# Expanding Diversity of Fused Steroid-Quinoline Hybrids by Sequential Amination/Annulation/Aromatization Reactions

**DOI:** 10.1021/acs.joc.4c02981

**Published:** 2025-03-07

**Authors:** Caterina Momoli, Antonio Arcadi, Marco Chiarini, Valerio Morlacci, Laura Palombi

**Affiliations:** † Dipartimento di Scienze Fisiche e Chimiche, Università degli studi dell’Aquila, Via Vetoio, Coppito (AQ), 67100 L’Aquila,Italy; ‡ Dipartimento di Bioscienze e Tecnologie Agroalimentari e Ambientali, Università degli studi di Teramo, Via R. Balzarini, 64110 Teramo, Italy

## Abstract

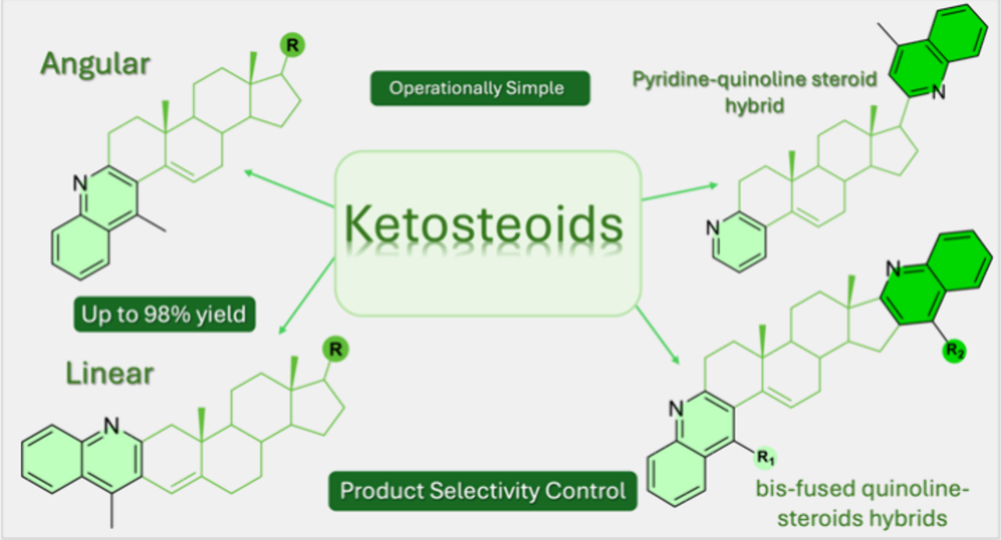

Viable alternative approaches to a variety of ring A and ring D-fused steroid-quinoline hybrids, along with ring A, D-fused, and/or ring A-fused, side chain-substituted steroid-bis-quinolines were explored by means of sequential amination/annulation/aromatization reactions of suitable ketosteroids with 2-acyl-substituted anilines. Key factors directing the chemoselective behavior of polyfunctionalized substrates were investigated. Remarkably, the use of TMSOTf as an alternative promoter/catalyst enabled the direct synthesis of the desired hybrids, avoiding the protection/deprotection steps of the conventional procedures when the starting substrates contained labile functional groups.

## Introduction

**Figure 1:**
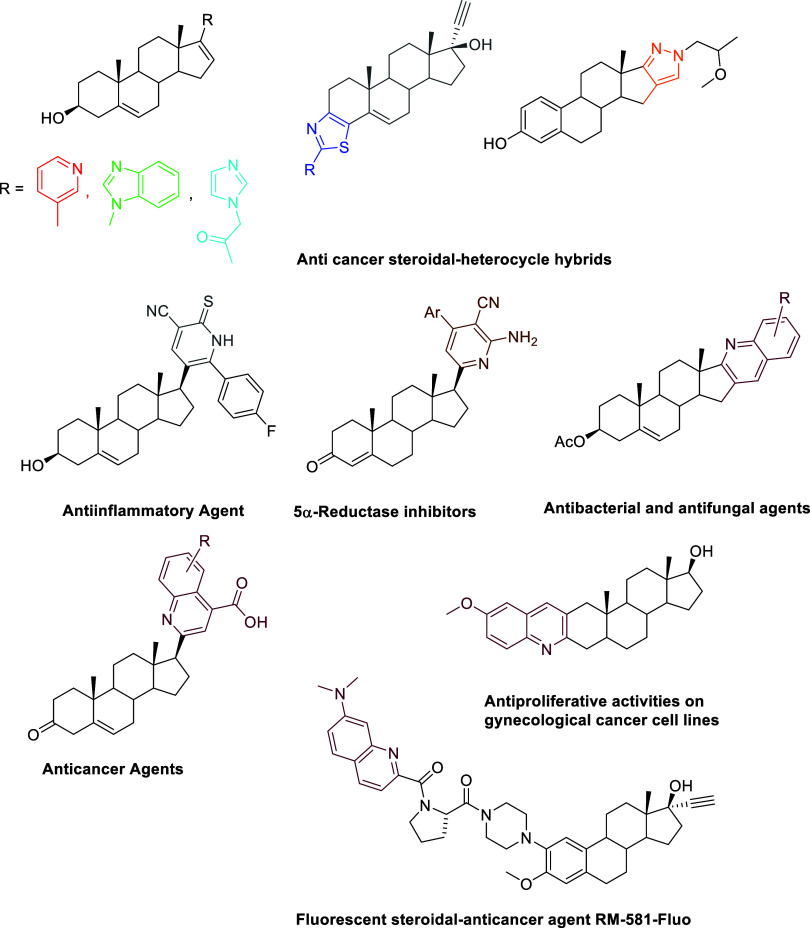
Selected steroid-heterocycle hybrids of pharmaceutical relevance.

Steroids, a class of naturally occurring biomolecules known for their wide range of biological activities, play a crucial role in the search for new drugs and represent the second-largest category in the global pharmaceutical market.^1^ The easy modification of the several functional groups in their skeleton makes steroids attractive substrates for different targets, improving their effectiveness and allowing for the modulation of their pharmacological profiles.^2^ Significant research efforts have focused on the rational modification of steroids,^3^ particularly in synthesizing and studying steroid-heterocycle hybrid compounds. These hybrids have gained attention for their potential to serve as novel scaffolds for therapeutic applications, as evidenced by numerous reports on their synthesis and biological activity (Figure 1).^4^

A steroid-oxazole-1,2′-[1,3]oxazete derivative was reported to exert a cardioprotective effect by increasing left ventricular pressure via kinase-2 inhibition, in addition to the biological activity on ischemia/reperfusion injury.^5^ Estrane derivatives annulated with five- and six-membered *N*-containing heterocycles have been synthesized and evaluated as chemotherapeutic anticancer agents.^6^ Analogously, steroidal pyrazole amides were tested for cytotoxicity^7^ as well as several 16-imidazolyl substituted steroidal derivatives.^8^ Estradiol derivatives with an annulated isooxazoline ring demonstrated antiproliferative activity against gynecological tumor cell lines (cervical, ovarian, and breast),^9^ while *in silico* simulations showed that 1,2,3-triazoles bonded to the steroid core possessed potential activity for the therapy of ovarian and colorectal cancer.^10^ Pregnenolone was used as a template to develop new anticancer compounds by means of heterocyclization reactions.^11^ It was settled that the incorporation of a pyrimidine scaffold into the steroid basic skeleton was crucial for the development of potent anticancer, antioxidant, antibacterial, and anti-Alzheimer agents.^12^ A series of steroidal derivatives were evaluated for their antifungal properties against *Candida* species, by using a steroid as the basic skeleton and a thiazolopyrimidine heterocycle as a pharmacophore in the D-ring.^13^ A variety of synthetic methods provided steroidal monocyclic pyridines showing antineuroinflammatory, antiviral, antiproliferative, and androgenic-anabolic activity, cytochrome P450 (CYP) 1B1 inhibition, and acute toxicity.^14^ The synthesis of steroid-fused and binary hybrids, including quinolines, pyridopyrimidines, imidazopyridines, spirocyclic imidazopyridines, pyrazolopyridines, thienopyridines, pyridinyl-thiazoles, and tetrazolopyridines, along with their various biological applications, has been reviewed.^15^ In particular, the design of novel steroid-quinoline hybrids is gaining increasing interest due to their diverse pharmacological applications.^16^ Cholesterol-quinoline hybrids also represent promising templates for the development of new drugs to deal with protein aggregation processes.^17^ Great attention is also devoted to the discovery of more effective quinoline-derived anticancer drugs.^18^ Concurrently, the development of efficient, practical, and versatile synthetic strategies aimed at achieving high structural diversity, selectivity, and functional group tolerance has gained significant interest.

As part of our ongoing research activity, we explored different synthetic methodologies to build structural diverse functionalized quinolines.^19^ In particular, cascade amination/cyclization/aromatization reactions of β-(2-aminophenyl)-α,β-ynones with ketones using metal or Bro̷nsted acid catalysis enabled access to linear and angular-fused quinoline-steroid hybrids.^20^ Given the importance of structural variations in the steroidal core for drug design, we decided to expand the diversity of fused steroid-quinoline hybrids by sequential amination/annulation/aromatization reactions of readily available 2-acyl-substituted anilines with various functionalized mono- and dioxo-steroids.

Therein, we report the results of our investigation.

## Results and Discussion

**Scheme 1:**
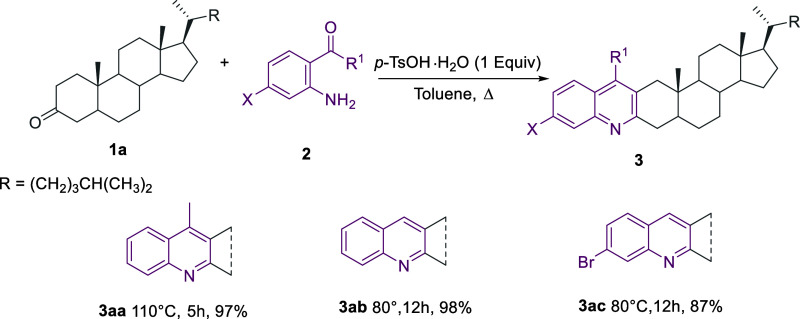
Synthesis of Ring A Linear-Fused Quinoline-Steroid Hybrids **3aa**–**3ac**

Based on our previous findings regarding the *p*-TsOH·H_2_O-mediated sequential reactions of β-(2-aminophenyl)-α,β-ynones with ketones,^20^ we envisaged that a suitable selection of the starting steroid would allow us to selectively obtain either the linear or angular-fused quinoline-steroid hybrids, which are fused at the C2/C3 and C3/C4 sides of the steroid A ring, respectively. Initially, we tried to expand the diversity of ring A-fused quinoline-steroids. As reported in Scheme 1, linear quinoline-steroid hybrids **3aa**–**3ac** were isolated in excellent yield by reacting 5α-cholestan-3-one **1a** with the 2-aminoaryl carbonyls **2a**-**2c** under Bro̷nsted acid-mediated conditions in toluene at 80 or 110 °C. The present protocol should represent an advantageous alternative to the conventional Vilsmeier–Haack chloroformylation^18g,21^ or deaminative coupling of 2-aminoaryl carbonyls^22^ with branched amines, according to the growing importance of more sustainable protocols.^23^ It is worth noting that, although the linear ring A-fused quinolino (3′,2′:2,3)cholestane derivative **3ab** was also obtained by heating 3-pyrrolidinocholest-2-ene with 2-aminobenzaldehyde **2b**, 2-aminoacetophenone **2a** was unreactive toward the enamine. Conventional basic conditions (potassium hydroxide in ethanol) and superbase-mediated indirect Friedländer reaction were also effective to produce the linear-fused quinoline derivative **3ab**.^24^ In this regard, it has been reported that the regioselectivity of the annulation depends on the configuration at the C-5 position. Specifically, 3-keto steroids with a 5α-configuration and a *trans* junction between rings A and B yielded exclusively linear annulated derivatives 3. In contrast, the corresponding 5β-derivatives with a *cis* junction favored the formation of angular products, albeit with lower regioselectivity.^25^ Anyway, only Δ^2^-enamine intermediates were isolated from 3-keto steroids with a 5α-configuration.^26^

**Scheme 2:**
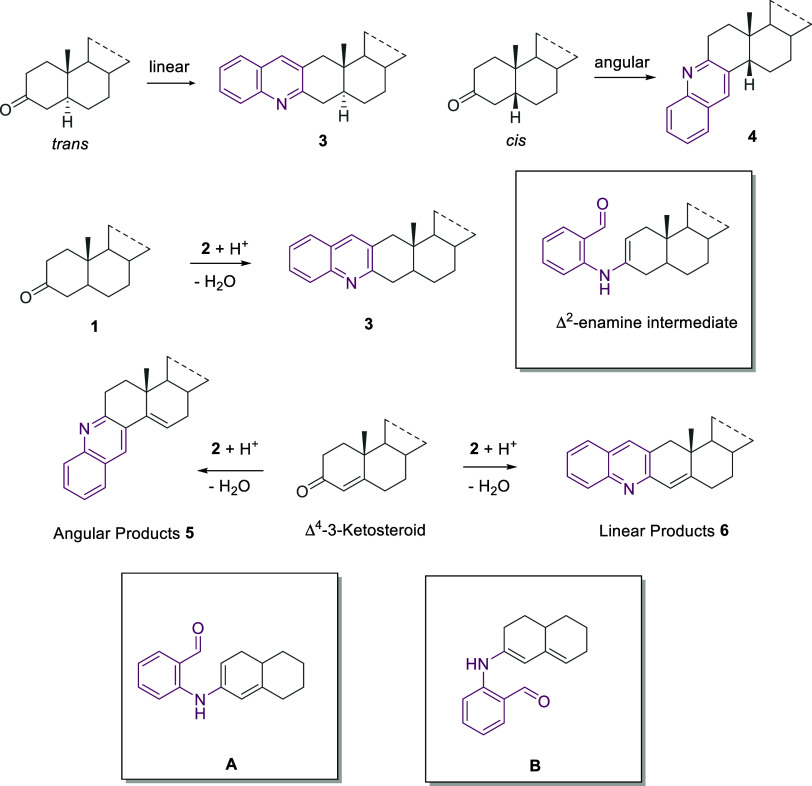
Ring A Linear- vs Angular-Fused Quinoline-Steroid hybrids

Conversely, Δ^4^-3-ketosteroids represented more suitable starting materials for the preparation of ring A angular-fused steroid-quinoline hybrids **5**. Calculations on a simplified model performed at the CAM-B3LYP/6-311+G* level of theory with the Gaussian program^27^ confirm that the dienamine regioisomer B, leading to the angular product **5**, is more stable than A (23 kJ/mol), which should instead afford the linear derivative **6** (Scheme 2). However, although Δ^4^-3-ketosteroids were also experimentally reported to undergo amination reactions, leading only to the corresponding Δ^3,5^-dienamine derivatives,^28^ the exploration of the reaction conditions showed that solvent, temperature, and features and loading of the catalyst play a key role in directing the sequential cascade toward the angular ring A-fused hybrid **5aa**.^29^

**Scheme 3:**
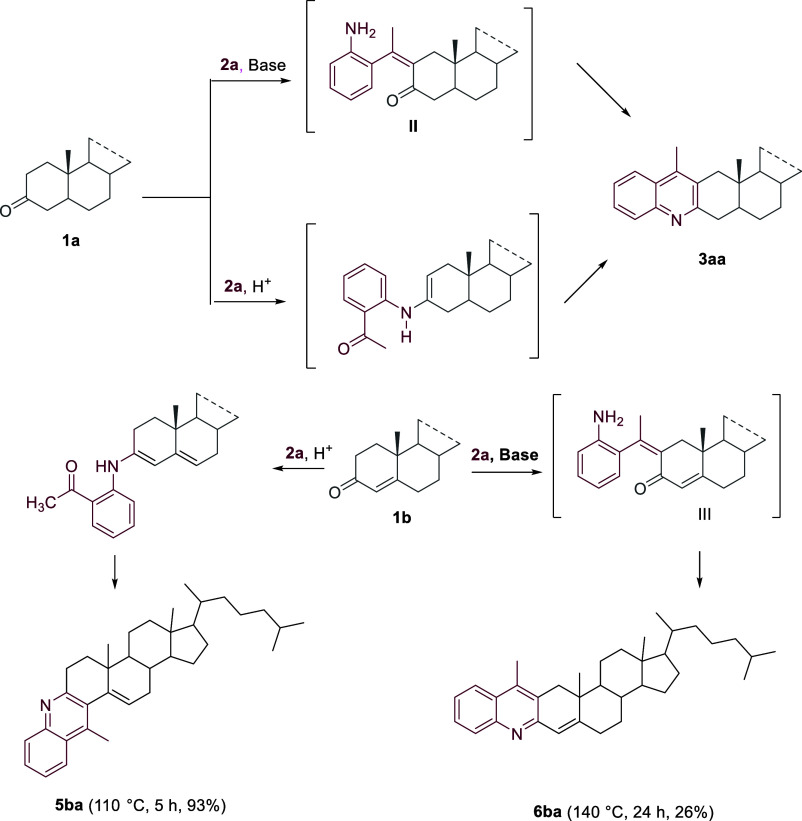
Sequential Amination/Annulation/Aromatization vs Sequential Intermolecular Aldol Reaction/Cyclization/Aromatization

Both the sequential amination/annulation/aromatization reaction and the sequential intermolecular aldol reaction/cycloamination/aromatization were supported for the well-known Friedländer acid- or base-promoted condensation of an aromatic 2-amino-substituted carbonyl compound with a carbonyl derivative containing a reactive a-methylene group (Scheme 3).^30^ Consequently, both sequential processes are expected to allow the selective formation of the linear ring A-fused steroidal quinoline derivative **3** under our acid and the reported basic-mediated conditions.^31^ Conversely, the choice of reaction conditions allowed us to address the angular-fused hybrid **5ba** under the *p*-TsOH·H_2_O (1 equiv)-promoted reaction of **1b** (1 equiv) with **2a** (1.1 equiv) in toluene at 110 °C. The observed excellent yield of **5ba** at the gram scale confirmed the practicality of our approach. Instead, the intermolecular aldol reaction/cycloamination/aromatization sequence leading to intermediate **III,** which exclusively occurs under *t-*BuOK-promoted conditions of the same reagents, gave only the linear-fused hybrid **6ba**, although in an unsatisfactory yield (Scheme 3).

**Scheme 4:**
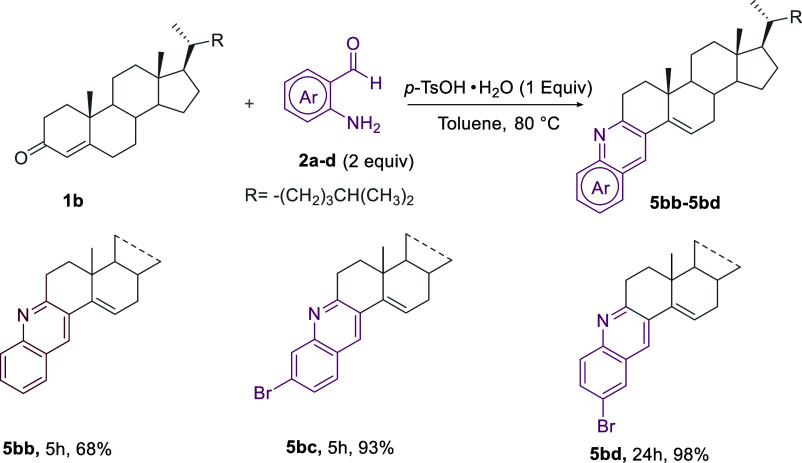
Extension of the Methodology to the Synthesis of Angular Ring A-Fused Quinoline-Steroid Hybrid **5bb–5bd** from the Reaction of Δ^4^-3-Ketosteroids **1b** with 2-Aminoaryl Carbonyls **2a**–**d**

Moreover, we are pleased to report that the sequential amination/cyclization/aromatization cascade process could also be extended to the straightforward synthesis of the 4-unsubstituted quinoline-steroid hybrids **5bb**–**5bd** in good to excellent yields by means of the Bro̷nsted acid-mediated reaction of **1b** with 2 equiv of 2-aminobenzaldehydes **2b**–**2d** (Scheme 4).

The methodology was also effective for the selective synthesis in an excellent yield of the angular derivative **5ca**. This unprecedented compound, having the free OH functionality, was directly obtained by reacting testosterone **1c** with **2a**. Under such conditions, only traces of the linear derivative **6ac** were observed.^18g,32^ No byproducts originating from the 2-amino-benzaldehyde self-condensation were observed.^33^

In a subsequent exploration of polyfunctionalized steroids bearing OH functionality, we were pleased to observe the smooth formation of the corresponding quinoline derivative **5da** in 67% yield from ethisterone **1d**, by using TMSOTf as a promoter.^34^ We failed to obtained the desired product both in the *p*-TsOH·H_2_O-mediated or the NaAuCl_4_·2H_2_O-catalyzed reaction.^35^

**Scheme 5:**
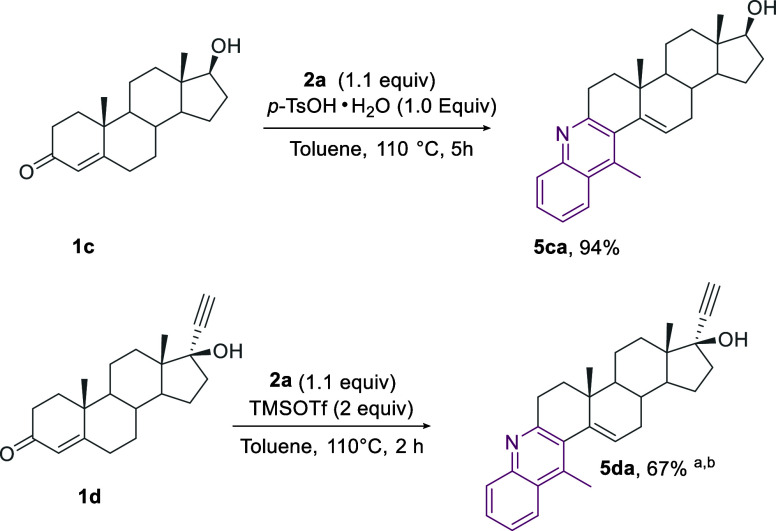
Synthesis of Angular-Fused Quinoline-Testosterone/Ethisterone Hybrids **5ca**–**5da** **5da** was isolated in 52% and **6da** in 6% yield when the reaction was carried out in the presence of 0.2 equiv of TMSOTf. Complex mixtures were obtained when the reaction was carried out in the presence of 1 equiv of *p*-TsOH· H_2_O or 0.05 equiv of NaAuCl_4_·2H_2_O.

Worth noting, ethisterone-based fused thiazole analogues exhibit potent anticancer activity at submicromolar concentrations.^36^ To the best of our knowledge, this is the first synthesis of ethisterone-based fused quinolines (Scheme 5).

Considering the potential of various heterocyclic fused derivatives in the development of effective anticancer agents, we extended our research by focusing on structural modifications of the steroid D-ring. In previous work, estrone 3-methyl ether was used for the synthesis of D-fused derivatives through combined chloroformylation/aminocyclization with anilines.^37^ More recently, a one-pot synthesis of D-fused estrone-quinoline hybrids was reported, using a dinuclear Ru(II) Schiff base complex to catalyze the acceptorless dehydrogenative coupling of estradiol with 2-nitrobenzyl alcohol.^31b^ Thus, we tested our conditions using 2-aminoacetophenone **2a** and estrone **1e**. Despite the challenges posed by the increased rigidity and steric hindrance of the D-ring,^38^ we were pleased to achieve a nearly quantitative conversion to the desired hybrid **7ea** when 2 equiv of the *p*-TsOH·H_2_O promoter were used. The key role of the acid promoter in the annulation step of the sequential amination/cyclization/aromatization was previously highlighted.^30a^

**Scheme 6:**
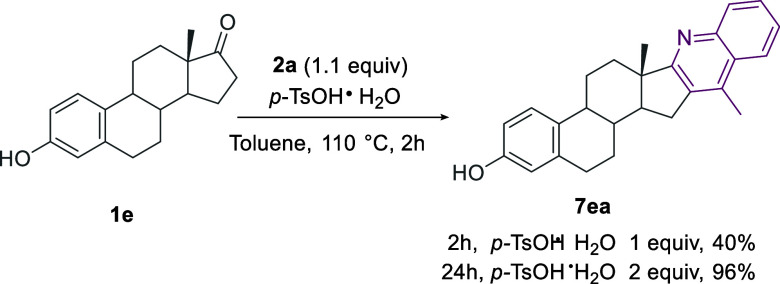
Synthesis of the Ring D-Fused Quinoline-Estrone Hybrid **7ea**

Notably, the tolerance toward the phenolic −OH group allowed for the direct formation of **7ea** (Scheme 6).

**Scheme 7:**
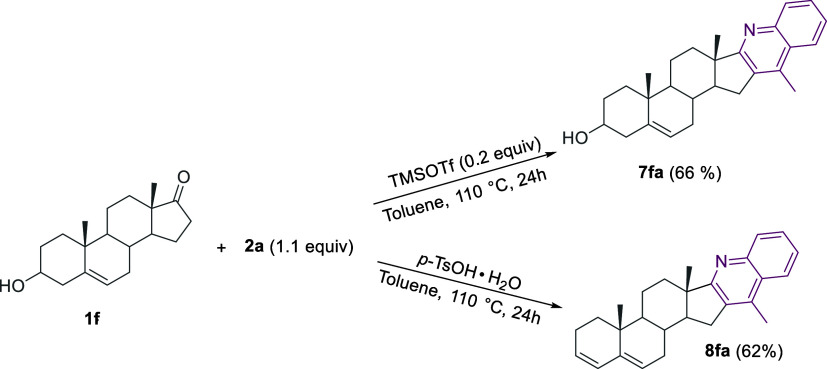
TMSOTf-Catalyzed vs *p*-TsOH·H_2_O-Promoted Reaction of the Epiandrosterone **1f** with the 2-Aminoacetophenone **2a**

By contrast, when performing the reaction on epiandrosterone **1f**, the expected hybrid **7fa** was isolated in good yield only by using 0.2 equiv of the Lewis acid TMSOTf as the catalyst, whereas the *p*-TsOH·H_2_O-mediated reaction afforded the product **8fa**. Very likely, the more reactive allylic alcohol functionality undergoes fast dehydration under these latter reaction conditions (Scheme 7).

**Scheme 8:**
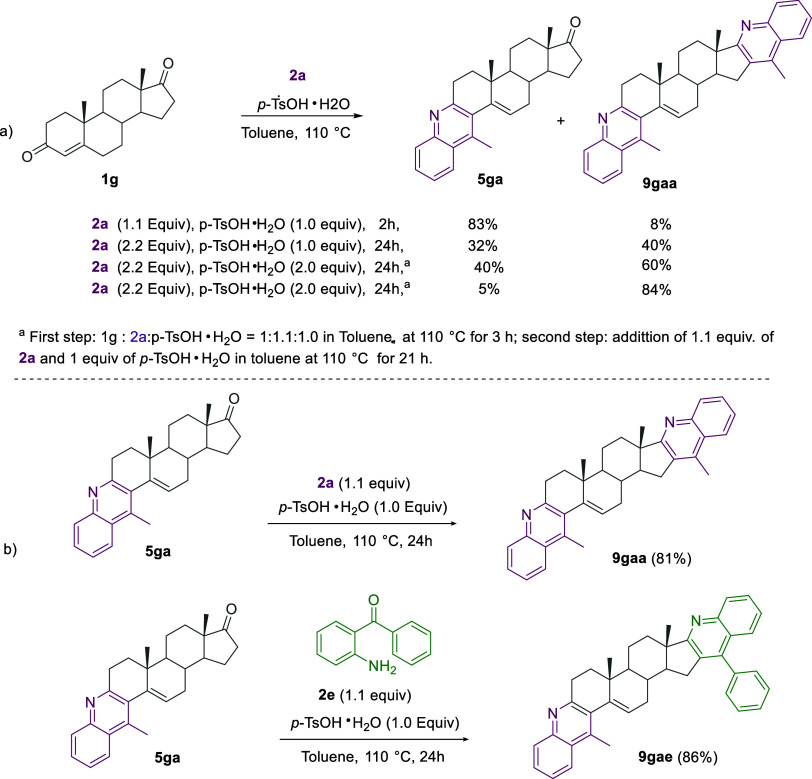
Synthesis of Mono-Fused Quinolines **5** and Bis-Fused Quinoline-Steroid Hybrids **9**

We subsequently tested 4-androstene-3,17-dione **1g** as a model substrate for double functionalization. As expected, the quinoline-steroid hybrid **5ga** could be selectively obtained in 83% yield following the usual treatment with a stoichiometric amount of *p*-TsOH·H_2_O for 2 h at 110 °C in the presence of 1.1 equiv of **2a** (Scheme 8a). However, we failed the selective formation of the bis-quinoline **9gaa** directly from the reaction of **1g** in the presence of an excess of 2.5 equiv of **2a** and 1.0 equiv of *p*-TsOH·H_2_O (one-pot procedure). Notably, **5ga** underwent smooth functionalization on ring D under the optimized conditions in the subsequent step. This latter approach allowed us to isolate the bis-quinoline-steroid hybrids **9gaa** and **9gae** in 81 and 86% yields, respectively. The one-pot procedure gave a mixture of the bis-quinoline **9gaa** (40% yield) alongside the monofunctionalized derivative **5ga** (32% yield). Pleasantly, the yield of **9gae** increased to 60% by using 2 equiv of *p*-TsOH·H_2_O instead of 1 equiv in the one-pot one procedure and to 84% yield by a combined one-pot two-step protocol (see SI) (Scheme 8b).

To the best of our knowledge, the synthesis of ring A, D-fused bis-quinoline hybrids has not been previously reported.

**Scheme 9:**
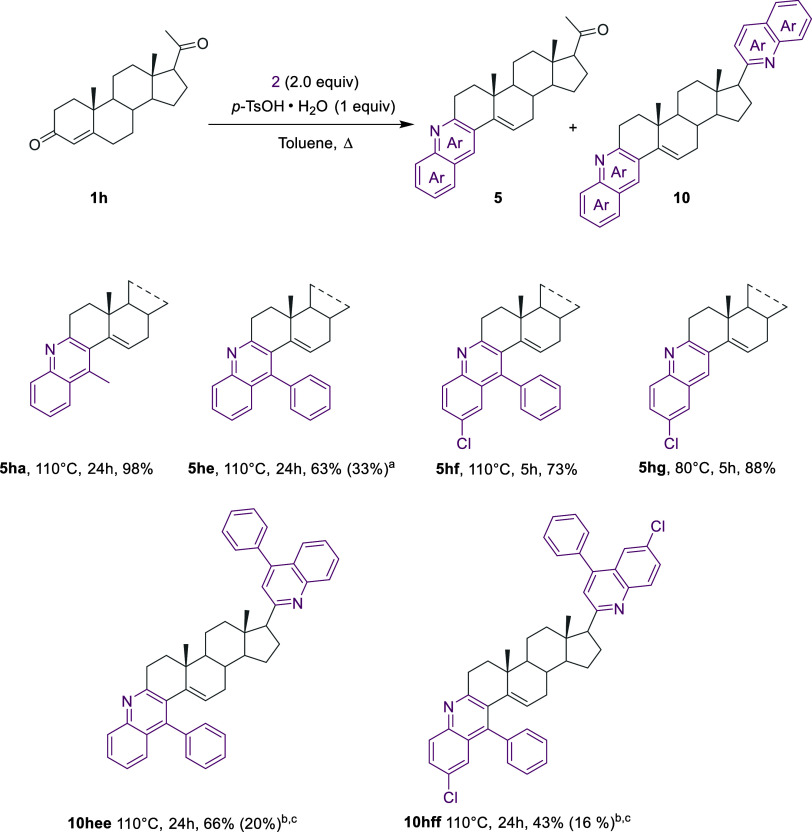
Synthesis of Mono-Fused Quinoline **5ha**–**5hg** and Bis-Fused Quinoline-Steroid Hybrids **10hee**–**10hff** Yield in parentheses refers to the bis-quinoline hybrid **10hee.** Combined one-pot two-step procedure. Yield in parentheses refers to the monoquinoline hybrid **5**.

The application of chemoselective Bro̷nsted acid-mediated amination/annulation/aromatization of the A ring of progesterone **1h** led to the corresponding angular-fused quinolines hybrids **5ha**, **5he**, **5hf**, and **5hg** due to the preferential amination over the conjugated carbonyl rather on the carbonyl in the side chain. However, valuable bis-quinolines **10hee** and **10hff** were obtained to a significant extent by the one-pot two-step protocol, prolonging the reaction times (Scheme 9).

**Scheme 10:**
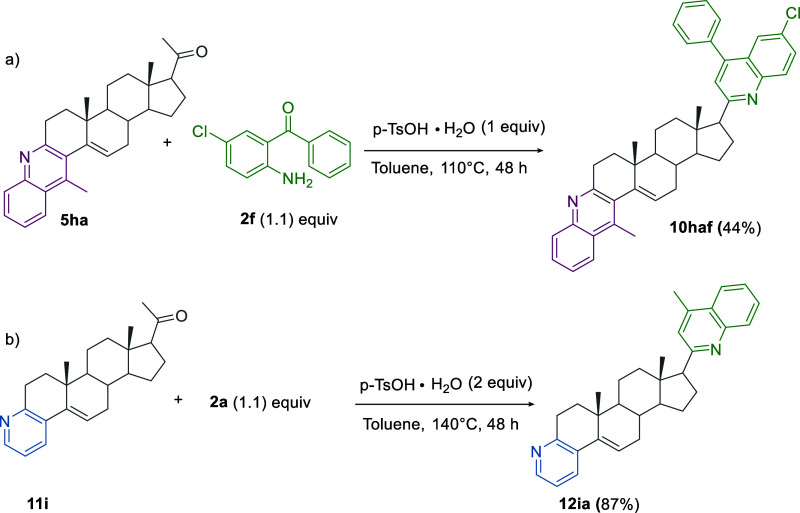
Synthesis of Different Ring A, D-Fused Heterocycle-Steroid Hybrids as Potential Prostate Cancer Therapy Agents

Moreover, bis-fused hybrids 10 with different quinoline moieties can also be easily obtained (Scheme 10a). It is worth highlighting the relevance for prostate cancer therapy of the availability of more effective inhibitors of the enzyme 17α-hydroxylase/17,20-lyase (CYP17 A1). The very limited therapy for PC makes the synthesis of new potential anticancer steroidal hybrids, differing either in the structure of the steroidal part or in the structure of nitrogen-containing heterocycles, highly relevant. Accordingly, we extended the procedure to the efficient synthesis of product **12ia** from the A-ring-fused pyridine-steroid hybrid 11i (Scheme 10b).^39^

**Scheme 11:**
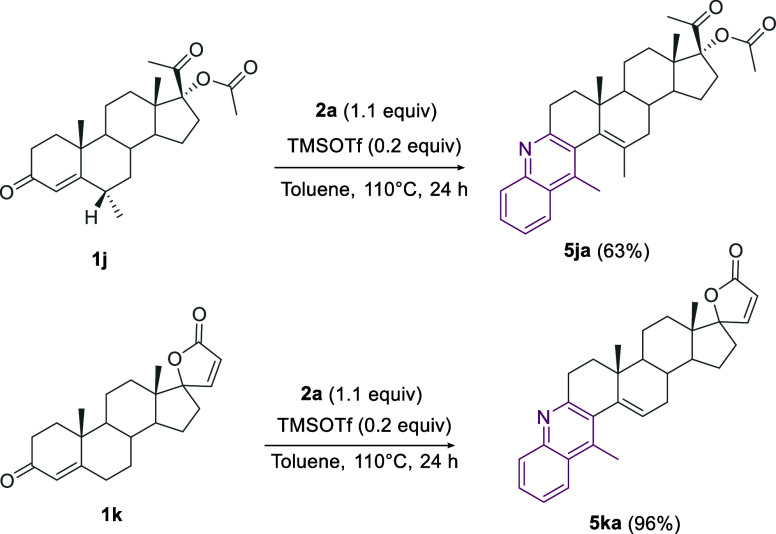
TMSOTf as Alternative Catalyst

Finally, we have to remark on the crucial role of TMSOTf as an alternative catalyst/promoter when the conventional acid or basic conditions are not applicable due to the instability of functional groups on the starting substrates (Scheme 11).

## Conclusions

Sequential amination/annulation/aromatization reactions of suitably functionalized ketosteroids with 2-acylanilines allowed the efficient expansion of the diversity of a variety of ring A and ring D-fused steroid-quinoline hybrids. Moreover, the procedure was extended to the synthesis of unprecedented ring A, D-fused steroid bis-quinolines and/or ring A, side chain-substituted steroid-bis-quinolines The conditions for achieving product selectivity control of polyfunctionalized steroid derivatives were deeply explored. The study of the substrate scope showed that the procedure could be applied to the straightforward synthesis of the 4-unsubstituted/4-functionalized quinoline-steroid hybrids in high yields by the *p*-TsOH·H_2_O-mediated reaction of Δ^4^-3-ketosteroids with the 2-aminobenzaldehydes or 2-aminoarylketones in toluene at 80 and 110 °C, respectively. The methodology was applied to the direct synthesis of the ring A angular-fused quinoline-testosterone hybrid bearing a free–OH functionality as well as to the selective functionalization at the 3-position of the Δ^4^-3,17-dione functionalized steroids. Moreover, our protocol allowed us to obtain the late-stage construction of the ring A and D-fused bis-quinoline-steroid hybrids through combined sequential processes. Remarkably, TMSOTf resulted in an alternative catalyst/promoter for the synthesis of polyfunctionalized steroids, which failed to occur under conventional conditions.

## Experimental Section

### General Materials and Methods

Flash chromatography was carried out using silica gel 60 (70–230 mesh, Merck, Darmstadt, Germany). Yields are usually given for isolated products showing one spot on a TLC plate, and no impurities were detectable in the NMR spectrum. ^1^H NMR spectra were recorded at 400.13 MHz on a Bruker Avance III spectrometer using the standard Bruker “zg30” sequence. Chemical shifts (in ppm) were referenced to CDCl_3_ (δ = 7.26 ppm) or DMSO-d6 (δ = 2.33 ppm) as an internal standard. ^13^C NMR spectra were taken on the same machine at 100.613 MHz, using the standard Bruker “zgpg30” proton-decoupled sequence. Carbon spectra were calibrated with CDCl_3_ (δ = 77.0 ppm) or DMSO-d6 (δ = 39.5 ppm) as an internal standard. Coupling constants (*J*) are quoted in Hertz. Mass measurements were performed using a MALDI-TOF spectrometer ABSCIEX TOF/TOF 5800, using a matrix in combination with KI for ionization, or the Thermo Fisher Orbitrap IQ-X Tribrid mass spectrometer. Unless otherwise stated, all starting materials, catalysts, and solvents were commercially available and were used as purchased. The steroidal derivatives **1k** and **11i** are known compounds and were prepared according to the literature and identified by comparison with the NMR spectra. Reaction products were purified by flash chromatography on silica gel (60–200 μm) by elution with *n*-hexane/EtOAc mixtures.

#### Synthesis of 10,13-Dimethyl-1,6,9,10,11,12,13,14,15,16-dodecahydro-5′*H*-spiro[cyclopenta[*a*]phenanthrene-17,2′-furan]-3,5′-dione (**1k**)^40^

Tributylamine (0.86 mL, 3.7 mmol) and Pd(OAc)_2_(PPh_3_)_2_ (0.017 g, 0.022 mmol) were added to a stirred solution of ((8*R*,9*S*,10*R*,13*S*,14*S*,17*S*)-17-hydroxy-10,13-dimethyl-3-oxo-2,3,6,7,8,9,10,11,12,13,14,15,16,17-tetradecahydro-*1H*-cyclopenta[a]phenanthren-17-yl)-propynoic acid methyl ester (0.407 g, 1.1 mmol) in DMF (2 mL). The mixture was purged with nitrogen, and formic acid (0.11 mL, 2.9 mmol) was added all at once. The mixture was stirred at 60 °C under a nitrogen atmosphere for 6h, AcOEt, and 0.1 N HCl were added, and the organic layer was separated, dried (Na_2_SO_4_), and concentrated at reduced pressure. The residue was purified by flash chromatography. Elution with an *n*-hexane/EtOAc 85/15 mixture afforded 10,13-dimethyl-1,6,9,10,11,12,13,14,15,16-dodecahydro-5*′H*-spiro[cyclopenta[*a*]phenanthrene-17,2*′*-furan]-3,5′-dione (**1k**) (0.303g, 81% yield) as a white solid, ^1^H NMR (400 MHz, CDCl_3_) δ 7.45 (d, *J* = 5.7 Hz, 1H), 5.97 (d, *J* = 5.7 Hz, 1H), 5.77–5.74 (m, 1H), 2.81–2.18 (m, 5H), 2.10–1.32 (m, 12H), 1.22 (s, 3H), 1.17–0.89 (m, 5H) ppm.

#### General Procedure **A** for the Synthesis of Polycyclic Quinoline-Fused Steroids **3aa**, **5ba**, **5ca**, **5ga**, **5ha**, **5he**, **5hf**, **7ea**, **8fa**, **9gae**, **10haf**, **10hee**, and **10hff**

To a solution of ketosteroid **1** (0.4 mmol, 0.13M) in toluene (3 mL) was added *p*-toluenesulfonic acid monohydrate (0.4 mmol, 1 equiv) and 2-aminoacetophenone **2** (from 0.44 to 0.80 mmol, from 1.1 to 2 equiv). After being stirred at 110 °C for between 5 and 24 h in an oil bath, the mixture was extracted with CH_2_Cl_2_ and a saturated solution of NaHCO_3_ (3 × 15 mL). The crude was loaded onto a chromatographic column of silica gel and eluted with a hexane/ethyl acetate mixture (from 9:1 to 8:2).

#### General Procedure **B** for the Synthesis of Polycyclic Quinoline-Fused Steroids **3ab**, **3ac**, **5bb**, **5bc**, **5bd**, and **5hg**

To a solution of ketosteroid **1** (0.4 mmol, 0.13M) in toluene (3 mL) was added *p*-toluenesulfonic acid monohydrate (0.4 mmol, 1 equiv) and 2-aminobenzaldehyde **2** (0.8 mmol, 2 equiv). After being stirred at 80 °C for between 5 and 24 h in an oil bath, the mixture was extracted with CH_2_Cl_2_ and a saturated solution of NaHCO_3_ (3 × 15 mL). The crude product was loaded onto a chromatographic column of silica gel and eluted with a hexane/ethyl acetate mixture (from 9:1 to 8:2).

#### General Procedure **C** for the Synthesis of Polycyclic Quinoline-Fused Steroids **5da**, **5ja**, **5ka**, and **7fa**

To a solution of ketosteroid **1** (0.4 mmol, 0.13M) in toluene (3 mL) was added TMSOTf (from 0.08 to 0.8 mmol, from 0.20 to 2 equiv) and 2-aminoacetophenone **2** (0.44 mmol, 1.1 equiv). After being stirred at 110 °C for between 5 and 48 h in an oil bath, the mixture was extracted with CH_2_Cl_2_ and H_2_O (3 × 15 mL). The crude was loaded onto a chromatographic column of silica gel and eluted with a hexane/ethyl acetate mixture (from 9:1 to 8:2).

#### General Procedure **D** One-Pot Two-Step for the Synthesis of Polycyclic Quinoline-Fused Steroid **9gaa**

To a solution of ketosteroid **1g** (0.4 mmol, 0.114 g, 0.13M) in toluene (3 mL) was added *p*-toluenesulfonic acid monohydrate (0.4 mmol, 0.076 g, 1 equiv) and 2-aminoacetophenone **2a** (0.44 mmol, 0.060 g, 1.1 equiv), and the reaction mixture was stirred at 110 °C for 3 h in an oil bath, monitoring progress periodically by TLC. After complete conversion of the starting material, a second equivalent of *p*-toluenesulfonic acid monohydrate (0.4 mmol, 0.076 g, 1 equiv) and 2-aminoacetophenone **2a** (0.44 mmol, 0.060 g 1.1 equiv) were added, and the reaction was stirred at 110 °C in the oil bath overnight. The mixture was then extracted with CH_2_Cl_2_ and a saturated NaHCO_3_ solution (3 × 15 mL). The crude product was loaded onto a silica gel chromatographic column and eluted with a hexane/ethyl acetate mixture (9:1 to 8:2). The product **9gaa** was obtained as a white solid in 83% yield (0.33 mmol, 0.163 g).

#### Procedure **E** for the Synthesis of the Polycyclic Quinoline-Fused Steroid **6ba**

To a solution of ketosteroid **1b** (0.78 mmol, 0.3 g, 0.39M) in toluene (2 mL) was added *t*-BuOK (0.78 mmol, 0.087 g, 1 equiv) and 2-aminoacetophenone **2a** (0.78 mmol, 0.094 g, 1.0 equiv). After being stirred at 140 °C in an oil bath for 24 h, the reaction mixture was cooled to room temperature, and 3.0 mL of ethyl acetate was added and concentrated in vacuo. The crude product was loaded onto a chromatographic column of silica gel and eluted with a hexane/ethyl acetate mixture 97/3. The product **6ba** was obtained as a white solid liquid in 26% yield (0.2 mmol, 98 mg).

#### Procedure for the Large-Scale Reaction

To a solution of ketosteroid **1b** (2.6 mmol, 1.0 g, 0.13M) in toluene (20 mL) was added *p*-toluenesulfonic acid monohydrate (2.6 mmol, 0.49 g, 1 equiv) and 2-aminoacetophenone **2a** (2.86 mmol, 0.35 g, 1.1 equiv). After being stirred at 110 °C in an oil bath, the mixture was extracted with CH_2_Cl_2_ and a saturated solution of NaHCO_3_ (3 × 30 mL). The crude was loaded onto a chromatographic column of silica gel and eluted with a hexane/ethyl acetate mixture (from 9:1 to 8:2). The product **5ba** was obtained as a white solid in 93% yield (2.42 mmol, 1.169 g).

#### (1*R*,13a*S*,15a*R*)-12,13a,15a-Trimethyl-1-((*R*)-6-methylheptan-2-yl)-2,3,3a,3b,4,5,5a,6,13,13a,13b,14,15,15a-tetradecahydro-1H-cyclopenta[5,6]naphtho[1,2-*b*]acridine (**3aa**)

Prepared from **1a** (0.4 mmol, 155 mg) and **2a** (0.44 mmol, 59 mg) following general procedure **A** and isolated as a white solid in 97% yield (0.039 mmol, 194 mg); ^**1**^**H NMR** (400 MHz, CDCl_3_) δ 7.98–7.95 (m, 2H), 7.61–7.57 (m, 1H), 7.46–7.42 (m, 1H), 3.04–2.97 (m, 2H), 2.89–2.76 (m, 1H), 2.55 (s, 3H), 2.37–2.32 (m, 1H), 2.09–2.07 (m, 1H), 1.92–0.79 (m, 24H), 0.96 (d, *J* = 6.5 Hz, 3H), 0.91 (d, *J* = 6.6 Hz, 3H), 0.90 (d, *J* = 6.6 Hz, 3H), 0.81 (s, 3H), 0.72 (s, 3H) ppm. ^**13**^**C {**^**1**^**H} NMR** (101 MHz, CDCl_3_) δ: 157.6, 146.0, 141.7, 129.0, 128.1, 127.9, 126.9, 125.2, 123.3, 56.4, 56.3, 54.0, 42.5, 41.4, 41.3, 40.0, 39.6, 37.9, 36.2, 35.9, 35.5, 35.0, 31.6, 28.6, 28.3, 28.1, 24.3, 23.9, 22.9, 22.6, 21.4, 18.8, 13.7, 12.06, 12.04 ppm. HRMS (ESI-Orbitrap) Calcd for C_35_H_52_N [M + H]^+^ 486.4094; Found: 486.4095.

#### (1*R*,13a*S*,15a*R*)-13a,15a-Dimethyl-1-((R)-6-methylheptan-2-yl)-2,3,3a,3b,4,5,5a,6,13,13a,13b,14,15,15a-tetradecahydro-1H-cyclopenta[5,6]naphtho[1,2-*b*]acridine (**3ab**)^24^

Prepared from **1a** (0.4 mmol, 155 mg) and **2b** (0.8 mmol, 97 mg) following general procedure **B** and isolated as a white solid in 98% yield (0.39 mmol, 190 mg); mp:217–218 °C; ^1^H NMR (400 MHz, CDCl_3_) δ 8.18 (s, 1H), 7.78 (s, 2H), 7.61–7.49 (m, 2H), 3.12–3.01 (m, 1H), 2.99–2.95 (m, 1H), 2.84–2.72 (m, 1H), 2.58–2.55 (d, *J* = 16.1 Hz, 1H), 2.09–2.05 (m, 1H), 1.90–0.99 (m, 24H), 0.95 (d, *J* = 6.5 Hz, 3H), 0.89 (dd, *J* = 6.6, 1.8 Hz, 6H), 0.80 (d, *J* = 1.4 Hz, 3H), 0.71 (s, 3H) ppm. ^13^C {^1^H} NMR (101 MHz, CDCl_3_) δ: 159.8, 147.1, 135.8, 130.9, 130.6, 129.1, 128.2, 125.8, 122.5, 56.4, 56.3, 53.5, 43.5, 42.5, 42.1, 40.0, 39.6, 37.4, 36.2, 35.9, 35.6, 35.2, 31.6, 28.7, 28.3, 28.1, 24.3, 23.9, 22.9, 22.7, 21.4, 18.8, 12.1, 11.7 ppm. HRMS (ESI-Orbitrap) Calcd for C_34_H_50_N [M + H]^+^ 472.3938; Found: 472.3938.

#### (1*R*,13a*S*,15a*R*)-9-Bromo-13a,15a-dimethyl-1-((R)-6-methylheptan-2-yl)-2,3,3a,3b,4,5,5a,6,13,13a,13b,14,15,15a-tetradecahydro-1H-cyclopenta[5,6]naphtho[1,2-*b*]acridine (**3ac**)

Prepared from **1a** (0.4 mmol, 155 mg) and **2c** (0.8 mmol, 160 mg) following general procedure **B** and isolated as a white solid in 87% yield (0.35 mmol, 196 mg); ^1^H NMR (400 MHz, CDCl_3_) δ 8.17–8.15 (m, 1H), 7.76 (s, 1H), 7.58–7.47 (m, 2H), 3.10–3.01 (m, 1H), 2.96–2.92 (m, 1H), 2.82–2.70 (m, 1H), 2.54–2.50 (m, 1H), 2.08–2.02 (m, 1H), 1.92–0.87 (m, 33H), 0.78 (s, 3H), 0.70 (s, 3H) ppm. ^13^C {^1^H} NMR (101 MHz, CDCl_3_) δ: 159.7, 147.2, 135.6, 130.8, 130.6, 129.0, 128.1, 125.8, 122.4, 56.4, 56.3, 53.5, 43.5, 42.5, 42.1, 39.9, 39.5, 37.4, 36.2, 35.8, 35.5, 35.2, 31.6, 28.7, 28.3, 28.0, 24.2, 23.9, 22.9, 22.6, 21.3, 18.7, 12.0, 11.7 ppm. HRMS (ESI-Orbitrap) Calcd for C_34_H_49_BrN [M + H]^+^ 550.3043; Found: 550.3047.

#### (3a*R*,5b*R*)-3a,5b,13-Trimethyl-3-((R)-6-methylheptan-2-yl)-2,3,3a,4,5,5a,5b,6,7,15,15a,15b-dodecahydro-1H-cyclopenta[5,6]naphtho[2,1-*a*]acridine (**5ba**)^29^

Prepared from **1b** (0.4 mmol, 154 mg) and **2a** (0.44 mmol, 60 mg) following general procedure **A** and isolated as a white solid in 98% yield (0.39 mmol, 189 mg); mp: 196–198 °C; ^1^H NMR (400 MHz, CDCl_3_) δ 8.01–7.95 (m, 2H), 7.63–7.59 (m, 1H), 7.51–7.46 (m, 1H), 5.60–5.58 (m, 1H), 2.88–2.77 (m, 2H), 2.69 (s, 3H), 2.33–2.26 (m, 1H), 2.17–2.06 (m, 2H), 1.91–1.00 (m, 21H), 0.94 (d, *J* = 6.5 Hz, 3H), 0.91 (s, 3H), 0.88 (d, *J* = 6.6 Hz, 3H), 0.87 (d, *J* = 6.6 Hz, 3H), 0.76 (s, 3H) ppm. ^13^C {^1^H} NMR (101 MHz, CDCl_3_) δ: 161.3, 145.6, 139.7, 139.2, 132.4, 128.9, 128.6, 128.2, 128.1, 125.3, 124.3, 56.8, 56.1, 47.2, 42.5, 39.9, 39.5, 37.9, 36.2, 35.8, 34.5, 32.3, 32.2, 32.0, 28.3, 28.0, 24.2, 23.9, 23.7, 22.9, 22.6, 22.0, 18.7, 15.5, 12.0 ppm. HRMS (MALDI-TOF) Calcd for C_35_H_50_N [M + H]^+^ 484.3938; Found: 484.3941.

#### (3a*R*,5b*R*)-3a,5b-Dimethyl-3-(6-methylheptan-2-yl)-2,3,3a,4,5,5a,5b,6,7,15,15a,15b-dodecahydro-1H-cyclopenta[5,6]naphtho[2,1-*a*]acridine (**5bb**)

Prepared from **1b** (0.4 mmol, 154 mg) and **2b** (0.8 mmol, 97 mg) following general procedure **B** and isolated as a white solid in 55% yield (0.22 mmol, 103 mg); mp:189–192 °C; ^1^H NMR (400 MHz, CDCl_3_) δ 8.21 (s, 1H), 8.00–7.96 (m, 1H), 7.78–7.74 (m, 1H), 7.64–7.58 (m, 1H), 7.46–7.42 (m, 1H), 6.32–6.30 (m, 1H), 3.30–3.10 (m, 2H), 2.35–2.21 (m, 2H), 2.11 (m, 1H), 1.94–1.81 (m, 2H), 1.73–1.07 (m, 19H), 1.01 (s, 3H), 0.97–0.96 (m, 3H), 0.91–0.89 (m, 6H), 0.76 (s, 3H) ppm. ^13^C {^1^H} NMR (101 MHz, CDCl_3_) δ: 157.2, 147.1, 140.1, 131.2, 130.0, 128.8, 128.1, 127.52, 127.5, 125.6, 123.1, 56.7, 56.2, 49.2, 42.4, 39.8, 39.5, 36.2, 35.8, 35.8, 34.4, 32.9, 31.5, 29.9, 28.3, 28.0, 24.3, 23.9, 22.8, 22.6, 21.3, 18.8, 18.8, 12.0 ppm. HRMS (MALDI-TOF) Calcd for C_34_H_48_N [M + H]^+^ 470.3787; Found: 470.3782.

#### (3a*R*,5b*R*)-12-Bromo-3a,5b-dimethyl-3-(6-methylheptan-2-yl)-2,3,3a,4,5,5a,5b,6,7,15,15a,15b-dodecahydro-1H-cyclopenta[5,6]naphtho[2,1-*a*]acridine (**5bc**)

Prepared from **1b** (0.4 mmol, 154 mg) and **2c** (0.44 mmol,160 mg) following general procedure B and isolated as a white solid in 93% yield (0.39 mmol, 178 mg); ^1^H NMR (400 MHz, CDCl_3_) δ 8.19–8.15 (m, 2H), 7.64–7.61 (m, 1H), 7.55–7.50 (m, 1H), 6.34–6.30 (m, 1H), 3.28–3.06 (m, 2H), 2.35–2.20 (m, 2H), 2.14–2.06 (m, 1H), 1.95–1.80 (m, 2H), 1.77–1.51 (m, 6H), 1.47–1.07 (m, 13H), 1.00 (s, 3H), 0.97 (m, 3H), 0.900 (d, *J* = 6.6 Hz, 3H), 0.896 (d, *J* = 6.6 Hz, 3H), 0.76 (s, 3H) ppm. ^13^C {^1^H} NMR (101 MHz, CDCl_3_) δ: 158.4, 147.5, 139.8, 130.9, 130.5, 130.4, 129.1, 128.8, 126.1, 123.7, 122.7, 56.7, 56.2, 49.2, 42.4, 39.7, 39.5, 36.2, 35.83, 35.79, 34.2, 32.9, 31.5, 29.9, 28.3, 28.0, 24.3, 23.9, 22.9, 22.6, 21.3, 18.9, 18.7, 12.0 ppm. HRMS (MALDI-TOF) Calcd for C_34_H_47_BrN [M + H]^+^ 548.2886; Found: 548.2886.

#### (3a*R*,5b*R*)-11-Bromo-3a,5b-dimethyl-3-(6-methylheptan-2-yl)-2,3,3a,4,5,5a,5b,6,7,15,15a,15b-dodecahydro-1H-cyclopenta[5,6]naphtho[2,1-*a*]acridine (**5bd**)

Prepared from **1b** (0.4 mmol, 154 mg) and **2e** (0.8 mmol, 160 mg) following general procedure **B** and isolated as a white solid in 98% yield (0.39 mmol, 215 mg); ^1^H NMR (400 MHz, CDCl_3_) δ 8.11 (s, 1H), 7.93–7.92 (m, 1H), 7.85–7.81 (m, 1H), 7.69–7.65 (m, 1H), 6.34–6.30 (m, 1H), 3.31–3.04 (m, 2H), 2.36–2.20 (m, 2H), 2.14–2.07 (m, 1H), 2.00–1.83 (m, 2H), 1.74–1.10 (m, 19H), 1.01 (s, 3H), 0.98–0.95 (m, 3H), 0.91–0.88 (m, 6H), 0.76 (s, 3H) ppm. ^13^C {^1^H} NMR (101 MHz, CDCl_3_) δ: 157.9, 146.0, 139.8, 132.1, 130.9, 130.0, 129.9, 129.4, 128.7, 124.1, 119.2, 56.7, 56.2, 49.2, 42.4, 39.7, 39.5, 36.2, 35.8 (2C), 34.2, 32.9, 31.5, 29.9, 28.3, 28.0, 24.3, 23.9, 22.8, 22.6, 21.3, 18.9, 18.7, 12.0 ppm. HRMS (MALDI-TOF) Calcd for C_34_H_47_BrN [M + H]^+^ 548.2886; Found: 548.2884.

#### (3*S*,3a*S*,5b*R*)-3a,5b,13-Trimethyl-2,3,3a,4,5,5a,5b,6,7,15,15a,15b-dodecahydro-1H-cyclopenta[5,6]naphtho[2,1-*a*]acridin-3-ol (**5ca**)

Prepared from **1c** (0.4 mmol, 115 mg) and **2a** (0.44 mmol, 60 mg) following general procedure **A** and isolated as a white solid in 94% yield (0.38 mmol, 146 mg); ^1^H NMR (400 MHz, CDCl_3_) δ 8.11–7.88 (m, 2H), 7.66–7.60 (m, 1H), 7.53–7.57 (m, 1H), 5.61–5.58 (m, 1H), 3.73 (t, *J* = 8.5 Hz, 1H), 2.95–2.78 (m, 2H), 2.71 (s, 3H), 2.37–2.27 (m, 2H), 2.23–2.07 (m, 2H), 1.94–1.88 (m, 1H), 1.85–1.02 (m, 11H), 0.94 (s, 3H), 0.86 (s, 3H) ppm. ^13^C {^1^H} NMR (101 MHz, CDCl_3_) δ: 161.2, 145.7, 139.8, 139.3, 132.3, 129.0, 128.24, 128.23, 128.1, 125.4, 124.3, 81.6, 51.5, 47.5, 43.0, 38.0, 36.8, 34.6, 32.3, 32.2, 31.6, 30.6, 23.7, 23.4, 21.7, 15.5, 11.3 ppm. HRMS (MALDI-TOF) Calcd for C_27_H_34_NO [M + H]^+^388.2635; Found: 388.2635.

#### 3-Ethynyl-3a,5b,13-trimethyl-2,3,3a,4,5,5a,5b,6,7,15,15a,15b-dodecahydro-1H-cyclopenta[5,6]naphtho[2,1-*a*]acridin-3-ol (**5da**)

Prepared from **1d** (0.4 mmol, 125 mg), **2a** (0.44 mmol, 60 mg) and TMSOTf (0.8 mmol, 146 mg) following general procedure **C** and isolated as a white solid in 67% yield (0.27 mmol, 110 mg); mp:184–187 °C; ^1^H NMR (400 MHz, CDCl_3_) δ 8.03–7.96 (m, 2H), 7.65–7.61 (m, 1H), 7.52–7.48 (m, 1H), 5.61–5.59 (m, 1H), 2.89–2.79 (m, 2H), 2.70 (s, 3H), 2.60 (s, 1H), 2.38–1.30 (m, 2H), 2.17 (dt, *J* = 13.5, 5.0 Hz, 1H), 2.09–2.02 (m, 1H), 1.87–1.25 (m, 12H), 0.95 (s, 3H), 0.93 (s, 3H) ppm. ^13^C {^1^H} NMR (101 MHz, CDCl_3_) δ: 161.1, 145.4, 139.7, 139.4, 132.2, 128.8, 128.3, 128.1, 128.0, 125.4, 124.3, 87.5, 79.7, 74.0, 50.8, 47.0, 46.8, 39.0, 37.9, 34.5, 32.8, 32.7, 32.1, 31.5, 23.7, 23.1, 21.7, 15.5, 12.8 ppm. HRMS (MALDI-TOF) Calcd for C_29_H_34_NO [M + H]^+^ 412.2635; Found: 412.2637.

#### (3a*S*,5b*R*)-3a,5b,13-Trimethyl-1,2,3a,4,5,5a,5b,6,7,15,15a,15b-dodecahydro-3H-cyclopenta[5,6]naphtho[2,1-*a*]acridin-3-one (**5ga**)

Prepared from **1g** (0.4 mmol, 115 mg) and **2a** (0.44 mmol, 60 mg) following general procedure **A** and isolated as a white solid in 84% yield (0.34 mmol, 133 mg); dec at 207 °C; ^1^H NMR (400 MHz, CDCl_3_) δ 7.98–7.93 (m, 2H), 7.62–7.58 (m, 1H), 7.49–7.45 (m, 1H), 5.59–5.58 (m, 1H), 2.87–2.75 (m, 2H), 2.67 (s, 3H), 2.57–2.37 (m, 3H), 2.15–2.06 (m, 2H), 2.00–1.78 (m, 5H), 1.64–1.31 (m, 5H), 0.92–0.91 (m, 6H) ppm. ^13^C {^1^H} NMR (101 MHz, CDCl_3_) δ: 220.8, 161.0, 145.7, 140.0, 139.3, 132.0, 129.0, 128.4, 128.0, 127.7, 125.4, 124.4, 51.8, 47.8, 47.4, 38.1, 35.9, 34.5, 32.2, 31.8, 31.6, 30.8, 23.7, 21.8, 21.4, 15.5, 13.7 ppm. HRMS (MALDI-TOF) Calcd for C_27_H_32_NO [M + H]^+^ 386.2478; Found: 386.2477.

#### 1-((3a*S*,5b*R*)-3a,5b,13-Trimethyl-2,3,3a,4,5,5a,5b,6,7,15,15a,15b-dodecahydro-1H-cyclopenta[5,6]naphtho[2,1-*a*]acridin-3-yl)ethan-1-one (**5ha**)

Prepared from **1h** (0.4 mmol, 126 mg) and **2a** (0.44 mmol, 60 mg) following general procedure **A** and isolated as a white solid in 98% yield (0.39 mmol, 162 mg); dec at 205 °C; ^1^H NMR (400 MHz, CDCl_3_) δ 7.97–7.91 (m, 2H), 7.61–7.57 (m, 1H), 7.47–7.44 (m, 1H), 5.55–5.54 (m, 1H), 2.83–2.79 (m, 2H), 2.65 (s, 3H), 2.54–2.50 (m, 1H), 2.30–2.05 (m, 7H), 1.75–1.18 (m, 11H), 0.87 (s, 3H), 0.65 (s, 3H) ppm. ^13^C {^1^H} NMR (101 MHz, CDCl_3_) δ: 209.3, 160.9, 145.5, 139.5, 139.1, 132.0, 128.8, 128.1, 128.1, 127.9, 125.2, 124.2, 63.4, 56.8, 47.0, 44.0, 38.8, 37.8, 34.4, 32.1, 32.0, 31.6, 31.5, 24.2, 23.5, 22.7, 21.9, 15.4, 13.3 ppm. HRMS (MALDI-TOF) Calcd for C_29_H_36_NO [M + H]^+^414.2791; Found: 414.2794.

#### 1-((3a*S*,5b*R*)-3a,5b-Dimethyl-13-phenyl-2,3,3a,4,5,5a,5b,6,7,15,15a,15b-dodecahydro-1H-cyclopenta[5,6]naphtho[2,1-*a*]acridin-3-yl)ethan-1-one (**5he**)

Prepared from **1h** (0.4 mmol, 126 mg) and **2e** (0.44 mmol, 87 mg) following general procedure **A** and isolated as a white solid in 63% yield (0.25 mmol, 120 mg); mp:197–199 °C; ^1^H NMR (400 MHz, CDCl_3_) δ 8.09–7.95 (m, 1H), 7.65–7.59 (m, 1H), 7.53–7.30 (m, 6H), 7.19–7.09 (m, 1H), 5.31–5.26 (m, 1H), 3.06–2.96 (m, 2H), 2.57–2.59 (m, 1H), 2.24–2.06 (m, 5H), 1.85–1.39 (m, 9H), 1.30–1.12 (m, 4H), 1.05–1.02 (m, 3H), 0.67 (s, 3H) ppm. ^13^C {^1^H} NMR (101 MHz, CDCl_3_) δ: 209.5, 160.6, 146.4, 144.6, 138.1, 137.7, 131.6, 130.6, 130.4, 128.8, 128.6 128.5, 127.6, 127.3, 126.6, 125.4, 63.6, 57.0, 47.8, 44.1, 39.0, 37.3, 34.9, 32.1, 32.0, 31.7, 31.6, 24.3, 23.0, 22.8, 21.9, 13.4 ppm. HRMS (MALDI-TOF) Calcd for C_34_H_38_NO [M + H]^+^ 476.2948; Found: 476.2945.

#### 1-((3a*S*,5b*R*)-11-Chloro-3a,5b-dimethyl-13-phenyl-2,3,3a,4,5,5a,5b,6,7,15,15a,15b-dodecahydro-1H-cyclopenta[5,6]naphtho[2,1-*a*]acridin-3-yl)ethan-1-one (**5hf**)

Prepared from **1h** (0.4 mmol, 126 mg) and **2f** (0.44 mmol, 102 mg) following general procedure **A** and isolated as a white solid in 73% yield (0.29 mmol, 149 mg); mp:195–196 °C; ^1^H NMR (400 MHz, CDCl_3_) δ 7.97–7.94 (m, 1H), 7.56–7.38 (m, 7H), 7.16–7.03 (m, 1H), 5.29–5.27 (m, 1H), 3.01–2.91 (m, 2H), 2.56–2.50 (m, 1H), 2.22–2.03 (m, 5H), 1.81–1.39 (m, 7H), 1.30–1.12 (m, 5H), 1.03–0.99 (m, 3H), 0.65 (s, 3H) ppm. ^13^C {^1^H} NMR (101 MHz, CDCl_3_) δ: 209.4, 160.9, 144.8, 143.8, 137.9, 137.0, 131.4, 131.3, 131.1, 130.1, 129.3, 129.0, 128.5, 128.4, 127.9, 127.6, 125.4, 63.6, 57.0, 47.8, 44.1, 39.0, 37.3, 34.8, 32.1, 31.9, 31.6, 31.5, 29.7, 24.3, 23.0, 22.9, 21.9, 13.4 ppm. HRMS (MALDI-TOF) Calcd for C_34_H_37_ClNO [M + H]^+^ 510.2558; Found: 510.2561.

#### (3a*R*,5b*R*)-11-Chloro-3a,5b-dimethyl-3-(6-methylheptan-2-yl)-2,3,3a,4,5,5a,5b,6,7,15,15a,15b-dodecahydro-1H-cyclopenta[5,6]naphtho[2,1-*a*]acridine (**5hg**)

Prepared from **1h** (0.4 mmol, 126 mg) and **2g** (0.8 mmol, 108 mg) following general procedure **B** and isolated as a white solid in 88% yield (0.35 mmol, 153 mg); ^1^H NMR (400 MHz, CDCl_3_) δ 8.11 (s, 1H), 7.92–7.90 (m, 1H), 7.75–7.74 (m, 1H), 7.57–7.54 (m, 1H), 6.33–6.31 (m, 1H), 3.27–3.09 (m, 2H), 2.62–2.56 (m, 1H), 2.39–2.21 (m, 3H), 2.17 (s, 3H), 1.95–1.52 (m, 8H), 1.39–1.13 (m, 4H), 1.00 (s, 3H), 0.71 (s, 3H) ppm. ^13^C {^1^H} NMR (101 MHz, CDCl_3_) δ: 209.4, 157.4, 139.7, 131.3, 130.7, 130.4, 129.9, 129.5, 129.48, 128.1, 126.1, 123.7, 63.6, 56.8, 49.0, 44.0, 38.8, 35.8, 34.2, 32.7, 31.6, 31.4, 29.7, 24.5, 22.9, 21.3, 18.8, 13.3 ppm. HRMS (ESI-Orbitrap) Calcd for C_28_H_33_ClNO [M + H]^+^ 434.2245; Found: 434.2246.

#### (3*R*,3a*S*,5b*R*)-3-Acetyl-3a,5b,13,14-tetramethyl-2,3,3a,4,5,5a,5b,6,7,15,15a,15b-dodecahydro-1H-cyclopenta[5,6]naphtho[2,1-*a*]acridin-3-yl Acetate (**5ja**)

Prepared from **1j** (0.4 mmol, 155 mg), **2a** (0.44 mmol, 60 mg) and TMSOTf (0.08 mmol, 15 mg) following general procedure **C** and isolated as a white solid in 63% yield (0.25 mmol, 122 mg); ^1^H NMR (400 MHz, CDCl_3_) δ 8.09 (m, 1H), 8.01 (m, 1H), 7.68 (m, 1H), 7.55 (m, 1H), 3.02 (m, 1H), 2.92–2.82 (m, 1H), 2.75 (td, *J* = 13.9, 4.9 Hz, 1H), 2.55 (s, 3H), 2.29–2.22 (m, 2H), 2.18–1.75 (m, 9H), 1.69–1.54 (m, 3H), 1.47–1.14 (m, 8H), 0.93–0.87 (m, 1H), 0.85 (s, 3H), 0.74 (s, 3H) ppm. ^13^C {^1^H} NMR (101 MHz, CDCl_3_) δ: 204.1, 170.8, 162.5, 145.3, 140.8, 133.4, 131.2, 130.8, 128.5, 127.5, 125.5, 124.2, 96.9, 52.1, 47.0, 46.8, 39.0, 37.8, 35.4, 32.5, 32.4, 31.4, 30.6, 29.7, 26.4, 24.5, 23.9, 22.1, 21.3, 21.0, 15.41, 14.5 ppm. HRMS (ESI-Orbitrap) Calcd for C_32_H_40_NO_3_ [M + H]^+^486.3003; Found: 486.3004.

#### (3a*S*,5b*R*)-3a,5b,13-Trimethyl-1,2,3a,4,5,5a,5b,6,7,15,15a,15b-dodecahydro-5′H-spiro[cyclopenta[5,6]naphtho[2,1-*a*]acridine-3,2′-furan]-5′-one (**5ka**)

Prepared from **1k** (0.4 mmol, 136 mg) **2a** (0.44 mmol, 60 mg) and TMSOTf (0.08 mmol, 15 mg) following general procedure **C** and isolated as a white solid in 96% yield (0.38 mmol, 169 mg); ^1^H NMR (400 MHz, CDCl_3_) δ 8.26–8.24 (m, 1H), 8.13–8.10 (m, 1H), 7.85–7.81 (m, 1H), 7.77–7.66 (m, 1H), 7.53–7.50 (m, 1H), 6.01–5.98 (m, 1H), 5.79–5.76 (m, 1H), 3.21 (dt, *J* = 15.8, 4.9 Hz, 1H), 3.03–2.94 (m, 1H), 2.87 (s, 3H), 2.53–2.27 (m, 2H), 2.16 (dt, *J* = 13.8, 5.5 Hz, 1H), 2.04–1.84 (m, 4H), 1.78–0.78 (m, 14H) ppm. ^13^C {^1^H} NMR (101 MHz, CDCl_3_) δ: 172.7, 159.3, 159.0, 139.4, 137.9, 132.8, 131.4, 130.6, 128.0, 127.8, 124.9, 124.4, 118.6, 98.5, 51.7, 47.2, 46.8, 37.9, 33.8, 33.3, 32.4, 31.7, 31.4, 29.7, 29.1, 23.7, 23.2, 21.4, 16.8, 15.0 ppm. HRMS (ESI-Orbitrap) Calcd for C_30_H_34_NO_2_ [M + H]^+^ 440.2584; Found: 440.2588.

#### (1*R*,13a*R*,15a*R*)-12,13a,15a-Trimethyl-1-((*R*)-6-methylheptan-2-yl)-2,3,3a,3b,4,5,13,13a,13b,14,15,15a-dodecahydro-1H-cyclopenta[5,6]naphtho[1,2-*b*]acridine (**6ba**)^29^

Prepared from **1b** (0.4 mmol, 154 mg) and **2a** (0.44 mmol, 59 mg) following general procedure **D** and isolated as a white solid in 21% yield (0.08 mmol, 41 mg); ^1^H NMR (400 MHz, CDCl_3_) δ 7.95 (d, *J* = 8.3 Hz, 1H), 7.88 (d, *J* = 8.3 Hz, 1H), 7.55 (t, *J* = 7.6 Hz, 1H), 7.40 (t, *J* = 7.6 Hz, 1H), 6.45 (d, *J* = 0.9 Hz, 1H), 3.25 (d, *J* = 15.4 Hz, 1H), 2.55 (s, 3H), 2.54–2.37 (m, 3H), 2.11–2.04 (m, 1H), 1.88–1.79 (m, 3H), 1.55–1.00 (m, 21H), 0.95 (s, 3H), 0.88 (d, *J* = 6.5 Hz, 3H), 0.87 (d, *J* = 6.5 Hz, 3H), 0.72 (s, 3H) ppm. ^13^C {^1^H} (101 MHz, CDCl_3_) δ: 156.7, 153.4, 146.5, 139.1, 129.1, 128.0, 127.5, 125.7, 125.1, 123.6, 123.4, 56.2, 56.0, 53.5, 42.4, 39.9, 39.5, 39.1, 38.8, 36.1, 36.0, 35.8, 31.7, 31.5, 28.2, 28.0, 24.3, 23.9, 22.8, 22.5, 21.8, 18.7, 18.4, 13.3, 11.9 ppm. HRMS (MALDI-TOF) Calcd for C_35_H_50_N [M + H]^+^ 484.3938; Found: 484.3939.

#### (8a*S*)-8a,14-Dimethyl-2,6b,7,8,8a,15,15a,15b-octahydro-1H-naphtho[2′,1′:4,5]indeno[1,2-*b*]quinolin-4-ol (**7ea**)

Prepared from **1e** (0.4 mmol, 108 mg) and **2a** (0.44 mmol, 60 mg) following general procedure **A** and isolated as a white solid liquid in 96% yield (0.38 mmol, 142 mg); ^1^H NMR (400 MHz, DMSO) δ 8.98 (s, 1H), 8.03–7.79 (m, 2H), 7.66–7.33 (m, 2H), 7.07–6.97 (m, 1H), 6.53–6.34 (m, 2H), 2.97–2.88 (m, 1H), 2.83–2.11 (m, 9H), 1.94–1.86 (m, 1H), 1.73–1.26 (m, 5H), 0.89 (s, 3H) ppm. ^13^C {^1^H} NMR (101 MHz, DMSO) δ: 173.0, 155.0, 146.3, 138.5, 137.1, 133.2, 130.2, 128.7, 128.0, 127.0, 125.9, 125.3, 123.8, 115.0, 112.8, 53.5, 46.1, 43.8, 37.5, 33.6, 29.1, 28.2, 27.0, 26.0, 17.8, 14.7 ppm. HRMS (MALDI-TOF) Calcd for C_26_H_28_NO [M + H]^+^ 370.2165; Found: 370.2168.

#### (6a*R*,8a*S*)-6a,8a,14-Trimethyl-3,4,5,6,6a,6b,7,8,8a,15,15a,15b-dodecahydro-1H-naphtho[2′,1′:4,5]indeno[1,2-*b*]quinolin-4-ol (**7fa**)

Prepared from **1f** (0.4 mmol, 115 mg) **2a** (0.44 mmol, 60 mg) and TMSOTf (0.08 mmol, 15 mg) following general procedure **C** and isolated as a white solid liquid in 66% yield (0.26 mmol, 102 mg); ^1^H NMR (400 MHz, CDCl_3_) δ 8.19–8.16 (m, 1H), 7.98–7.93 (m, 1H), 7.67–7.62 (m, 1H), 7.58–7.46 (m, 1H), 5.45–4.43 (m, 1H), 3.62–3.52 (m, 1H), 2.98 (dd, *J* = 14.8, 6.4 Hz, 1H), 2.63 (s, 3H), 2.37–2.07 (m, 4H), 2.00–1.49 (m, 12H), 1.13 (m, 7H) ppm. ^13^C {^1^H} NMR (101 MHz, CDCl_3_) δ: 141.4, 141.1, 133.6, 128.5, 127.4, 125.7, 123.5, 121.0, 120.9, 71.7, 55.1, 51.8, 50.5, 46.2, 42.3, 37.2, 36.8, 33.6, 31.7, 31.4, 31.0, 29.2, 20.7, 19.5, 19.4, 17.4, 15.2 ppm. HRMS (ESI-Orbitrap) Calcd for C_27_H_34_NO [M + H]^+^ 388.2635; Found: 388.2633.

#### (6a*R*,8a*S*)-6a,8a,14-Trimethyl-5,6,6a,6b,7,8,8a,15,15a,15b-decahydro-1H-naphtho[2′,1′:4,5]indeno[1,2-*b*]quinoline (**8fa**)

Prepared from **1f** (0.4 mmol, 115 mg) and **2a** (0.44 mmol, 60 mg) following general procedure **A** and isolated as a white solid liquid in 62% yield (0.25 mmol, 92 mg); ^1^H NMR (400 MHz, CDCl_3_) δ 8.11–8.09 (m, 1H), 7.94–7.92 (m, 1H), 7.64–7.58 (m, 1H), 7.49–7.45 (m, 1H), 6.01–5.95 (m, 1H), 5.71–5.59 (m, 1H), 5.49–5.42 (m, 1H), 2.99–2.91 (m, 1H), 2.60 (s, 3H), 2.55–1.62 (m, 12H), 1.29–1.17 (m, 2H), 1.11 (s, 3H), 1.08 (s, 3H) ppm. ^13^C {^1^H} NMR (101 MHz, CDCl_3_) δ: 173.5, 147.2, 141.9, 138.3, 133.3, 129.5, 128.9, 127.8, 127.5, 125.4, 125.2, 123.4, 122.3, 55.3, 48.9, 46.1, 35.5, 33.69, 33.65, 31.3, 31.0, 29.0, 23.1, 20.7, 18.9, 17.6, 15.0 ppm. HRMS (ESI-Orbitrap) Calcd for C_27_H_32_N [M + H]^+^ 370.2529; Found: 370.2528.

#### (10a*R*,12a*S*)-3,10a,12a,18-Tetramethyl-9,10,10a,10b,11,12,12a,19,19a,19b-decahydro-1H-quinolino[2″,3”:3′,4′]cyclopenta[1′,2′:5,6]naphtho[2,1-*a*]acridine (**9gaa**)

Prepared from **1g** (0.4 mmol, 114 mg) and **2a** (0.44 mmol x 2, 60 mg x 2) following general procedure **D** and isolated as a white solid in 83% yield (0.33 mmol, 163 mg); dec at 238 °C; ^1^H NMR (400 MHz, CDCl_3_) δ 8.09 (d, *J* = 8.3 Hz, 1H), 8.01 (d, *J* = 8.6 Hz, 1H), 7.98 (d, *J* = 8.5 Hz, 1H), 7.93 (d, *J* = 8.1 Hz, 1H), 7.64–7.58 (m 2H), 7.51–7.45 (m, 2H), 5.64 (d, *J* = 2.4 Hz, 1H), 2.98 (dd, *J* = 14.7, 6.4 Hz, 1H), 2.92–2.84 (m, 2H), 2.74 (s, 3H), 2.60 (s, 3H), 2.59–2.47 (m, 3H), 2.24–2.06 (m, 2H), 1.98–1.68 (m, 5H), 1.54–1.47 (m, 2H), 1.13 (s, 3H), 1.02 (s, 3H) ppm. ^13^C {^1^H} NMR (101 MHz, CDCl_3_) δ: 173.3, 161.0, 147.1, 145.6, 140.1, 139.3, 138.4, 133.1, 132.1, 129.4, 128.9, 128.2, 128.0, 127.9, 127.8, 127.4, 125.3, 125.2, 124.3, 123.4, 55.1, 47.8, 46.1, 38.2, 34.4, 33.7, 32.2, 31.4, 31.35, 28.9, 23.7, 21.6, 17.5, 15.5, 15.0 ppm. HRMS (MALDI-TOF) Calcd for C_35_H_37_N_2_ [M + H]^+^ 485.2951; Found: 485.2951.

#### (10a*R*,12a*S*)-3,10a,12a-Trimethyl-18-phenyl-9,10,10a,10b,11,12,12a,19,19a,19b-decahydro-1H-quinolino[2″,3”:3′,4′]cyclopenta[1′,2′:5,6]naphtho[2,1-*a*]acridine (**9gae**)

Prepared from **5ga** (0.4 mmol, 154 mg) and **2e** (0.44 mmol, 87 mg) following general procedure **A** and isolated as a white solid liquid in 86% yield (0.34 mmol, 188 mg); ^1^H NMR (400 MHz, CDCl_3_) δ 8.16 (d, *J* = 8.2 Hz, 1H), 8.01 (d, *J* = 8.2 Hz, 1H), 7.96 (d, *J* = 8.3 Hz, 1H), 7.67 (d, *J* = 8.3 Hz, 1H), 7.64–7.60 (m, 2H), 7.57–7.47 (m, 4H), 7.42–7.36 (m, 3H), 5.59–5.57 (m, 1H), 2.91–2.80 (m, 2H), 2.75–2.65 (m, 5H), 2.61–2.55 (m, 1H), 2.41–2.33 (m, 1H), 2.23–2.17 (m, 1H), 2.14–1.72 (m, 6H), 1.54–1.46 (m, 2H), 1.23 (s, 3H), 1.01 (s, 3H) ppm. ^13^C {^1^H} NMR (101 MHz, CDCl_3_) δ: 173.6, 160.9, 147.6, 145.5, 143.1, 140.0, 139.4, 136.7, 132.8, 132.1, 129.5, 129.1, 129.0, 128.8, 128.6, 128.3, 128.2, 128.1, 127.97, 127.95, 127.8, 126.4, 125.7, 125.40, 125.35, 125.2, 124.3, 55.6, 47.7, 46.2, 38.2, 34.4, 33.8, 32.1, 31.4, 29.8, 23.6, 21.6, 17.6, 15.5 ppm. HRMS (ESI-Orbitrap) Calcd for C_40_H_39_N_2_ [M + H]^+^ 547.3108; Found: 547.3106.

#### (3a*S*,5b*R*)-3-(4-(4-Chlorophenyl)quinolin-2-yl)-3a,5b,13-trimethyl-2,3,3a,4,5,5a,5b,6,7,15,15a,15b-dodecahydro-1H-cyclopenta[5,6]naphtho[2,1-*a*]acridine (**10haf**)

Prepared from **5ha** (0.4 mmol, 165 mg) and **2f** (0.44 mmol, 92 mg) following general procedure **A** and isolated as a white solid liquid in 44% yield (0.18 mmol, 107 mg); ^1^H NMR (400 MHz, CDCl_3_) δ 8.10–8.06 (m, 2H), 7.99 (dd, *J* = 8.4, 1.5 Hz, 1H), 7.85 (d, *J* = 2.3 Hz, 1H), 7.67–7.61 (m, 2H), 7.59–7.51 (m, 6H), 7.29–7.28 (m, 1H), 5.66 (dd, *J* = 5.1, 2.1 Hz, 1H), 3.12 (t, *J* = 9.4 Hz, 1H), 2.99–2.80 (m, 3H), 2.73 (s, 3H), 2.32 (dt, *J* = 17.8, 5.0 Hz, 1H), 2.17 (dt, *J* = 13.6, 5.1 Hz, 1H), 2.13–1.72 (m, 6H), 1.62–1.34 (m, 6H), 0.92 (s, 3H), 0.64 (s, 3H) ppm. ^13^C {^1^H} NMR (101 MHz, CDCl_3_) δ: 161.7, 161.0, 146.7, 146.6, 139.6, 137.9, 132.4, 131.5, 131.4, 129.8, 129.5, 128.8, 128.7, 128.6, 128.1, 126.2, 125.6, 124.4, 124.4, 123.1, 59.0, 57.0, 47.6, 45.6, 38.5, 38.1, 34.6, 32.6, 32.1, 31.9, 24.9, 24.7, 23.7, 21.8, 15.7, 13.4 ppm. HRMS (ESI-Orbitrap) Calcd for C_42_H_42_ClN_2_ [M + H]^+^ 609.3031; Found: 609.3026.

#### (3a*S*,5b*R*)-3a,5b-Dimethyl-13-phenyl-3-(4-phenylquinolin-2-yl)-2,3,3a,4,5,5a,5b,6,7,15,15a,15b-dodecahydro-1H-cyclopenta[5,6]naphtho[2,1-*a*]acridine (**10hee**)

Prepared from **1h** (0.31 mmol, 100 mg) and **2e** (0.68 mmol, 140 mg) following the general procedure **D** and isolated as a white solid liquid in 66% yield (0.20 mmol, 130 mg); ^1^H NMR (400 MHz, CDCl_3_) δ 8.20 (d, *J* = 8.3 Hz, 1H), 8.12 (d, *J* = 8.4 Hz, 1H), 7.90 (d, *J* = 8.2 Hz, 1H), 7.70 (t, *J* = 7.4 Hz, 1H), 7.64 (t, *J* = 7.5 Hz, 1H), 7.55–7.42 (m, 11H), 7.39–7.34 (m, 1H), 7.25 (s, 1H), 7.21–7.11 (m, 1H), 5.36 (d, *J* = 2.8 Hz, 1H), 3.17–2.99 (m, 3H), 2.84–2.75 (m, 1H), 2.20–1.22 (m, 14H), 1.04 (s, 3H), 0.62 (s, 3H) ppm. ^13^C {^1^H} NMR (101 MHz, CDCl_3_) δ: 161.3, 160.6, 148.1, 147.5, 146.0, 144.9, 138.5, 138.0, 137.7, 131.6, 131.0, 130.6, 129.6, 129.0, 128.8, 128.63, 128.57, 128.3, 128.2, 127.64, 127.60, 127.3, 126.7, 125.7, 125.6, 125.54, 125.49, 122.3, 58.9, 57.0, 48.1, 45.4, 38.5, 37.4, 34.9, 32.3, 32.0, 31.8, 24.9, 24.6, 23.0, 21.8, 13.4 ppm. HRMS (MALDI-TOF) Calcd for C_47_H_45_N_2_ [M + H]^+^ 637.3577; Found: 637.3578.

#### (3a*S*,5b*R*)-11-Chloro-3-(6-chloro-4-phenylquinolin-2-yl)-3a,5b-dimethyl-13-phenyl-2,3,3a,4,5,5a,5b,6,7,15,15a,15b-dodecahydro-1H-cyclopenta[5,6]naphtho[2,1-*a*]acridine (**10hff**)

Prepared from **1h** (0.31 mmol, 100 mg) and **2f** (0.68 mmol, 162 mg) following general procedure **D** and isolated as a white solid in 43% yield (0.14 mmol, 97 mg); mp:217–218 °C; ^1^H NMR (400 MHz, CDCl_3_) δ 8.09 (d, *J* = 9.0 Hz, 1H), 7.98 (d, *J* = 8.9 Hz, 1H), 7.85 (d, *J* = 2.3 Hz, 1H), 7.63 (dd, *J* = 9.0, 2.3 Hz, 1H), 7.60–7.41 (m, 11H), 7.24 (s, 1H), 7.18–7.10 (m, 1H), 5.36–5.33 (m, 1H), 3.13–2.96 (m, 3H), 2.86–2.70 (m, 1H), 2.17–1.20 (m, 14H), 1.03 (s, 3H), 0.59 (s, 3H) ppm. ^13^C {^1^H} NMR (101 MHz, CDCl_3_) δ: 161.7, 161.1, 146.7, 146.6, 144.8, 143.7, 138.0, 137.9, 137.1, 131.49, 131.46, 131.41, 131.36, 131.2, 130.1, 129.8, 129.5, 129.3, 129.0, 128.8 128.5, 128.4, 127.9, 127.6, 126.2, 125.4, 124.3, 123.1, 58.9, 57.0, 48.1, 45.4, 38.5, 37.4, 34.8, 32.2, 32.0, 29.7, 24.8, 24.5, 23.1, 21.7, 13.3 ppm. HRMS (MALDI-TOF) Calcd for C_47_H_43_Cl_2_N_2_ [M + H]^+^ 705.2798; Found: 705.2793.

#### Procedure for the Synthesis of Polycyclic Quinoline-Fused Steroids (**11i**)^40^

Prepared from **1h** (0.4 mmol, 126 mg) and propargylamine (0.8 mmol, 45 mg) and isolated as a white solid liquid in 67% yield (0.27 mmol, 94 mg); ^1^H NMR (400 MHz, CDCl_3_) δ 8.34–8.31 (m, 1H), 7.79–7.66 (m, 1H), 7.04–6.98 (m, 1H), 6.10–6.07 (m, 1H), 2.98–2.92 (m, 2H), 2.54–2.48 (m, 1H), 2.29–1.99 (m, 7H), 1.80–1.40 (m, 8H), 1.25–1.06 (m, 3H), 0.90 (s, 3H), 0.64–0.61 (m, 3H) ppm. ^13^C {^1^H} NMR (101 MHz, CDCl_3_) δ: 209.2, 154.6, 147.5, 139.5, 132.4, 130.4, 121.8, 121.3, 63.6, 56.8, 48.9, 44.0, 38.8, 35.5, 34.1, 32.5, 31.5, 31.3, 29.0, 24.4, 22.8, 21.2, 18.5, 13.3 ppm. HRMS (ESI-Orbitrap) Calcd for C_24_H_32_NO [M + H]^+^ 350.2478; Found: 350.2482.

#### Procedure for the Synthesis of Polycyclic Quinoline-Fused Steroids (**12ia**)

Prepared from **11i** (0.4 mmol, 140 mg) and **2a** (0.44 mmol, 60 mg) following general procedure **A** and isolated as a white solid liquid in 87% yield (0.35 mmol, 156 mg); ^1^H NMR (400 MHz, CDCl_3_) δ 8.48–8.34 (m, 1H), 8.15–8.10 (m, 1H), 7.98–7.93 (m 1H), 7.81–7.76 (m, 1H), 7.69–7.65 (m, 1H), 7.53–7.48 (m, 1H), 7.16–7.13 (m, 1H), 7.10–7.05 (m, 1H), 6.18 (m, 1H), 3.10–2.91 (m, 3H), 2.70 (s, 3H), 2.38 (s, 1H), 2.19–1.87 (m, 4H), 1.80–1.06 (m, 9H), 0.97–0.84 (m, 4H), 0.58 (s, 3H) ppm. ^13^C {^1^H} NMR (101 MHz, CDCl_3_) δ: 164.4, 161.4, 154.7, 147.4, 143.2, 139.6, 132.6, 130.7, 129.7, 128.8, 127.0, 125.4, 123.6, 122.8, 122.3, 121.4, 58.7, 56.8, 49.2, 45.3, 38.2, 35.6, 34.1, 32.7, 31.7, 29.7, 29.0, 24.9, 21.0, 18.9, 18.5, 13.2 ppm. HRMS (ESI-Orbitrap) Calcd for C_32_H_37_N_2_ [M + H]^+^ 449.2951; Found: 449.2955.

## Data Availability

The data underlying this study are available in the published article and its Supporting Information.

## Supporting Information Available

General materials and methods and ^1^H- and ^13^C NMR spectra (PDF)
