# Supplementary material for: Expanding Diversity of Fused Steroid-Quinoline Hybrids by Sequential Amination/Annulation/Aromatization Reactions
Source: J Org Chem. 2025 Mar 7;90(11):3951–63. doi: 10.1021/acs.joc.4c02981 (PMC11934142; doi:10.1021/acs.joc.4c02981)
Supplement: Supplementary file 1 — jo4c02981_si_001.pdf [file jo4c02981_si_001.pdf]

Supporting Information

Expanding Diversity of Fused Steroid-Quinoline Hybrids by Sequential Amination/Annulation/Aromatization Reactions

Caterina Momoli,[a] Antonio Arcadi,[a]* Marco Chiarini,[b] Valerio Morlacci,[a] and Laura Palombi[a]*

[a] Dipartimento di Scienze Fisiche e Chimiche, Università degli studi dell'Aquila. Via Vetoio-67100 Coppito (AQ)-Italy. E-mail: antonio.arcadi@univaq.it; laura.palombi@uinvaq.it

[b] Dipartimento di Bioscienze e Tecnologie Agroalimentari e Ambientali, Università degli studi di Teramo, Via R. Balzarini, 64110 Teramo- Italy

Table of Contents

General materials and methods **S02**

Synthetic Procedures **S03**

Characterization Data **S06**

NMR Spectra. **S15**

Computational Data **S43**

Additional references **S46**

General materials and methods

Flash chromatography was carried out using silica gel 60 (70–230 mesh, Merck, Darmstadt, Germany). Yields are usually given for isolated products showing one spot on a TLC plate and no impurities were detectable in the NMR spectrum. ^{1}H NMR spectra were recorded at 400.13 MHz on a Bruker Avance III spectrometer using the standard Bruker "zg30" sequence. Chemical shifts (in ppm) were referenced to $CDCl_3$ (δ = 7.26 ppm) or DMSO-d6 (δ =2.33 ppm) as an internal standard. ^{13}C NMR spectra were taken on the same machine at 100.613 MHz, using the standard Bruker "zgpg30" proton decupled sequence. Carbon spectra were calibrated with $CDCl_3$ (δ = 77.0 ppm) or DMSO-d6 (δ =39.5 ppm) as internal standard. Coupling constants (J) are quoted in Hertz. Mass measurements were performed using a MALDI-TOF spectrometer ABSCIEX TOF/TOF 5800, using matrix in combination with KI for the ionization, or the Thermo Fisher Orbitrap IQ-X Tribrid mass spectrometer. Unless otherwise stated, all starting materials, catalysts, and solvents were commercially available and were used as purchased. The steroidal derivative 1k and 11i are known compounds and were prepared according to the literature and identified by comparison with the NMR spectra. Reaction products were purified by flash chromatography on silica gel (60-200 μm) by elution with *n*-hexane/EtOAc mixtures.

Synthetic Procedures and Characterization Data

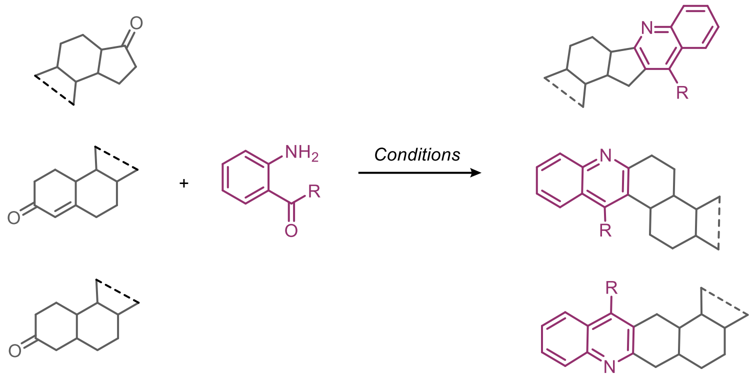

General procedure A for the synthesis of polycyclic quinoline fused steroids: 3aa, 5ba, 5ca, 5ga, 5ha, 5he, 5hf, 7ea, 8fa, 9gae, 10haf, 10hee, 10hff.

To a solution of ketosteroid **1** (0.4 mmol, 0.13M) in toluene (3 mL) was added p-toluenesulfonic acid monohydrate (0.4 mmol, 1 equiv) and 2-aminoacetophenone **2** (from 0.44 mmol to 0.80 mmol, from 1.1 eqiv to 2 equiv). After being stirred at 110 °C for between 5 to 24 hours in oil bath, the mixture was extracted with CH_2Cl_2 and a saturated solution of $NaHCO_3$ (3 x 15 mL). The crude has been loaded in a chromatographic column of silica gel eluted with a hexane/ethyl acetate mixture 9:1 to 8:2.

General procedure B for the synthesis of polycyclic quinoline fused steroids 3ab, 3ac, 5bb, 5bc, 5bd, 5hg

To a solution of ketosteroid **1** (0.4 mmol, 0.13M) in toluene (3 mL) was added p-toluenesulfonic acid monohydrate (0.4 mmol, 1 equiv) and 2-aminobenzaldehyde **2** (0.8 mmol, 2 equiv). After being stirred at 80 °C for between 5 to 24 hours in oil bath, the mixture was extracted with CH_2Cl_2 and a saturated solution of $NaHCO_3$ (3 x 15 mL). The crude has been loaded in a chromatographic column of silica gel eluted with a hexane/ethyl acetate mixture 9:1 to 8:2.

General procedure C for the synthesis of polycyclic quinoline fused steroids 5da, 5ja, 5ka,7fa

To a solution of ketosteroid **1** (0.4 mmol, 0.13M) in toluene (3 mL) was added TMSOTf (from 0.08 mmol to 0.8 mmol, from 0.20 to 2 equiv) and 2-aminoacetophenone **2** (0.44 mmol, 1.1 equiv). After being stirred at 110 °C for between 5 to 48 hours in oil bath, the mixture was extracted with CH_2Cl_2 and a H_2O (3 x 15 mL). The crude has been loaded in a chromatographic column of silica gel eluted with a hexane/ethyl acetate mixture 9:1 to 8:2.

General Procedure D one one-pot two-step for the synthesis of polycyclic quinoline fused steroids 9gaa

To a solution of ketosteroid **1g** (0.4 mmol, 0.114 g, 0.13M) in toluene (3 mL) was added *p*-toluenesulfonic acid monohydrate (0.4 mmol, 0.076 g, 1 equiv) and 2-aminoacetophenone **2a** (0.44 mmol, 0.060 g, 1.1 equiv), and the reaction mixture was stirred at 110 °C for 3 hours in oil bath, monitoring progress periodically by TLC. After complete conversion of the starting material, a second equivalent of *p*-toluenesulfonic acid monohydrate (0.4 mmol, 0.076 g, 1 equiv) and 2-aminoacetophenone **2a** (0.44 mmol, 0.060 g 1.1 equiv) were added, and the reaction was stirred at 110 °C in oil bath overnight. The mixture was then extracted with CH_2Cl_2 and a saturated $NaHCO_3$ solution (3 × 15 mL). The crude product was loaded onto a silica gel chromatographic column and eluted with a hexane/ethyl acetate mixture (9:1 to 8:2). The product **9gaa** was obtained as a white solid in 83 % yield (0.33 mmol, 0.163 g).

Procedure E for the synthesis of the polycyclic quinoline fused steroid 6ba

To a solution of ketosteroid **1b** (0.78 mmol, 0.3g, 0.39M) in toluene (2 mL) was added t-BuOK (0.78 mmol, 0.087g, 1 equiv) and 2-aminoacetophenone **2a** (0.78 mmol, 0.094 g, 1.0 equiv). After being stirred at 140 °C in oil bath for 24 h, the reaction mixture was cooled to room temperature and 3.0 mL of ethyl acetate was added and concentrated in vacuo.

The crude was loaded in a chromatographic column of silica gel and eluted with a hexane/ethyl acetate mixture 97/3. The product **6ba** was obtained as a white solid liquid in 26% yield (0.2 mmol, 98 mg).

Procedure for large scale reaction: To a solution of ketosteroid **1b** (2.6 mmol, 1.0g, 0.13M) in toluene (20 mL) was added *p*-toluenesulfonic acid monohydrate (2.6 mmol, 0.49g, 1 equiv) and 2-aminoacetophenone **2a** (2.86 mmol, 0.35g, 1.1 equiv). After being stirred at 110 °C in oil bath, the mixture was extracted with CH_2Cl_2 and a saturated solution of $NaHCO_3$ (3 x 30 mL). The crude was loaded in a chromatographic column of silica gel and eluted with a hexane/ethyl acetate mixture (from 9:1 to 8:2). The product **5ba** was obtained as a white solid in 93% yield (2.42 mmol, 1.169 g).

Procedure for the synthesis of polycyclic quinoline fused steroids 11i

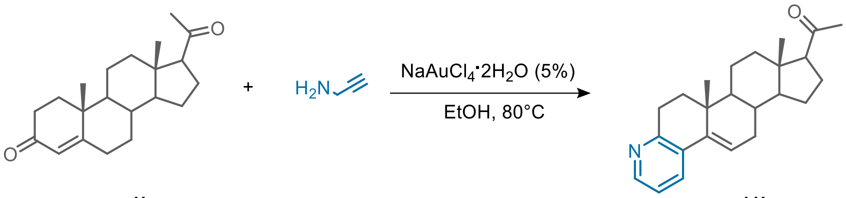

1h 11i

To a solution of ketosteroid **1h** (0.4 mmol, 0.13M) in EtOH (3 mL) was added $NaAuCl_4 \cdot 2H_2O$ (0.02 mmol, 0.05 equiv) and Propargylamine (0.8 mmol, 2 equiv). After being stirred at 80 °C for 24 hours in oil bath, the mixture has been loaded in a chromatographic column of silica gel eluted with a hexane/ethyl acetate mixture 9:1 to 8:2. The product has been obtained as a white solid liquid in 35% of yield.

Procedure for the synthesis of polycyclic quinoline fused steroids 12ia

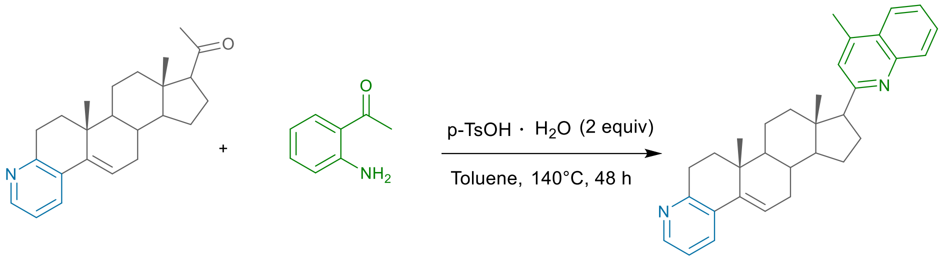

11i 2a 12ia

To a solution of **7i** (0.4 mmol, 0.13M) in toluene (3 mL) was added p-toluenesulfonic acid monohydrate (0.8 mmol, 2 equiv) and 2-benzophenone **2a** (0.44 mmol, 1.1 equiv). After being stirred at 140 °C for 48 hours in oil bath, the mixture was extracted with CH_2Cl_2 and a saturated solution of $NaHCO_3$ (3 x 15 mL). The crude has been loaded in a chromatographic column of silica gel eluted with a hexane/ethyl acetate mixture 9:1 to 8:2.

Characterization Data

(10R,13S)-10,13-dimethyl-1,6,7,8,9,10,11,12,13,14,15,16-dodecahydro-5'H-spiro[cyclopenta[a]phenanthrene-17,2'-furan]-3,5'(2H)-dione, 1k[1]

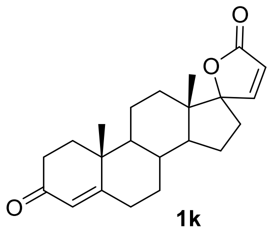

1k

Tributylamine (0.86 ml, 3.7 mmol) and $Pd(OAc)_2(PPh_3)_2$ (0.017 g, 0.022 mmol) were added to a stirred solution of ((8R,9S,10R,13S,14S,17S)-17-hydroxy-10,13-dimethyl-3-oxo-2,3,6,7,8,9,10,11,12,13,14,15,16,17-tetradecahydro-*1H*-cyclopenta[a]phenanthren-17-yl)-propynoic acid methyl ester (0.407 g, 1.1 mmol) in DMF (2 ml). The mixture was purged with nitrogen, and formic acid (0.11 ml, 2.9 mmol) was added all once. The mixture was stirred at 60 °C under nitrogen atmosphere for 6h, AcOEt and 0.1 N HCl were added, and the organic layer was separated, dried (Na_2SO_4) and concentrated at reduced pressure. The residue was purified by flash chromatography. Elution with n-hexane/EtOAc 85/15 mixture afforded (0.303g, 81% yield) as withe solid liquid, **^{1}H NMR** (400 MHz, $CDCl_3$) δ 7.45 (d, *J* = 5.7 Hz, 1H), 5.97 (d, *J* = 5.7 Hz, 1H), 5.77-5.74 (m, 1H), 2.81–2.18 (m, 5H), 2.10–1.32 (m, 12H), 1.22 (s, 3H), 1.17– 0.89 (m, 5H) ppm.

(1R,13aS,15aR)-12,13a,15a-trimethyl-1-((R)-6-methylheptan-2-yl)-2,3,3a,3b,4,5,5a,6,13,13a,13b,14,15,15a-tetradecahydro-1H-cyclopenta[5,6]naphtho[1,2-b]acridine, 3aa

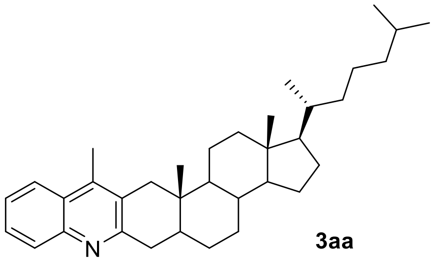

3aa

Prepared from **1a** (0.4 mmol, 155 mg) and **2a** (0.44 mmol, 59 mg) following general procedure **A** and isolated as a white solid liquid in 97% yield (0.039 mmol, 194 mg); **^{1}H NMR** (400 MHz, $CDCl_3$) δ 7.98-7.95 (m, 2H), 7.61-7.57 (m, 1H), 7.46-7.42 (m, 1H), 3.04-2.97 (m, 2H), 2.89 - 2.76 (m, 1H), 2.55 (s, 3H), 2.37-2.32 (m, 1H), 2.09-2.07 (m, 1H), 1.92 - 0.79 (m, 24H), 0.96 (d, *J* = 6.5 Hz, 3H), 0.91 (d, *J* = 6.6 Hz, 3H), 0.90 (d, *J* = 6.6 Hz, 3H), 0.81 (s, 3H), 0.72 (s, 3H) ppm. **^{13}C {^{1}H} NMR** (101 MHz, $CDCl_3$) δ 157.6, 146.0, 141.7, 129.0, 128.1, 120.0, 126.9, 125.2, 123.3, 56.4, 56.3, 54.0, 42.5, 41.4, 41.3, 40.0, 39.6, 37.9, 36.2, 35.9, 35.5, 35.0, 31.6, 28.6, 28.3, 28.1, 24.3, 23.9, 22.9, 22.6, 21.4, 18.8, 13.7, 12.06, 12.04 ppm. HRMS (ESI-Orbitrap) Calcd for $C_{35}H_{52}N$ $[M+H]^+$ 486.4094; Found: 486.4095.

(1R,13aS,15aR)-13a,15a-dimethyl-1-((R)-6-methylheptan-2-yl)-2,3,3a,3b,4,5,5a,6,13,13a,13b,14,15,15a-tetradecahydro-1H-cyclopenta[5,6]naphtho[1,2-b]acridine, 3ab[2]

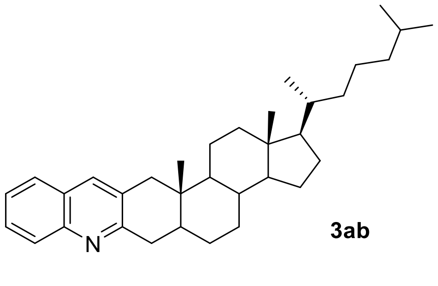

3ab

Prepared from **1a** (0.4 mmol, 155 mg) and **2b** (0.8 mmol, 97 mg) following general procedure **B** and isolated as a white solid in 98% yield (0.39 mmol, 190 mg); mp:217-218 °C; **^{1}H NMR** (400 MHz, $CDCl_3$) δ 8.18 (s, 1H), 7.78 (s, 2H), 7.61-7.49 (m, 2H), 3.12-3.01 (m, 1H), 2.99-2.95 (m, 1H), 2.84 - 2.72 (m, 1H), 2.58-2.55 (d, *J* = 16.1 Hz, 1H), 2.09-2.05 (m, 1H), 1.90–0.99 (m, 24H), 0.95 (d, *J* = 6.5 Hz, 3H), 0.89 (dd, *J* = 6.6, 1.8 Hz, 6H), 0.80 (d, *J* = 1.4 Hz, 3H), 0.71 (s, 3H) ppm. **^{13}C {^{1}H} NMR** (101 MHz, $CDCl_3$) δ 159.8, 147.1, 135.8, 130.9, 130.6, 129.1, 128.2, 125.8, 122.5, 56.4, 56.3, 53.5, 43.5, 42.5, 42.1, 40.0, 39.6, 37.4, 36.2, 35.9, 35.6, 35.2, 31.6, 28.7, 28.3, 28.1, 24.3, 23.9, 22.9, 22.7, 21.4, 18.8, 12.1, 11.7 ppm. HRMS (ESI-Orbitrap) Calcd for $C_{34}H_{50}N$ $[M+H]^+$ 472.3938; Found: 472.3938.

(1R,13aS,15aR)-9-bromo-13a,15a-dimethyl-1-((R)-6-methylheptan-2-yl)-2,3,3a,3b,4,5,5a,6,13,13a,13b,14,15,15a-tetradecahydro-1H-cyclopenta[5,6]naphtho[1,2-b]acridine, 3ac

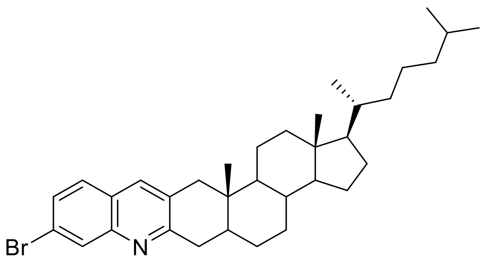

3ac

Prepared from **1a** (0.4 mmol, 155 mg) and **2c** (0.8 mmol, 160 mg) following general procedure **B** and isolated as a white solid liquid in 87% yield (0.35 mmol, 196 mg); **^{1}H NMR** (400 MHz, $CDCl_3$) δ 8.17-8.15 (m, 1H), 7.76 (s, 1H), 7.58-7.47 (m, 2H), 3.10-3.01 (m, 1H), 2.96-2.92 (m, 1H), 2.82 - 2.70 (m, 1H), 2.54-2.50 (m, 1H), 2.08-2.02 (m, 1H), 1.92 – 0.87 (m, 33H), 0.78 (s, 3H), 0.70 (s, 3H) ppm. **^{13}C {^{1}H} NMR** (101 MHz, $CDCl_3$) δ 159.7, 147.2, 135.6, 130.8, 130.6, 129.0, 128.1, 125.8, 122.4, 56.4, 56.3, 53.5, 43.5, 42.5, 42.1, 39.9, 39.5, 37.4, 36.2, 35.8, 35.5, 35.2, 31.6, 28.7, 28.3, 28.0, 24.2, 23.9, 22.9, 22.6, 21.3, 18.7, 12.0, 11.7 ppm. HRMS (ESI-Orbitrap) Calcd for $C_{34}H_{49}BrN$ $[M+H]^+$ 550.3043; Found: 550.3047.

(3aR,5bR)-3a,5b,13-trimethyl-3-((R)-6-methylheptan-2-yl)-2,3,3a,4,5,5a,5b,6,7,15,15a,15b-dodecahydro-1H-cyclopenta[5,6]naphtho[2,1-a]acridine, 5ba[3]

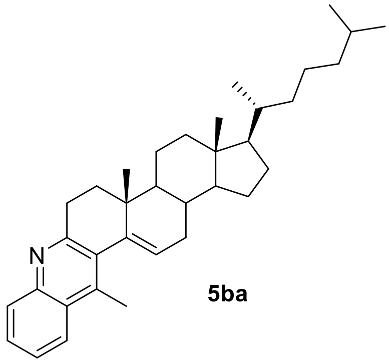

5ba

Prepared from **1b** (0.4 mmol, 154 mg) and **2a** (0.44 mmol, 60 mg) following general procedure **A** and isolated as a white solid in 98% yield (0.39 mmol, 189 mg); mp: 196–198 °C; **^{1}H NMR** (400 MHz, $CDCl_3$) δ 8.01-7.95 (m, 2H), 7.63-7.59 (m, 1H), 7.51-7.46 (m, 1H), 5.60–5.58 (m, 1H), 2.88-2.77 (m, 2H), 2.69 (s, 3H), 2.33-2.26 (m, 1H), 2.17–2.06 (m, 2H), 1.91–1.00 (m, 21H), 0.94 (d, J = 6.5 Hz, 3H), 0.91 (s, 3H), 0.88 (d, J = 6.6 Hz, 3H), 0.87 (d, J = 6.6 Hz, 3H), 0.76 (s, 3H) ppm. **^{13}C {^{1}H} NMR** (101 MHz, $CDCl_3$) δ 161.3, 145.6, 139.7, 139.2, 132.4, 128.9, 128.6, 128.2, 128.1, 125.3, 124.3, 56.8, 56.1, 47.2, 42.5, 39.9, 39.5, 37.9, 36.2, 35.8, 34.5, 32.3, 32.2, 32.0, 28.3, 28.0, 24.2, 23.9, 23.7, 22.9, 22.6, 22.0, 18.7, 15.5, 12.0 ppm. HRMS (MALDI-TOF) Calcd for $C_{35}H_{50}N$ $[M+H]^+$ 484.3938; Found: 484.3941.

(3aR,5bR)-3a,5b-dimethyl-3-(6-methylheptan-2-yl)-2,3,3a,4,5,5a,5b,6,7,15,15a,15b-dodecahydro-1H-cyclopenta[5,6]naphtho[2,1-a]acridine, 5bb

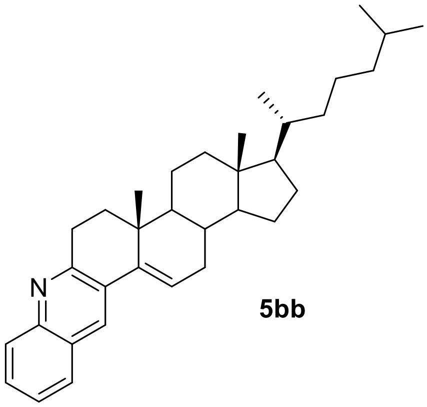

5bb

Prepared from **1b** (0.4 mmol, 154 mg) and **2b** (0.8 mmol, 97 mg) following general procedure **B** and isolated as a white solid in 55% yield (0.22 mmol, 103 mg); mp:189-192 °C; **^{1}H NMR** (400 MHz, $CDCl_3$) δ 8.21 (s, 1H), 8.00-7.96 (m, 1H), 7.78-7.74 (m, 1H), 7.64-7.58 (m, 1H), 7.46-7.42 (m, 1H), 6.32-6.30 (m, 1H), 3.30-3.10 (m, 2H), 2.35-2.21 (m, 2H), 2.11 (m, 1H), 1.94 - 1.81 (m, 2H), 1.73-1.07 (m, 19H), 1.01 (s, 3H), 0.97-0.96 (m, 3H), 0.91-0.89 (m, 6H), 0.76 (s, 3H) ppm. **^{13}C {^{1}H} NMR** (101 MHz, $CDCl_3$) δ 157.2, 147.1, 140.1, 131.2, 130.0, 128.8, 128.1, 127.52, 127.49, 125.6, 123.1, 56.7, 56.2, 49.2, 42.4, 39.8, 39.5, 36.2, 35.83, 35.82, 34.4, 32.9, 31.5, 29.9, 28.3, 28.0, 24.3, 23.9, 22.8, 22.6, 21.3, 18.82, 18.75, 12.0 ppm. HRMS (MALDI-TOF) Calcd for $C_{34}H_{48}N$ $[M+H]^+$ 470.3787; Found: 470.3782.

(3aR,5bR)-12-bromo-3a,5b-dimethyl-3-(6-methylheptan-2-yl)-2,3,3a,4,5,5a,5b,6,7,15,15a,15b-dodecahydro-1H-cyclopenta[5,6]naphtho[2,1-a]acridine, 5bc

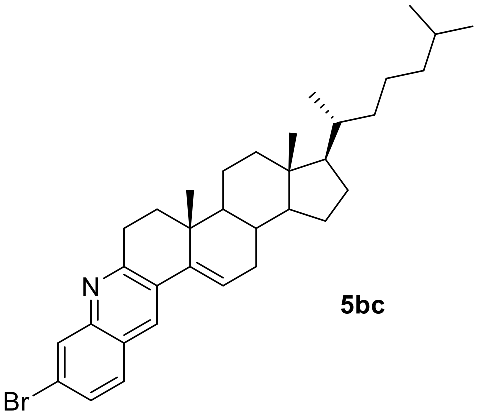

5bc

Prepared from **1b** (0.4 mmol, 154 mg) and **2c** (0.44 mmol,160 mg) following general procedure B and isolated as a white solid liquid in 93% yield (0.39 mmol, 178 mg); **^{1}H NMR** (400 MHz, $CDCl_3$) δ 8.19-8.15 (m, 2H), 7.64-7.61 (m, 1H), 7.55-7.50 (m, 1H), 6.34-6.30 (m, 1H), 3.28 -3.06 (m, 2H), 2.35 - 2.20 (m, 2H), 2.14-2.06 (m, 1H), 1.95 - 1.80 (m, 2H), 1.77-1.51 (m, 6H), 1.47-1.07 (m, 13H), 1.00 (s, 3H), 0.97 (m, 3H), 0.900 (d, *J* = 6.6 Hz, 3H), 0.896 (d, *J* = 6.6 Hz, 3H), 0.76 (s, 3H) ppm. **^{13}C {^{1}H} NMR** (101 MHz, $CDCl_3$) δ 158.4, 147.5, 139.8, 130.9, 130.5, 130.4, 129.1, 128.8, 126.1, 123.7, 122.7, 56.7, 56.2, 49.2, 42.4, 39.7, 39.5, 36.2, 35.83, 35.79, 34.2, 32.9, 31.5, 29.9, 28.3, 28.0, 24.3, 23.9, 22.9, 22.6, 21.3, 18.9, 18.7, 12.0 ppm. HRMS (MALDI-TOF) Calcd for $C_{34}H_{47}BrN$ $[M+H]^+$ 548.2886; Found: 548.2886.

(3aR,5bR)-11-bromo-3a,5b-dimethyl-3-(6-methylheptan-2-yl)-2,3,3a,4,5,5a,5b,6,7,15,15a,15b-dodecahydro-1H-cyclopenta[5,6]naphtho[2,1-a]acridine, 5bd

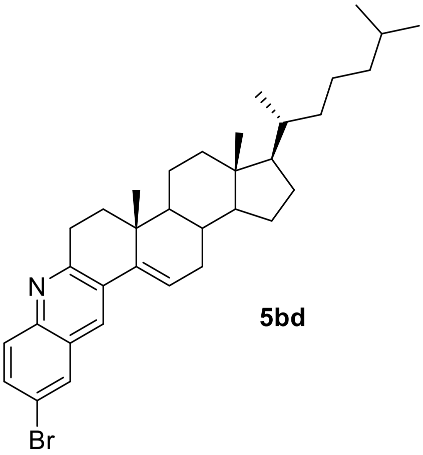

5bd

Prepared from **1b** (0.4 mmol, 154 mg) and **2e** (0.8 mmol, 160 mg) following general procedure **B** and isolated as a white solid liquid in 98% yield (0.39 mmol, 215 mg); **^{1}H NMR** (400 MHz, $CDCl_3$) δ 8.11 (s, 1H), 7.93-7.92 (m, 1H), 7.85-7.81 (m, 1H), 7.69-7.65 (m, 1H), 6.34-6.30 (m, 1H), 3.31 - 3.04 (m, 2H), 2.36 - 2.20 (m, 2H), 2.14-2.07 (m, 1H), 2.00-1.83 (m, 2H), 1.74-1.10 (m, 19H), 1.01 (s, 3H), 0.98-0.95 (m, 3H), 0.91-0.88 (m, 6H), 0.76 (s, 3H) ppm. **^{13}C {^{1}H} NMR** (101 MHz, $CDCl_3$) δ 157.9, 146.0, 139.8, 132.1, 130.9, 130.0, 129.9, 129.4, 128.7, 124.1, 119.2, 56.7, 56.2, 49.2, 42.4, 39.7, 39.5, 36.2, 35.8 (2C), 34.2, 32.9, 31.5, 29.9, 28.3, 28.0, 24.3, 23.9, 22.8, 22.6, 21.3, 18.9, 18.7, 12.0 ppm. HRMS (MALDI-TOF) Calcd for $C_{34}H_{47}BrN$ $[M+H]^+$ 548.2886; Found: 548.2884.

(3S,3aS,5bR)-3a,5b,13-trimethyl-2,3,3a,4,5,5a,5b,6,7,15,15a,15b-dodecahydro-1H-cyclopenta[5,6]naphtho[2,1-a]acridin-3-ol, 5ca

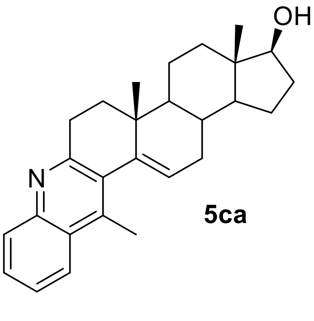

5ca

Prepared from **1c** (0.4 mmol, 115 mg) and **2a** (0.44 mmol, 60 mg) following general procedure **A** and isolated as a white solid liquid in 94% yield (0.38 mmol, 146 mg); **^{1}H NMR** (400 MHz, $CDCl_3$) δ 8.11-7.88 (m, 2H), 7.66-7.60 (m, 1H), 7.53-7.57 (m, 1H), 5.61-5.58 (m, 1H), 3.73 (t, *J* = 8.5 Hz, 1H), 2.95-2.78 (m, 2H), 2.71 (s, 3H), 2.37-2.27 (m, 2H), 2.23-2.07 (m, 2H), 1.94-1.88 (m, 1H), 1.85-1.02 (m, 11H), 0.94 (s, 3H), 0.86 (s, 3H) ppm. **^{13}C {^{1}H} NMR** (101 MHz, $CDCl_3$) δ 161.2, 145.7, 139.8, 139.3, 132.3, 129.0, 128.24, 128.23, 128.1, 125.4, 124.3, 81.6, 51.5, 47.5, 43.0, 38.0, 36.8, 34.6, 32.3, 32.2, 31.6, 30.6, 23.7, 23.4, 21.7, 15.5, 11.3 ppm. HRMS (MALDI-TOF) Calcd for $C_{27}H_{34}NO$ $[M+H]^+$ 388.2635; Found: 388.2635.

3-ethynyl-3a,5b,13-trimethyl-2,3,3a,4,5,5a,5b,6,7,15,15a,15b-dodecahydro-1H-cyclopenta[5,6]naphtho[2,1-a]acridin-3-ol, 5da

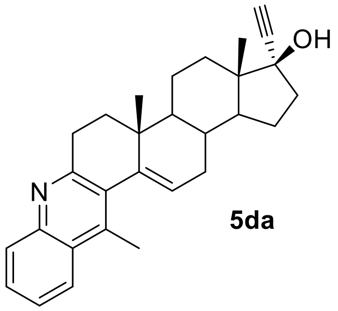

5da

Prepared from **1d** (0.4 mmol, 125 mg), **2a** (0.44 mmol, 60 mg) and TMSOTf (0.8 mmol, 146 mg) following general procedure **C** and isolated as a white solid in 67% yield (0.27mmol, 110 mg); mp:184-187 °C; **^{1}H NMR** (400 MHz, $CDCl_3$) δ 8.03 - 7.96 (m, 2H), 7.65-7.61 (m, 1H), 7.52-7.48 (m, 1H), 5.61-5.59 (m, 1H), 2.89-2.79 (m, 2H), 2.70 (s, 3H), 2.60 (s, 1H), 2.38–1.30 (m, 2H), 2.17 (dt, *J* = 13.5, 5.0 Hz, 1H), 2.09-2.02 (m, 1H), 1.87-1.25 (m, 12H), 0.95 (s, 3H), 0.93 (s, 3H) ppm. **^{13}C {^{1}H} NMR** (101 MHz, $CDCl_3$) δ 161.1, 145.4, 139.7, 139.4, 132.2, 128.8, 128.3, 128.1, 128.0, 125.4, 124.3, 87.5, 79.7, 74.0, 50.8, 47.0, 46.8, 39.0, 37.9, 34.5, 32.8, 32.7, 32.1, 31.5, 23.7, 23.1, 21.7, 15.5, 12.8 ppm. HRMS (MALDI-TOF) Calcd for $C_{29}H_{34}NO$ $[M+H]^+$ 412.2635; Found: 412.2637.

(3aS,5bR)-3a,5b,13-trimethyl-1,2,3a,4,5,5a,5b,6,7,15,15a,15b-dodecahydro-3H-cyclopenta[5,6]naphtho[2,1-a]acridin-3-one, 5ga

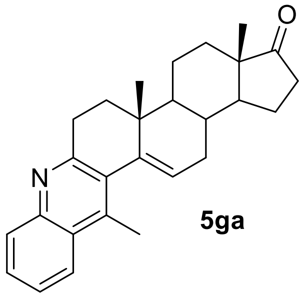

5ga

Prepared from **1g** (0.4 mmol, 115 mg) and **2a** (0.44 mmol, 60 mg) following general procedure **A** and isolated as a white solid in 84% yield (0.34 mmol, 133 mg); dec. at 207 °C; **^{1}H NMR** (400 MHz, $CDCl_3$) δ 7.98-7.93 (m, 2H), 7.62-7.58 (m, 1H), 7.49-7.45 (m, 1H), 5.59-5.58 (m, 1H), 2.87-2.75 (m, 2H), 2.67 (s, 3H), 2.57 - 2.37 (m, 3H), 2.15 - 2.06 (m, 2H), 2.00-1.78 (m, 5H), 1.64-1.31 (m, 5H), 0.92-0.91 (m, 6H) ppm. **^{13}C {^{1}H} NMR** (101 MHz, $CDCl_3$) δ 220.8, 161.0, 145.7, 140.0, 139.3, 132.0, 129.0, 128.4, 128.0, 127.7, 125.4, 124.4, 51.8, 47.8, 47.4, 38.1, 35.9, 34.5, 32.2, 31.8, 31.6, 30.8, 23.7, 21.8, 21.4, 15.5, 13.7 ppm. HRMS (MALDI-TOF) Calcd for $C_{27}H_{32}NO$ $[M+H]^+$ 386.2478; Found: 386.2477.

1-((3aS,5bR)-3a,5b,13-trimethyl-2,3,3a,4,5,5a,5b,6,7,15,15a,15b-dodecahydro-1H-cyclopenta[5,6]naphtho[2,1-a]acridin-3-yl)ethan-1-one, 5ha

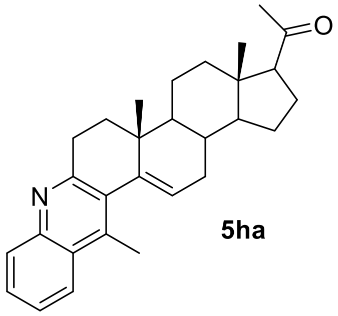

5ha

Prepared from **1h** (0.4 mmol, 126 mg) and **2a** (0.44 mmol, 60 mg) following general procedure **A** and isolated as a white solid in 98% yield (0.39 mmol, 162 mg); dec. at 205 °C; **^{1}H NMR** (400 MHz, $CDCl_3$) δ 7.97 - 7.91 (m, 2H), 7.61-7.57 (m, 1H), 7.47-7.44 (m, 1H), 5.55-5.54 (m, 1H), 2.83-2.79 (m, 2H), 2.65 (s, 3H), 2.54-2.50 (m, 1H), 2.30-2.05 (m, 7H), 1.75-1.18 (m, 11H), 0.87 (s, 3H), 0.65 (s, 3H) ppm. **^{13}C {^{1}H} NMR** (101 MHz, $CDCl_3$) δ 209.3, 160.9, 145.5, 139.5, 139.1, 132.0, 128.8, 128.11, 128.06, 127.9, 125.2, 124.2, 63.4, 56.8, 47.0, 44.0, 38.8, 37.8, 34.4, 32.1, 32.0, 31.6, 31.5, 24.2, 23.5, 22.7, 21.9, 15.4, 13.3 ppm. HRMS (MALDI-TOF) Calcd for $C_{29}H_{36}NO$ $[M+H]^+$ 414.2791; Found: 414.2794.

1-((3aS,5bR)-3a,5b-dimethyl-13-phenyl-2,3,3a,4,5,5a,5b,6,7,15,15a,15b-dodecahydro-1H-cyclopenta[5,6]naphtho[2,1-a]acridin-3-yl)ethan-1-one, 5he

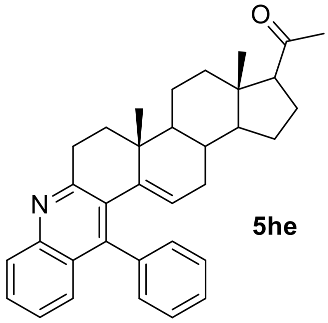

5he

Prepared from **1h** (0.4 mmol, 126 mg) and **2e** (0.44 mmol, 87 mg) following general procedure **A** and isolated as a white solid in 63% yield (0.25 mmol, 120 mg); mp:197-199 °C; **^{1}H NMR** (400 MHz, $CDCl_3$) δ 8.09-7.95 (m, 1H), 7.65-7.59 (m, 1H), 7.53-7.30 (m, 6H), 7.19-7.09 (m, 1H), 5.31-5.26 (m, 1H), 3.06-2.96 (m, 2H), 2.57-2.59 (m, 1H), 2.24-2.06 (m, 5H), 1.85-1.39 (m, 9H), 1.30-1.12 (m, 4H), 1.05-1.02 (m, 3H), 0.67 (s, 3H) ppm. **^{13}C {^{1}H} NMR** (101 MHz, $CDCl_3$) δ 209.5, 160.6, 146.4, 144.6, 138.1, 137.7, 131.6, 130.6, 130.4, 128.8, 128.6 128.5, 127.6, 127.3, 126.6, 125.4, 63.6, 57.0, 47.8, 44.1, 39.0, 37.3, 34.9, 32.1, 32.0, 31.7, 31.6, 24.3, 23.0, 22.8, 21.9, 13.4 ppm. HRMS (MALDI-TOF) Calcd for $C_{34}H_{38}NO$ $[M+H]^+$ 476.2948; Found: 476.2945.

1-((3aS,5bR)-11-chloro-3a,5b-dimethyl-13-phenyl-2,3,3a,4,5,5a,5b,6,7,15,15a,15b-dodecahydro-1H-cyclopenta[5,6]naphtho[2,1-a]acridin-3-yl)ethan-1-one, 5hf

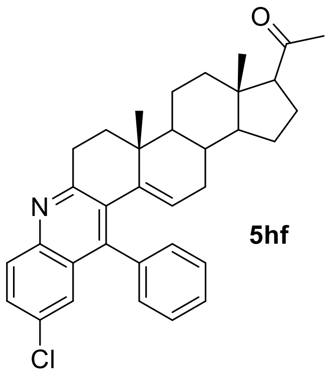

5hf

Prepared from **1h** (0.4 mmol, 126 mg) and **2f** (0.44 mmol, 102 mg) following general procedure **A** and isolated as a white solid in 73% yield (0.29 mmol, 149 mg); mp:195-196 °C; **^{1}H NMR** (400 MHz, $CDCl_3$) δ 7.97-7.94 (m, 1H), 7.56 - 7.38 (m, 7H), 7.16 - 7.03 (m, 1H), 5.29-5.27 (m, 1H), 3.01-2.91 (m, 2H), 2.56-2.50 (m, 1H), 2.22-2.03 (m, 5H), 1.81-1.39 (m, 7H), 1.30-1.12 (m, 5H), 1.03-0.99 (m, 3H), 0.65 (s, 3H) ppm. **^{13}C {^{1}H} NMR** (101 MHz, $CDCl_3$) δ 209.4, 160.9, 144.8, 143.8, 137.9, 137.0, 131.4, 131.3, 131.1, 130.1, 129.3, 129.0, 128.5, 128.4, 127.9, 127.6, 125.4, 63.6, 57.0, 47.8, 44.1, 39.0, 37.3, 34.8, 32.1, 31.9, 31.6, 31.5, 29.7, 24.3, 23.0, 22.9, 21.9, 13.4 ppm. HRMS (MALDI-TOF) Calcd for $C_{34}H_{37}ClNO$ $[M+H]^+$ 510.2558; Found: 510.2561.

(3aR,5bR)-11-chloro-3a,5b-dimethyl-3-(6-methylheptan-2-yl)-2,3,3a,4,5,5a,5b,6,7,15,15a,15b-dodecahydro-1H-cyclopenta[5,6]naphtho[2,1-a]acridine, 5hg

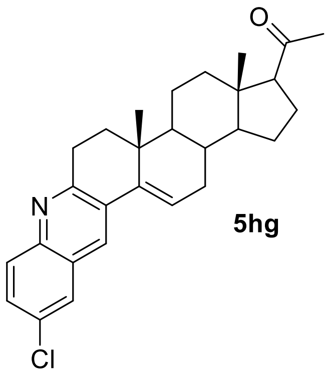

5hg

Prepared from **1h** (0.4 mmol, 126 mg) and **2g** (0.8 mmol, 108 mg) following general procedure **B** and isolated as a white solid liquid in 88% yield (0.35 mmol, 153 mg); **^{1}H NMR** (400 MHz, $CDCl_3$) δ 8.11 (s, 1H), 7.92-7.90 (m, 1H), 7.75-7.74 (m, 1H), 7.57-7.54 (m, 1H), 6.33-6.31 (m, 1H), 3.27 - 3.09 (m, 2H), 2.62-2.56 (m, 1H), 2.39-2.21 (m, 3H), 2.17 (s, 3H), 1.95-1.52 (m, 8H), 1.39-1.13 (m, 4H), 1.00 (s, 3H), 0.71 (s, 3H) ppm. **^{13}C {^{1}H} NMR** (101 MHz, $CDCl_3$) δ 209.4, 157.4, 139.7, 131.3, 130.7, 130.4, 129.9, 129.53, 129.48, 128.1, 126.1, 123.7, 63.6, 56.8, 49.0, 44.0, 38.8, 35.8, 34.2, 32.7, 31.6, 31.4, 29.7, 24.5, 22.9, 21.3, 18.8, 13.3 ppm. HRMS (ESI-Orbitrap) Calcd for $C_{28}H_{33}ClNO$ $[M+H]^+$ 434.2245; Found: 434.2246.

(3R,3aS,5bR)-3-acetyl-3a,5b,13,14-tetramethyl-2,3,3a,4,5,5a,5b,6,7,15,15a,15b-dodecahydro-1H-cyclopenta[5,6]naphtho[2,1-a]acridin-3-yl acetate, 5ja

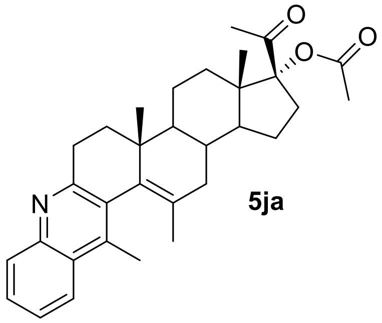

5ja

Prepared from **1j** (0.4 mmol, 155 mg), **2a** (0.44 mmol, 60 mg) and TMSOTf (0.08 mmol, 15 mg) following general procedure **C** and isolated as a white solid liquid in 63% yield (0.25 mmol, 122 mg); **^{1}H NMR** (400 MHz, $CDCl_3$) δ 8.09 (m, 1H), 8.01 (m, 1H), 7.68 (m, 1H), 7.55 (m, 1H), 3.02 (m, 1H), 2.92–2.82 (m, 1H), 2.75 (td, J = 13.9, 4.9 Hz, 1H), 2.55 (s, 3H), 2.29-2.22 (m, 2H), 2.18-1.75 (m, 9H), 1.69-1.54 (m, 3H), 1.47-1.14 (m, 8H), 0.93-0.87 (m, 1H), 0.85 (s, 3H), 0.74 (s, 3H) ppm. **^{13}C {^{1}H} NMR** (101 MHz, $CDCl_3$) δ 204.1, 170.8, 162.5, 145.3, 140.8, 133.4, 131.2, 130.8, 128.5, 127.5, 125.5, 124.2, 96.9, 52.1, 47.0, 46.8, 39.0, 37.8, 35.4, 32.5, 32.4, 31.4, 30.6, 29.7, 26.4, 24.5, 23.9, 22.1, 21.3, 21.0, 15.41, 14.5 ppm. HRMS (ESI-Orbitrap) Calcd for $C_{32}H_{40}NO_3$ $[M+H]^+$ 486.3003; Found: 486.3004.

(3aS,5bR)-3a,5b,13-trimethyl-1,2,3a,4,5,5a,5b,6,7,15,15a,15b-dodecahydro-5'H-spiro[cyclopenta[5,6]naphtho[2,1-a]acridine-3,2'-furan]-5'-one, 5ka

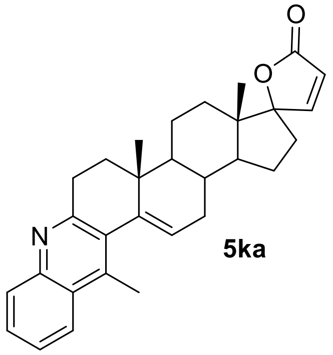

5ka

Prepared from **1k** (0.4 mmol, 136 mg) **2a** (0.44 mmol, 60 mg) and TMSOTf (0.08 mmol, 15 mg) following general procedure **C** and isolated as a white solid liquid in 96% yield (0.38 mmol, 169 mg); **^{1}H NMR** (400 MHz, $CDCl_3$) δ 8.26-8.24 (m, 1H), 8.13-8.10 (m, 1H), 7.85-7.81 (m, 1H), 7.77-7.66 (m, 1H), 7.53-7.50 (m, 1H), 6.01-5.98 (m, 1H), 5.79-5.76 (m, 1H), 3.21 (dt, *J* = 15.8, 4.9 Hz, 1H), 3.03-2.94 (m, 1H), 2.87 (s, 3H), 2.53–2.27 (m, 2H), 2.16 (dt, *J* = 13.8, 5.5 Hz, 1H), 2.04–1.84 (m, 4H), 1.78–0.78 (m, 14H) ppm. **^{13}C {^{1}H} NMR** (101 MHz, $CDCl_3$) δ 172.7, 159.3, 159.0, 139.4, 137.9, 132.8, 131.4, 130.6, 128.0, 127.8, 124.9, 124.4, 118.6, 98.5, 51.7, 47.2, 46.8, 37.9, 33.8, 33.3, 32.4, 31.7, 31.4, 29.7, 29.1, 23.7, 23.2, 21.4, 16.8, 15.0 ppm. HRMS (ESI-Orbitrap) Calcd for $C_{30}H_{34}NO_2$ $[M+H]^+$ 440.2584; Found: 440.2588.

(1R,13aR,15aR)-12,13a,15a-trimethyl-1-((R)-6-methylheptan-2-yl)-2,3,3a,3b,4,5,13,13a,13b,14,15,15a-dodecahydro-1H-cyclopenta[5,6]naphtho[1,2-b]acridine, 6ba[3]

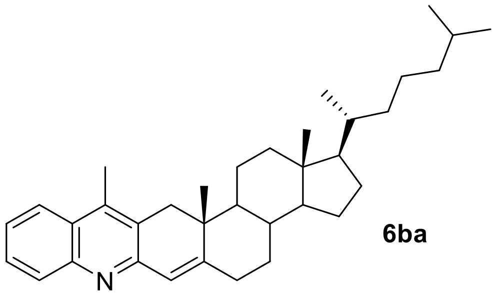

6ba

Prepared from **1b** (0.4 mmol, 154 mg) and **2a** (0.44 mmol, 59 mg) following general procedure **D** and isolated as a white solid liquid in 21% yield (0.08 mmol, 41 mg); **^{1}H NMR** (400 MHz, $CDCl_3$) δ 7.95 (d, *J* = 8.3 Hz, 1H), 7.88 (d, *J* = 8.3 Hz, 1H), 7.55 (t, *J* = 7.6 Hz, 1H), 7.40 (t, *J* = 7.6 Hz, 1H), 6.45 (d, *J* = 0.9 Hz, 1H), 3.25 (d, *J* = 15.4 Hz, 1H), 2.55 (s, 3H), 2.54 – 2.37 (m, 3H), 2.11-2.04 (m, 1H), 1.88 – 1.79 (m, 3H), 1.55-1.00 (m, 21H), 0.95 (s, 3H), 0.88 (d, *J* = 6.5 Hz, 3H), 0.87 (d, *J* = 6.5 Hz, 3H), 0.72 (s, 3H) ppm. **^{13}C {^{1}H}** (101 MHz, $CDCl_3$) δ 156.7, 153.4, 146.5, 139.1, 129.1, 128.0, 127.5, 125.7, 125.1, 123.6, 123.4, 56.2, 56.0, 53.5, 42.4, 39.9, 39.5, 39.1, 38.8, 36.1, 36.0, 35.8, 31.7, 31.5, 28.2, 28.0, 24.3, 23.9, 22.8, 22.5, 21.8, 18.7, 18.4, 13.3, 11.9 ppm. HRMS (MALDI-TOF) Calcd for $C_{35}H_{50}N$ $[M+H]^+$ 484.3938; Found: 484.3939.

(8aS)-8a,14-dimethyl-2,6b,7,8,8a,15,15a,15b-octahydro-1H-naphtho[2',1':4,5]indeno[1,2-b]quinolin-4-ol, 7ea

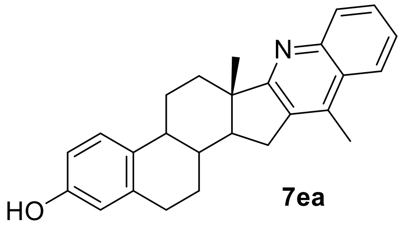

7ea

Prepared from **1e** (0.4 mmol, 108 mg) and **2a** (0.44 mmol, 60 mg) following general procedure **A** and isolated as a white solid liquid in 96% yield (0.38 mmol, 142 mg); **^{1}H NMR** (400 MHz, DMSO) δ 8.98 (s, 1H), 8.03-7.79 (m, 2H), 7.66-7.33 (m, 2H), 7.07-6.97 (m, 1H), 6.53-6.34 (m, 2H), 2.97-2.88 (m, 1H), 2.83-2.11 (m, 9H), 1.94-1.86 (m, 1H), 1.73–1.26 (m, 5H), 0.89 (s, 3H) ppm. **^{13}C {^{1}H} NMR** (101 MHz, DMSO) δ 173.0, 155.0, 146.3, 138.5, 137.1, 133.2, 130.2, 128.7, 128.0, 127.0, 125.9, 125.3, 123.8, 115.0, 112.8, 53.5, 46.1, 43.8, 37.5, 33.6, 29.1, 28.2, 27.0, 26.0, 17.8, 14.7 ppm. HRMS (MALDI-TOF) Calcd for $C_{26}H_{28}NO$ $[M+H]^+$ 370.2165; Found: 370.2168.

(6aR,8aS)-6a,8a,14-trimethyl-3,4,5,6,6a,6b,7,8,8a,15,15a,15b-dodecahydro-1H-naphtho[2',1':4,5]indeno[1,2-b]quinolin-4-ol,-7fa

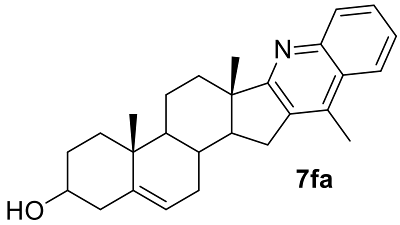

7fa

Prepared from **1f** (0.4 mmol, 115 mg) **2a** (0.44 mmol, 60 mg) and TMSOTf (0.08 mmol, 15 mg) following general procedure **C** and isolated as a white solid liquid in 66% yield (0.26 mmol, 102 mg); **^{1}H NMR** (400 MHz, $CDCl_3$) δ 8.19-8.16 (m, 1H), 7.98-7.93 (m, 1H), 7.67-7.62 (m, 1H), 7.58–7.46 (m, 1H), 5.45-4.43 (m, 1H), 3.62-3.52 (m, 1H), 2.98 (dd, *J* = 14.8, 6.4 Hz, 1H), 2.63 (s, 3H), 2.37-2.07 (m, 4H), 2.00-1.49 (m, 12H), 1.13 (m, 7H) ppm. **^{13}C {^{1}H} NMR** (101 MHz, $CDCl_3$) δ 141.4, 141.1, 133.6, 128.5, 127.4, 125.7, 123.5, 121.0, 120.9, 71.7, 55.1, 51.8, 50.5, 46.2, 42.3, 37.2, 36.8, 33.6, 31.7, 31.4, 31.0, 29.2, 20.7, 19.5, 19.4, 17.4, 15.2 ppm. HRMS (ESI-Orbitrap) Calcd for $C_{27}H_{34}NO$ $[M+H]^+$ 388.2635; Found: 388.2633.

(6aR,8aS)-6a,8a,14-trimethyl-5,6,6a,6b,7,8,8a,15,15a,15b-decahydro-1H-naphtho[2',1':4,5]indeno[1,2-b]quinoline, 8fa

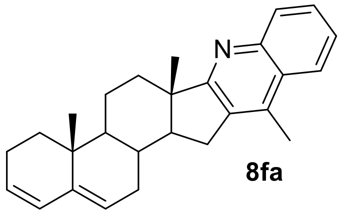

8fa

Prepared from **1f** (0.4 mmol, 115 mg) and **2a** (0.44 mmol, 60 mg) following general procedure **A** and isolated as a white solid liquid in 62% yield (0.25 mmol, 92 mg); **^{1}H NMR** (400 MHz, $CDCl_3$) δ 8.11-8.09 (m, 1H), 7.94-7.92 (m, 1H), 7.64-7.58 (m, 1H), 7.49-7.45 (m, 1H), 6.01-5.95 (m, 1H), 5.71–5.59 (m, 1H), 5.49-5.42 (m, 1H), 2.99-2.91 (m, 1H), 2.60 (s, 3H), 2.55 – 1.62 (m, 12H), 1.29-1.17 (m, 2H), 1.11 (s, 3H), 1.08 (s, 3H) ppm. **^{13}C {^{1}H} NMR** (101 MHz, $CDCl_3$) δ 173.5, 147.2, 141.9, 138.3, 133.3, 129.5, 128.9, 127.8, 127.5, 125.4, 125.2, 123.4, 122.3, 55.3, 48.9, 46.1, 35.5, 33.69, 33.65, 31.3, 31.0, 29.0, 23.1, 20.7, 18.9, 17.6, 15.0 ppm. HRMS (ESI-Orbitrap) Calcd for $C_{27}H_{32}N$ $[M+H]^+$ 370.2529; Found: 370.2528.

(10aR,12aS)-3,10a,12a,18-tetramethyl-9,10,10a,10b,11,12,12a,19,19a,19b-decahydro-1H-quinolino[2'',3'':3',4']cyclopenta[1',2':5,6]naphtho[2,1-a]acridine, 9gaa

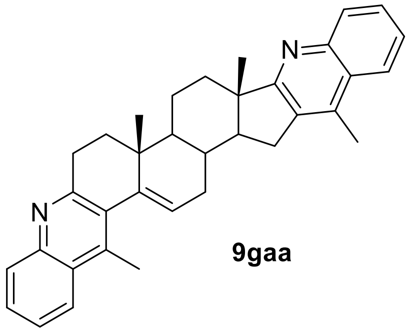

9gaa

Prepared from **1g** (0.4 mmol, 114 mg) and **2a** (0.44 mmol x 2, 60 mg x 2) following general procedure **D** and isolated as a white solid in 83% yield (0.33 mmol, 163 mg); dec. at 238 °C; **^{1}H NMR** (400 MHz, $CDCl_3$) δ 8.09 (d, *J* = 8.3 Hz, 1H), 8.01 (d, *J* = 8.6 Hz, 1H), 7.98 (d, *J* = 8.5 Hz, 1H), 7.93 (d, *J* = 8.1 Hz, 1H), 7.64-7.58 (m 2H), 7.51-7.45 (m, 2H), 5.64 (d, *J* = 2.4 Hz, 1H), 2.98 (dd, *J* = 14.7, 6.4 Hz, 1H), 2.92-2.84 (m, 2H), 2.74 (s, 3H), 2.60 (s, 3H), 2.59-2.47 (m, 3H), 2.24-2.06 (m, 2H), 1.98-1.68 (m, 5H), 1.54-1.47 (m, 2H), 1.13 (s, 3H), 1.02 (s, 3H) ppm. **^{13}C {^{1}H} NMR** (101 MHz, $CDCl_3$) δ 173.3, 161.0, 147.1, 145.6, 140.1, 139.3, 138.4, 133.1, 132.1, 129.4, 128.9, 128.2, 128.0, 127.9, 127.8, 127.4, 125.3, 125.2, 124.3, 123.4, 55.1, 47.8, 46.1, 38.2, 34.4, 33.7, 32.2, 31.40, 31.35, 28.9, 23.7, 21.6, 17.5, 15.5, 15.0 ppm. HRMS (MALDI-TOF) Calcd for $C_{35}H_{37}N_2$ $[M+H]^+$ 485.2951; Found: 485.2951.

(10aR,12aS)-3,10a,12a-trimethyl-18-phenyl-9,10,10a,10b,11,12,12a,19,19a,19b-decahydro-1H-quinolino[2'',3'':3',4']cyclopenta[1',2':5,6]naphtho[2,1-a]acridine, 9gae

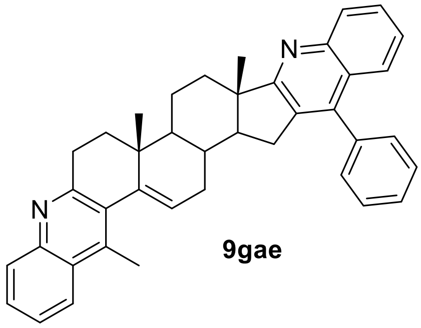

9gae

Prepared from **5ga** (0.4 mmol, 154 mg) and **2e** (0.44 mmol, 87 mg) following general procedure **A** and isolated as a white solid liquid in 86% yield (0.34 mmol, 188 mg); **^{1}H NMR** (400 MHz, $CDCl_3$) δ 8.16 (d, *J* = 8.2 Hz, 1H), 8.01 (d, *J* = 8.2 Hz, 1H), 7.96 (d, *J* = 8.3 Hz, 1H), 7.67 (d, *J* = 8.3 Hz, 1H), 7.64-7.60 (m, 2H), 7.57-7.47 (m, 4H), 7.42-7.36 (m, 3H), 5.59-5.57 (m, 1H), 2.91-2.80 (m, 2H), 2.75-2.65 (m, 5H), 2.61-2.55 (m, 1H), 2.41-2.33 (m, 1H), 2.23-2.17 (m, 1H), 2.14-1.72 (m, 6H), 1.54-1.46 (m, 2H), 1.23 (s, 3H), 1.01 (s, 3H) ppm. **^{13}C {^{1}H} NMR** (101 MHz, $CDCl_3$) δ 173.6, 160.9, 147.6, 145.5, 143.1, 140.0, 139.4, 136.7, 132.8, 132.1, 129.5, 129.1, 129.0, 128.8, 128.6, 128.3, 128.2, 128.1, 127.97, 127.95, 127.8, 126.4, 125.7, 125.40, 125.35, 125.2, 124.3, 55.6, 47.7, 46.2, 38.2, 34.4, 33.8, 32.1, 31.4, 29.8, 23.6, 21.6, 17.6, 15.5 ppm. HRMS (ESI-Orbitrap) Calcd for $C_{40}H_{39}N_2$ $[M+H]^+$ 547.3108; Found: 547.3106.

(3aS,5bR)-3-(4-(4-chlorophenyl)quinolin-2-yl)-3a,5b,13-trimethyl-2,3,3a,4,5,5a,5b,6,7,15,15a,15b-dodecahydro-1H-cyclopenta[5,6]naphtho[2,1-a]acridine, 10haf

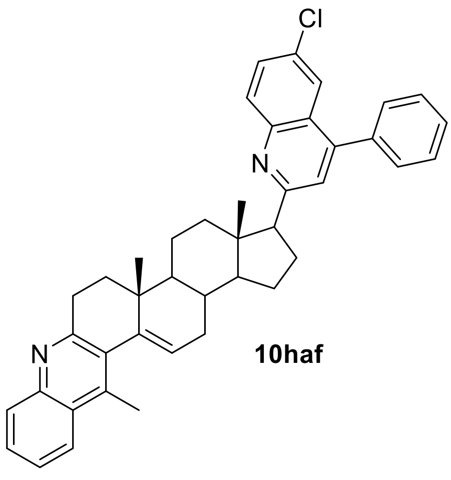

10haf

Prepared from **5ha** (0.4 mmol, 165 mg) and **2f** (0.44 mmol, 92 mg) following general procedure **A** and isolated as a white solid liquid in 44% yield (0.18 mmol, 107 mg); **^{1}H NMR** (400 MHz, $CDCl_3$) δ 8.10-8.06 (m, 2H), 7.99 (dd, *J* = 8.4, 1.5 Hz, 1H), 7.85 (d, *J* = 2.3 Hz, 1H), 7.67 - 7.61 (m, 2H), 7.59-7.51 (m, 6H), 7.29-7.28 (m, 1H), 5.66 (dd, *J* = 5.1, 2.1 Hz, 1H), 3.12 (t, *J* = 9.4 Hz, 1H), 2.99 - 2.80 (m, 3H), 2.73 (s, 3H), 2.32 (dt, *J* = 17.8, 5.0 Hz, 1H), 2.17 (dt, *J* = 13.6, 5.1 Hz, 1H), 2.13-1.72 (m, 6H), 1.62-1.34 (m, 6H), 0.92 (s, 3H), 0.64 (s, 3H) ppm. **^{13}C {^{1}H} NMR** (101 MHz, $CDCl_3$) δ 161.7, 161.0, 146.7, 146.6, 139.6, 137.9, 132.4, 131.5, 131.4, 129.8, 129.5, 128.8, 128.7, 128.6, 128.1, 126.2, 125.6, 124.4, 124.4, 123.1, 59.0, 57.0, 47.6, 45.6, 38.5, 38.1, 34.6, 32.6, 32.1, 31.9, 24.9, 24.7, 23.7, 21.8, 15.7, 13.4 ppm. HRMS (ESI-Orbitrap) Calcd for $C_{42}H_{42}ClN_2$ $[M+H]^+$ 609.3031; Found: 609.3026.

(3aS,5bR)-3a,5b-dimethyl-13-phenyl-3-(4-phenylquinolin-2-yl)-2,3,3a,4,5,5a,5b,6,7,15,15a,15b-dodecahydro-1H-cyclopenta[5,6]naphtho[2,1-a]acridine, 10hee

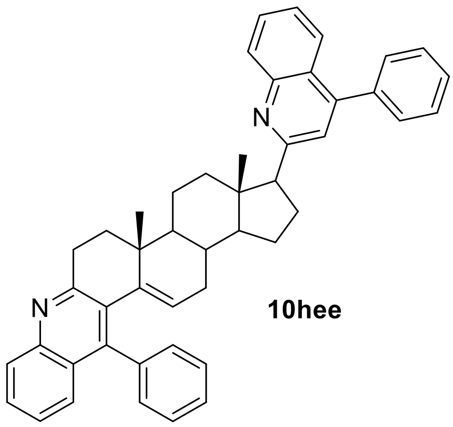

10hee

Prepared from **1h** (0.31mmol, 100 mg) and **2e** (0.68mmol, 140 mg) following the general procedure D and isolated as a white solid liquid in 66% yield (0.20 mmol, 130 mg); **^{1}H NMR** (400 MHz, $CDCl_3$) δ 8.20 (d, *J* = 8.3 Hz, 1H), 8.12 (d, *J* = 8.4 Hz, 1H), 7.90 (d, *J* = 8.2 Hz, 1H), 7.70 (t, *J* = 7.4 Hz, 1H), 7.64 (t, *J* = 7.5 Hz, 1H), 7.55-7.42 (m, 11H), 7.39-7.34 (m, 1H), 7.25 (s, 1H), 7.21-7.11 (m, 1H), 5.36 (d, *J* = 2.8 Hz, 1H), 3.17-2.99 (m, 3H), 2.84-2.75 (m, 1H), 2.20-1.22 (m, 14H), 1.04 (s, 3H), 0.62 (s, 3H) ppm. **^{13}C {^{1}H} NMR** (101 MHz, $CDCl_3$) δ 161.3, 160.6, 148.1, 147.5, 146.0, 144.9, 138.5, 138.0, 137.7, 131.6, 131.0, 130.6, 129.6, 129.0, 128.8, 128.63, 128.57, 128.3, 128.2, 127.64, 127.60, 127.3, 126.7, 125.7, 125.6, 125.54, 125.49, 122.3, 58.9, 57.0, 48.1, 45.4, 38.5, 37.4, 34.9, 32.3, 32.0, 31.8, 24.9, 24.6, 23.0, 21.8, 13.4 ppm. HRMS (MALDI-TOF) Calcd for $C_{47}H_{45}N_2$ $[M+H]^+$ 637.3577; Found: 637.3578.

(3aS,5bR)-11-chloro-3-(6-chloro-4-phenylquinolin-2-yl)-3a,5b-dimethyl-13-phenyl-2,3,3a,4,5,5a,5b,6,7,15,15a,15b-dodecahydro-1H-cyclopenta[5,6]naphtho[2,1-a]acridine, 10hff

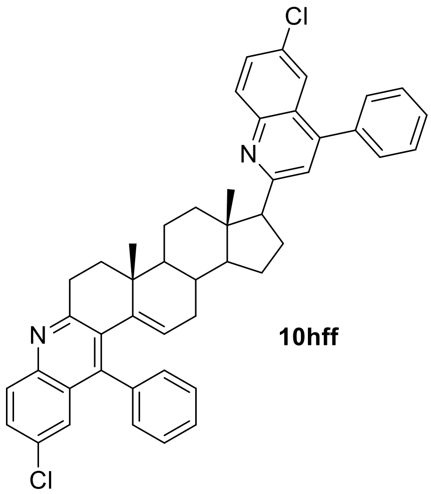

10hff

Prepared from **1h** (0.31 mmol, 100 mg) and **2f** (0.68 mmol, 162 mg) following the general procedure D, and isolated as a white solid in 43% yield (0.14 mmol, 97 mg); mp:217-218 °C; **^{1}H NMR** (400 MHz, $CDCl_3$) δ 8.09 (d, *J* = 9.0 Hz, 1H), 7.98 (d, *J* = 8.9 Hz, 1H), 7.85 (d, *J* = 2.3 Hz, 1H), 7.63 (dd, *J* = 9.0, 2.3 Hz, 1H), 7.60-7.41 (m, 11H), 7.24 (s, 1H), 7.18-7.10 (m, 1H), 5.36-5.33 (m, 1H), 3.13-2.96 (m, 3H), 2.86-2.70 (m, 1H), 2.17-1.20 (m, 14H), 1.03 (s, 3H), 0.59 (s, 3H) ppm. **^{13}C {^{1}H} NMR** (101 MHz, $CDCl_3$) δ 161.7, 161.1, 146.7, 146.6, 144.8, 143.7, 138.0, 137.9, 137.1, 131.49, 131.46, 131.41, 131.36, 131.2, 130.1, 129.8, 129.5, 129.3, 129.0, 128.8 128.5, 128.4, 127.9, 127.6, 126.2, 125.4, 124.3, 123.1, 58.9, 57.0, 48.1, 45.4, 38.5, 37.4, 34.8, 32.2, 32.0, 29.7, 24.8, 24.5, 23.1, 21.7, 13.3 ppm. HRMS (MALDI-TOF) Calcd for $C_{47}H_{43}Cl_2N_2$ $[M+H]^+$ 705.2798; Found: 705.2793.

1-((6aR,8aS)-6a,8a-dimethyl-6,6a,6b,7,8,8a,9,10,11,11a,11b,12-dodecahydro-5H-cyclopenta[5,6]naphtho[2,1-f]quinolin-9-yl)ethan-1-one. 11i[1]

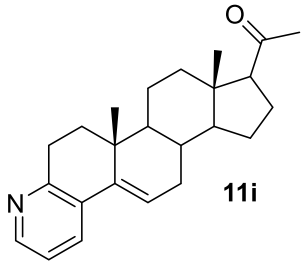

11i

Prepared from **1h** (0.4 mmol, 126 mg) and Propargylamine (0.8 mmol, 45 mg) and isolated as a white solid liquid in 67% yield (0.27 mmol, 94 mg); **^{1}H NMR** (400 MHz, $CDCl_3$) δ 8.34 – 8.31 (m, 1H), 7.79 – 7.66 (m, 1H), 7.04-6.98 (m, 1H), 6.10-6.07 (m, 1H), 2.98-2.92 (m, 2H), 2.54-2.48 (m, 1H), 2.29 – 1.99 (m, 7H), 1.80 – 1.40 (m, 8H), 1.25 – 1.06 (m, 3H), 0.90 (s, 3H), 0.64-0.61 (m, 3H) ppm. **^{13}C {^{1}H} NMR** (101 MHz, $CDCl_3$) δ 209.2, 154.6, 147.5, 139.5, 132.4, 130.4, 121.8, 121.3, 63.6, 56.8, 48.9, 44.0, 38.8, 35.5, 34.1, 32.5, 31.5, 31.3, 29.0, 24.4, 22.8, 21.2, 18.5, 13.3 ppm. HRMS (ESI-Orbitrap) Calcd for $C_{24}H_{32}NO$ $[M+H]^+$ 350.2478; Found: 350.2482.

(6aR,8aS)-6a,8a-dimethyl-9-(4-methylquinolin-2-yl)-6,6a,6b,7,8,8a,9,10,11,11a,11b,12-dodecahydro-5H-cyclopenta[5,6]naphtho[2,1-f]quinoline, 12ia

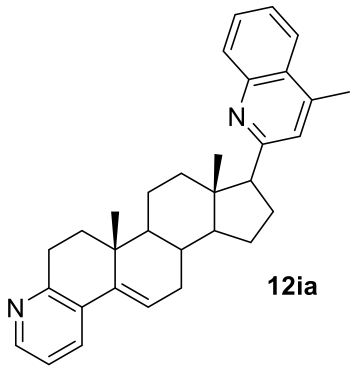

12ia

Prepared from **11i** (0.4 mmol, 140 mg) and **2a** (0.44 mmol, 60 mg) following general procedure **A** and isolated as a white solid liquid in 87% yield (0.35 mmol, 156 mg); **^{1}H NMR** (400 MHz, $CDCl_3$) δ 8.48-8.34 (m, 1H), 8.15-8.10 (m, 1H), 7.98-7.93 (m 1H), 7.81-7.76 (m, 1H), 7.69-7.65 (m, 1H), 7.53-7.48 (m, 1H), 7.16-7.13 (m, 1H), 7.10-7.05 (m, 1H), 6.18 (m, 1H), 3.10-2.91 (m, 3H), 2.70 (s, 3H), 2.38 (s, 1H), 2.19–1.87 (m, 4H), 1.80-1.06 (m, 9H), 0.97-0.84 (m, 4H), 0.58 (s, 3H) ppm. **^{13}C {^{1}H} NMR** (101 MHz, $CDCl_3$) δ 164.4, 161.4, 154.7, 147.4, 143.2, 139.6, 132.6, 130.7, 129.7, 128.8, 127.0, 125.4, 123.6, 122.8, 122.3, 121.4, 58.7, 56.8, 49.2, 45.3, 38.2, 35.6, 34.1, 32.7, 31.7, 29.7, 29.0, 24.9, 21.0, 18.9, 18.5, 13.2 ppm. HRMS (ESI-Orbitrap) Calcd for $C_{32}H_{37}N_2$ $[M+H]^+$ 449.2951; Found: 449.2955.

NMR Spectra

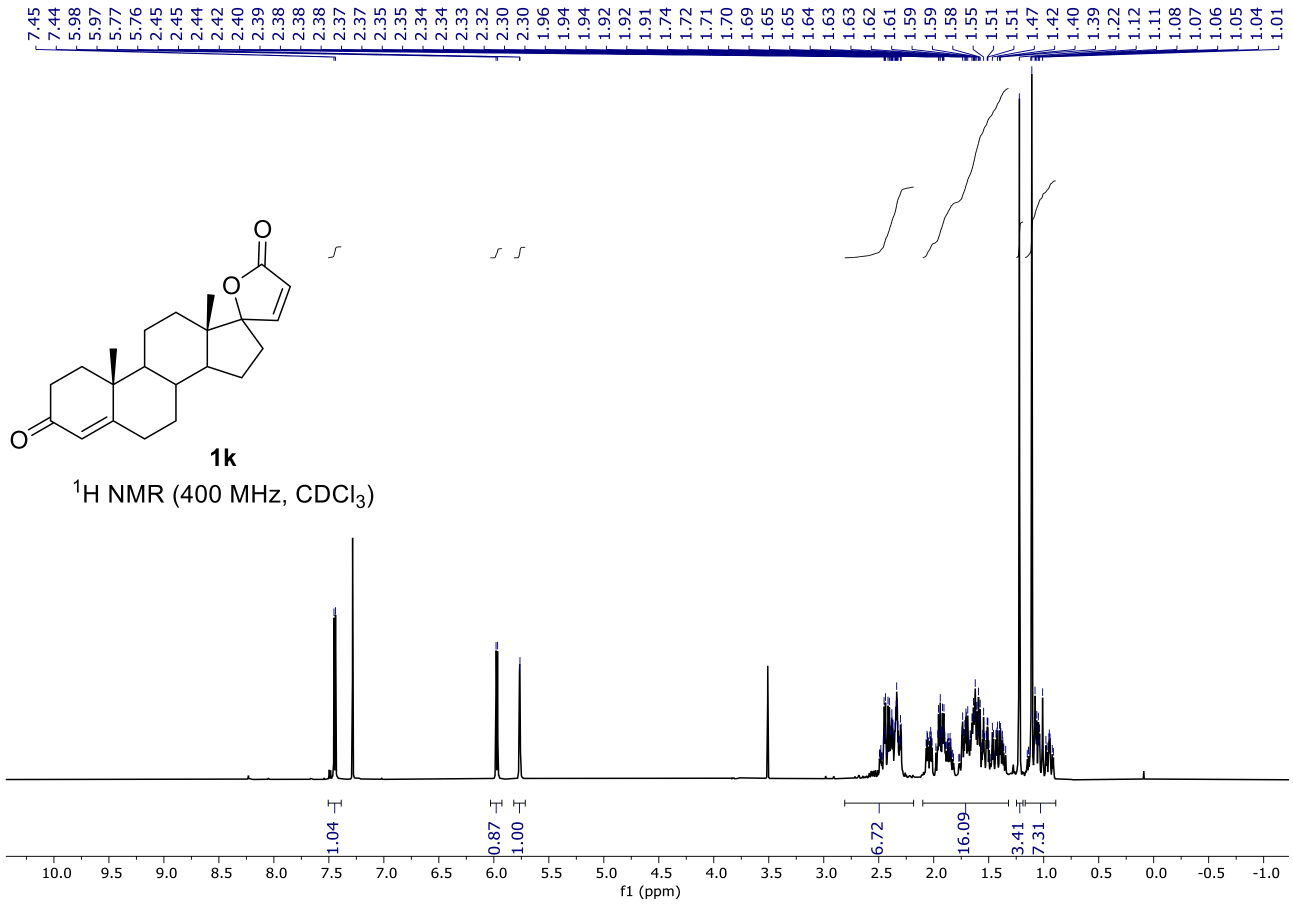
7.45 7.44 5.98 5.97 5.77 5.76 2.45 2.45 2.44 2.42 2.40 2.39 2.38 2.38 2.38 2.37 2.37 2.35 2.35 2.34 2.34 2.33 2.32 2.30 2.30 1.96 1.94 1.94 1.92 1.92 1.91 1.74 1.72 1.71 1.70 1.69 1.65 1.65 1.64 1.63 1.63 1.62 1.61 1.59 1.59 1.58 1.55 1.51 1.51 1.47 1.42 1.40 1.39 1.22 1.12 1.11 1.08 1.07 1.06 1.05 1.04 1.01
O
O
O
1k
^{1}H NMR (400 MHz, $CDCl_3$)
1.04
0.87
1.00
6.72
16.09
3.41
7.31
10.0 9.5 9.0 8.5 8.0 7.5 7.0 6.5 6.0 5.5 5.0 4.5 4.0 3.5 3.0 2.5 2.0 1.5 1.0 0.5 0.0 -0.5 -1.0
f1 (ppm)

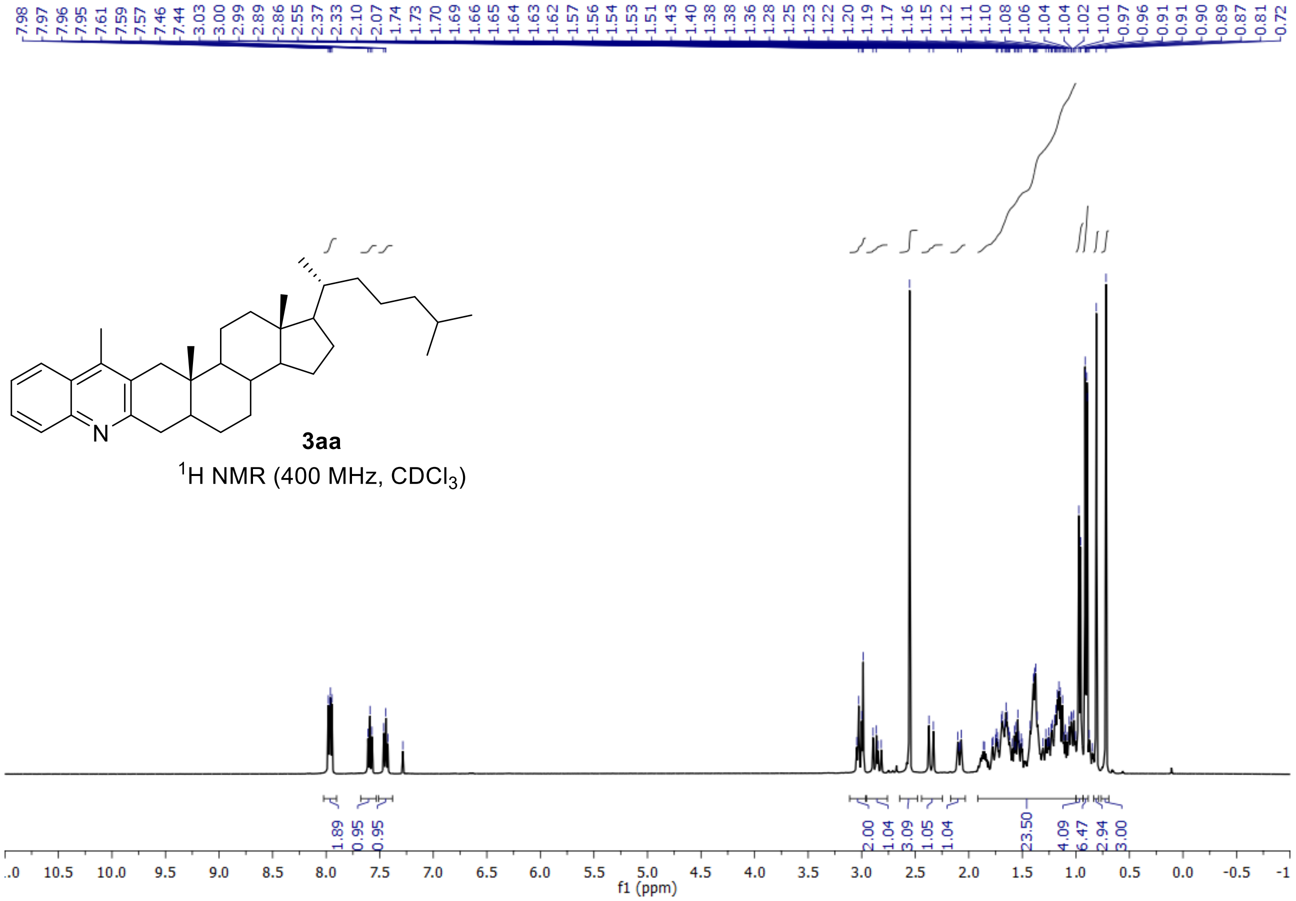
7.98 7.97 7.96 7.95 7.61 7.59 7.57 7.46 7.44 3.03 3.00 2.99 2.89 2.86 2.55 2.37 2.33 2.10 2.07 1.74 1.73 1.70 1.69 1.66 1.65 1.64 1.63 1.62 1.57 1.56 1.54 1.53 1.51 1.43 1.40 1.38 1.38 1.36 1.28 1.25 1.23 1.22 1.20 1.19 1.17 1.16 1.15 1.12 1.11 1.10 1.08 1.06 1.04 1.04 1.02 1.01 0.97 0.96 0.91 0.91 0.90 0.89 0.87 0.81 0.72
N
3aa
^{1}H NMR (400 MHz, $CDCl_3$)
1.89
0.95
0.95
2.00
1.04
3.09
1.05
1.04
23.50
4.09
6.47
2.94
3.00
f1 (ppm)

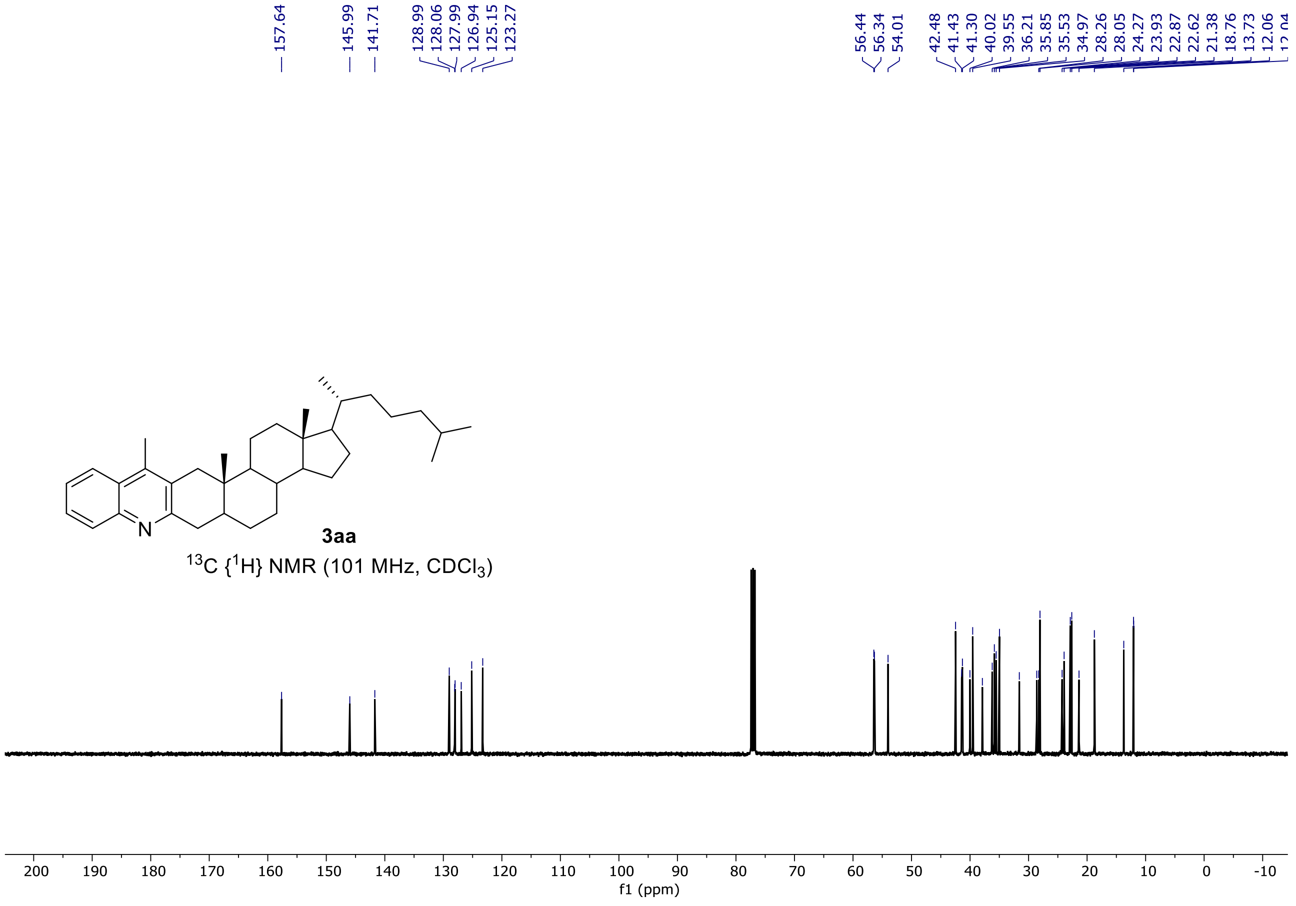
157.64
145.99
141.71
128.99 128.06 127.99 126.94 125.15 123.27
56.44 56.34 54.01
42.48 41.43 41.30 40.02 39.55 36.21 35.85 35.53 34.97 28.26 28.05 24.27 23.93 22.87 22.62 21.38 18.76 13.73 12.06
N
3aa
^{13}C {^{1}H} NMR (101 MHz, $CDCl_3$)
f1 (ppm)

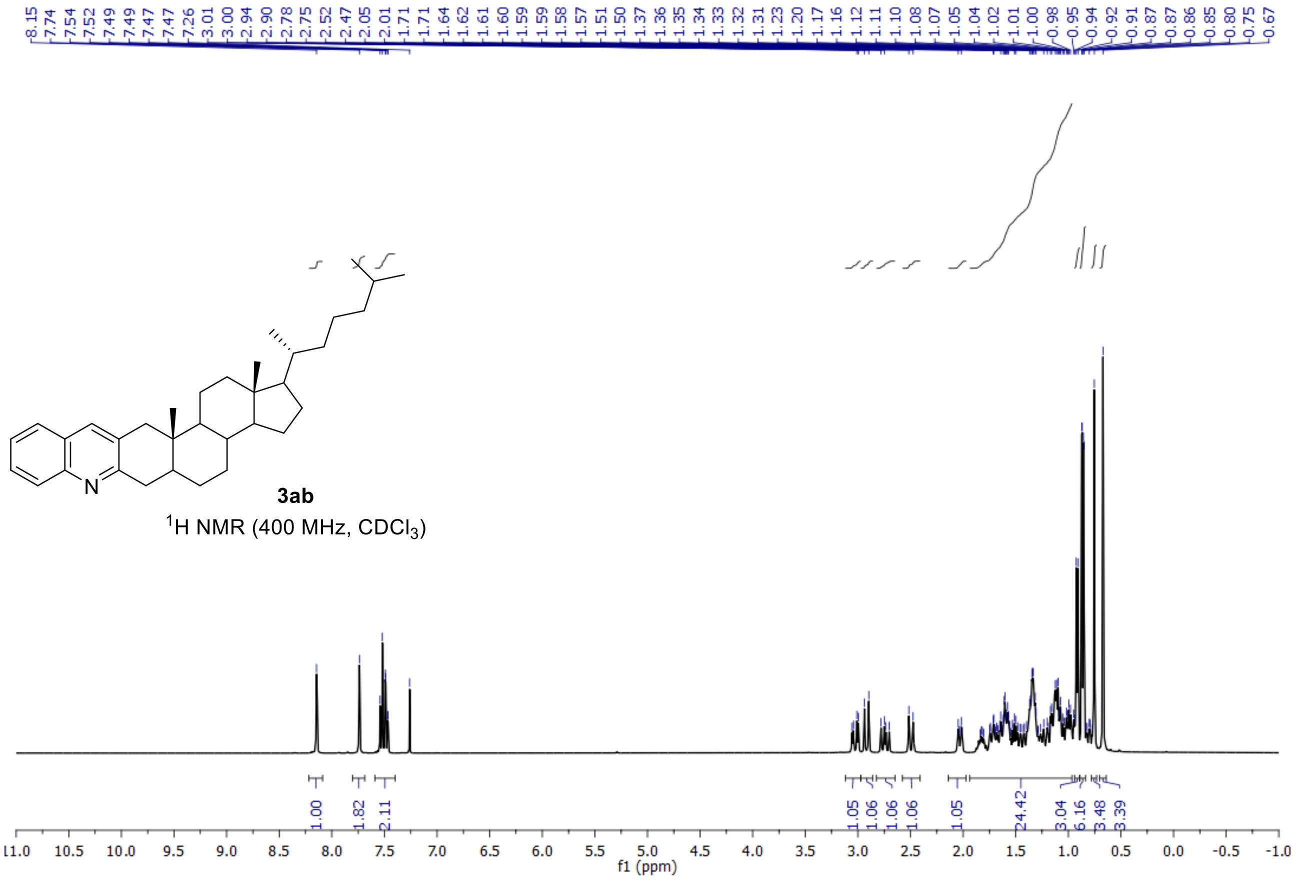
8.15
7.74
7.54
7.52
7.49
7.49
7.47
7.47
7.26
3.01
3.00
2.94
2.90
2.78
2.75
2.52
2.47
2.05
2.01
1.71
1.71
1.64
1.62
1.61
1.60
1.59
1.59
1.58
1.57
1.51
1.50
1.37
1.36
1.35
1.34
1.33
1.32
1.31
1.23
1.20
1.17
1.16
1.12
1.11
1.10
1.08
1.07
1.05
1.04
1.02
1.01
1.00
0.98
0.95
0.94
0.92
0.91
0.87
0.87
0.86
0.85
0.80
0.75
0.67
3ab
^{1}H NMR (400 MHz, $CDCl_3$)
1.00
1.82
2.11
1.05
1.06
1.06
1.06
1.05
24.42
3.04
6.16
3.48
3.39
f1 (ppm)

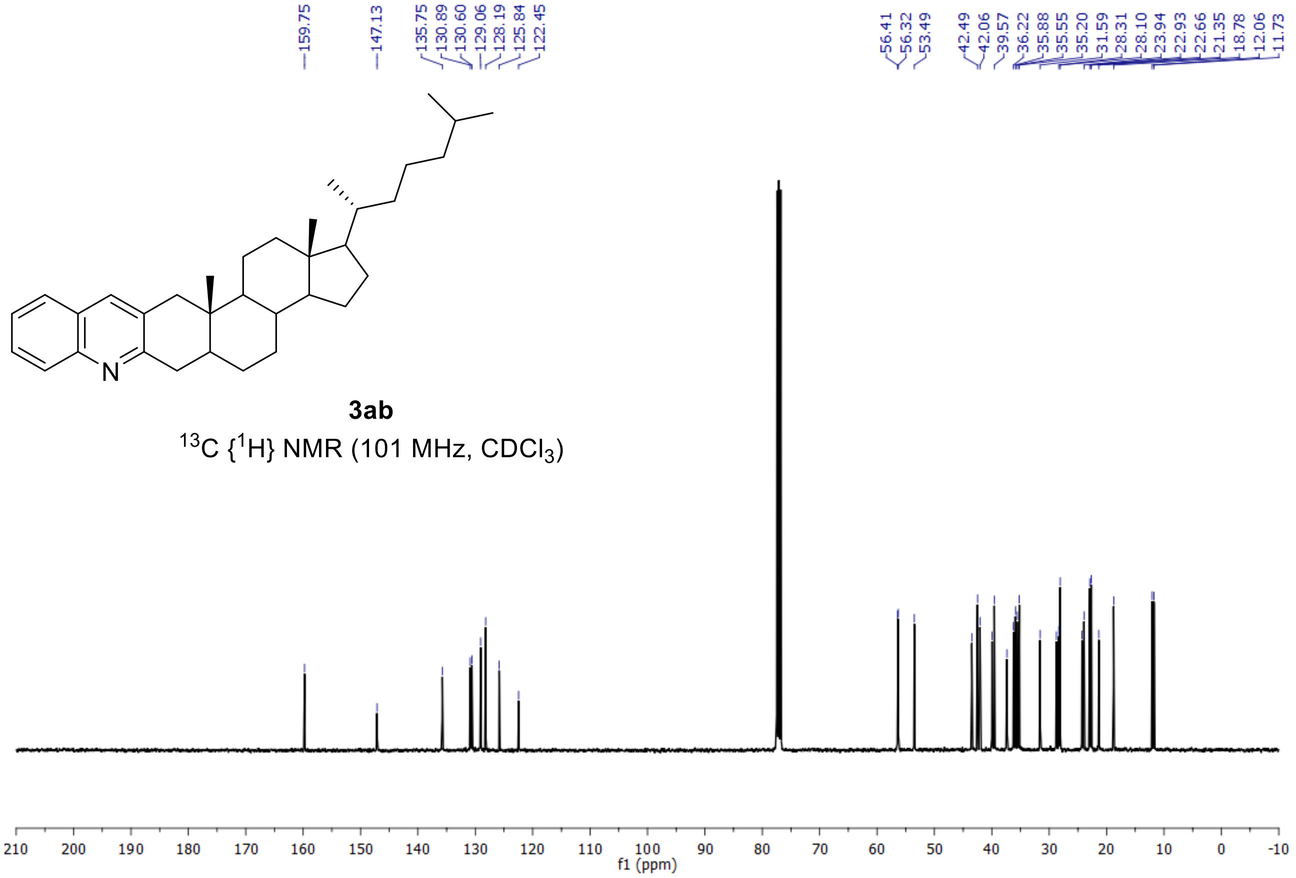
159.75
147.13
135.75
130.89
130.60
129.06
128.19
125.84
122.45
56.41
56.32
53.49
42.49
42.06
39.57
36.22
35.88
35.55
35.20
31.59
28.31
28.10
23.94
22.93
22.66
21.35
18.78
12.06
11.73
3ab
^{13}C {^{1}H} NMR (101 MHz, $CDCl_3$)
f1 (ppm)

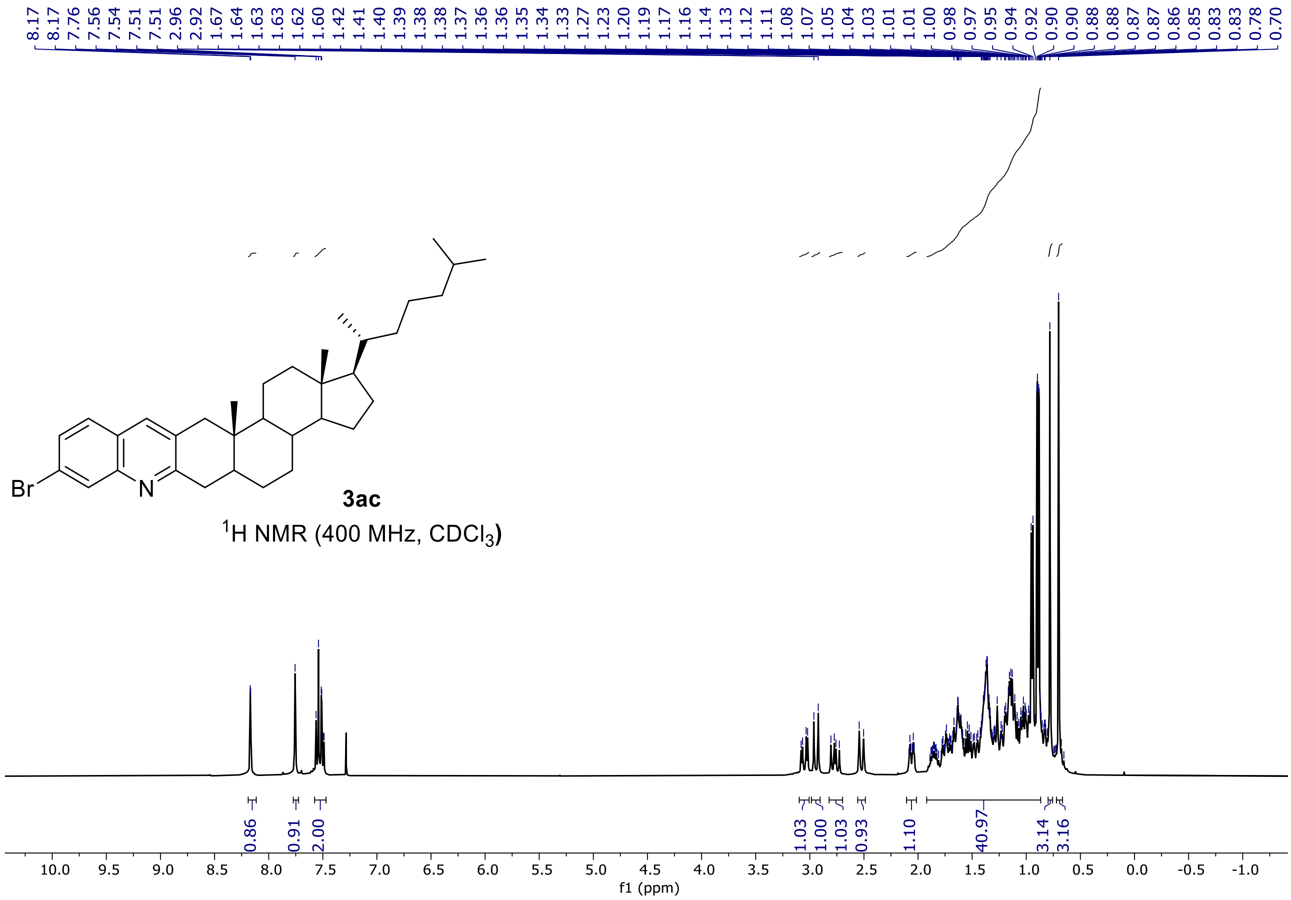
8.17 8.17 7.76 7.56 7.54 7.51 7.51 2.96 2.92 1.67 1.64 1.63 1.63 1.62 1.60 1.42 1.41 1.40 1.39 1.38 1.38 1.37 1.36 1.36 1.35 1.34 1.33 1.27 1.23 1.20 1.19 1.17 1.16 1.14 1.13 1.12 1.11 1.08 1.07 1.05 1.04 1.03 1.01 1.01 1.00 0.98 0.97 0.95 0.94 0.92 0.90 0.90 0.88 0.87 0.87 0.86 0.85 0.83 0.83 0.78 0.70
Br
N
3ac
^{1}H NMR (400 MHz, $CDCl_3$)
0.86 0.91 2.00 1.03 1.00 1.03 0.93 1.10 40.97 3.14 3.16
10.0 9.5 9.0 8.5 8.0 7.5 7.0 6.5 6.0 5.5 5.0 4.5 4.0 3.5 3.0 2.5 2.0 1.5 1.0 0.5 0.0 -0.5 -1.0
f1 (ppm)

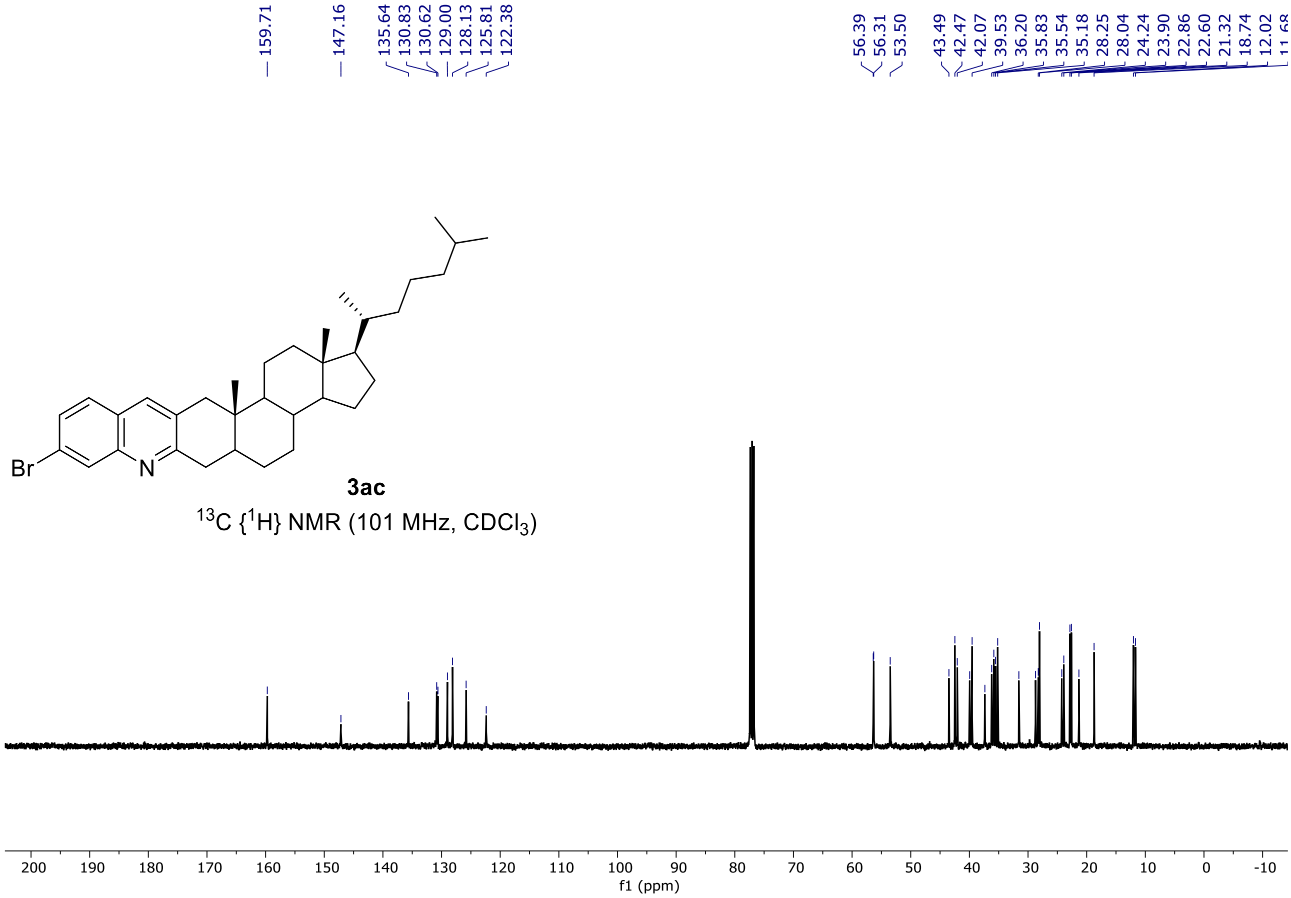
159.71 147.16 135.64 130.83 130.62 129.00 128.13 125.81 122.38 56.39 56.31 53.50 43.49 42.47 42.07 39.53 36.20 35.83 35.54 35.18 28.25 28.04 24.24 23.90 22.86 22.60 21.32 18.74 12.02
Br
N
3ac
^{13}C {^{1}H} NMR (101 MHz, $CDCl_3$)
200 190 180 170 160 150 140 130 120 110 100 90 80 70 60 50 40 30 20 10 0 -10
f1 (ppm)

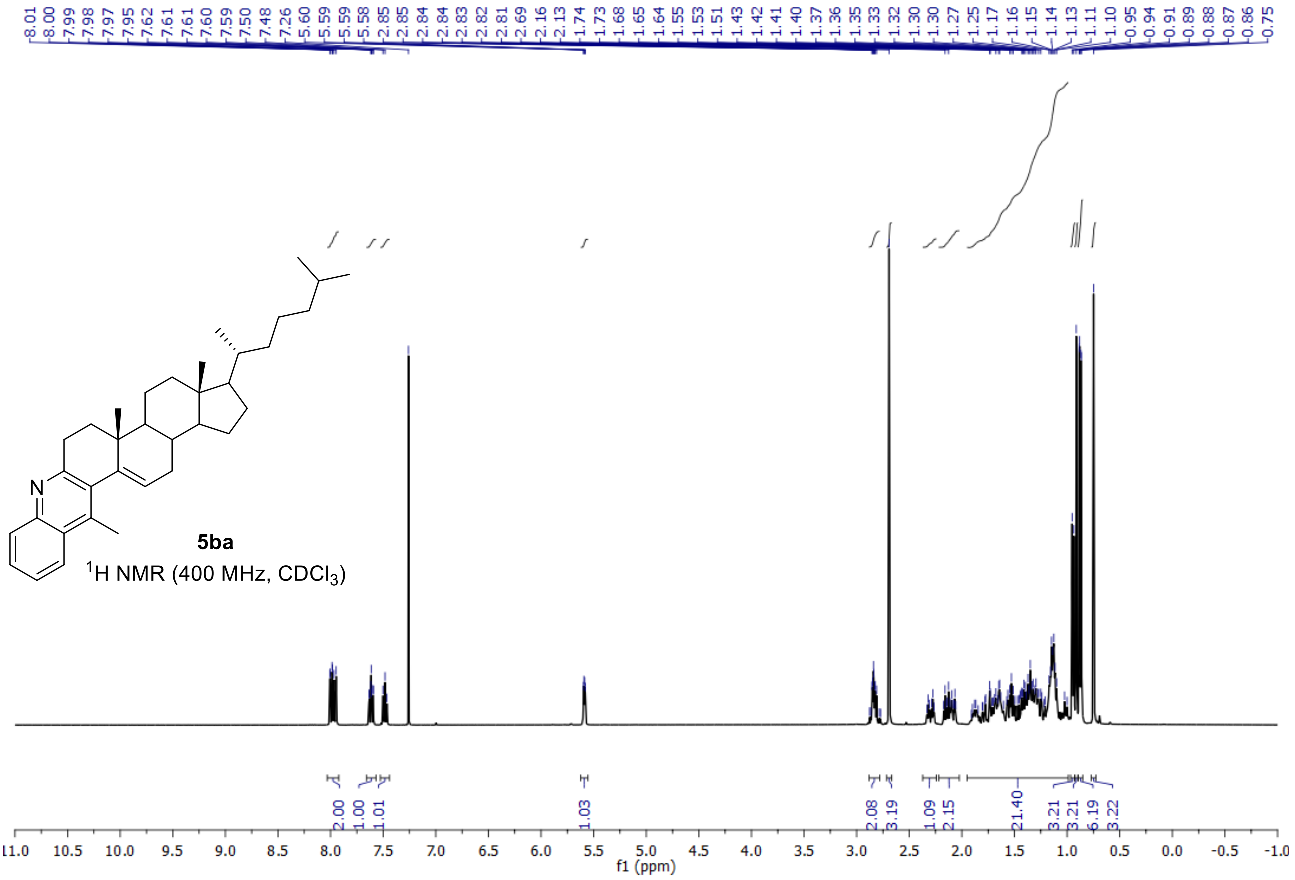
8.01
8.00
7.99
7.98
7.97
7.95
7.62
7.61
7.61
7.60
7.59
7.50
7.48
7.26
5.60
5.59
5.59
5.58
2.85
2.85
2.84
2.84
2.83
2.82
2.81
2.69
2.16
2.13
1.74
1.73
1.68
1.65
1.64
1.55
1.53
1.51
1.43
1.42
1.41
1.40
1.37
1.36
1.35
1.33
1.32
1.30
1.30
1.27
1.25
1.17
1.16
1.15
1.14
1.13
1.11
1.10
0.95
0.94
0.91
0.89
0.88
0.87
0.86
0.75
N
5ba
1H NMR (400 MHz, CDCl3)
2.00
1.00
1.01
1.03
2.08
3.19
1.09
2.15
21.40
3.21
3.21
6.19
3.22
f1 (ppm)

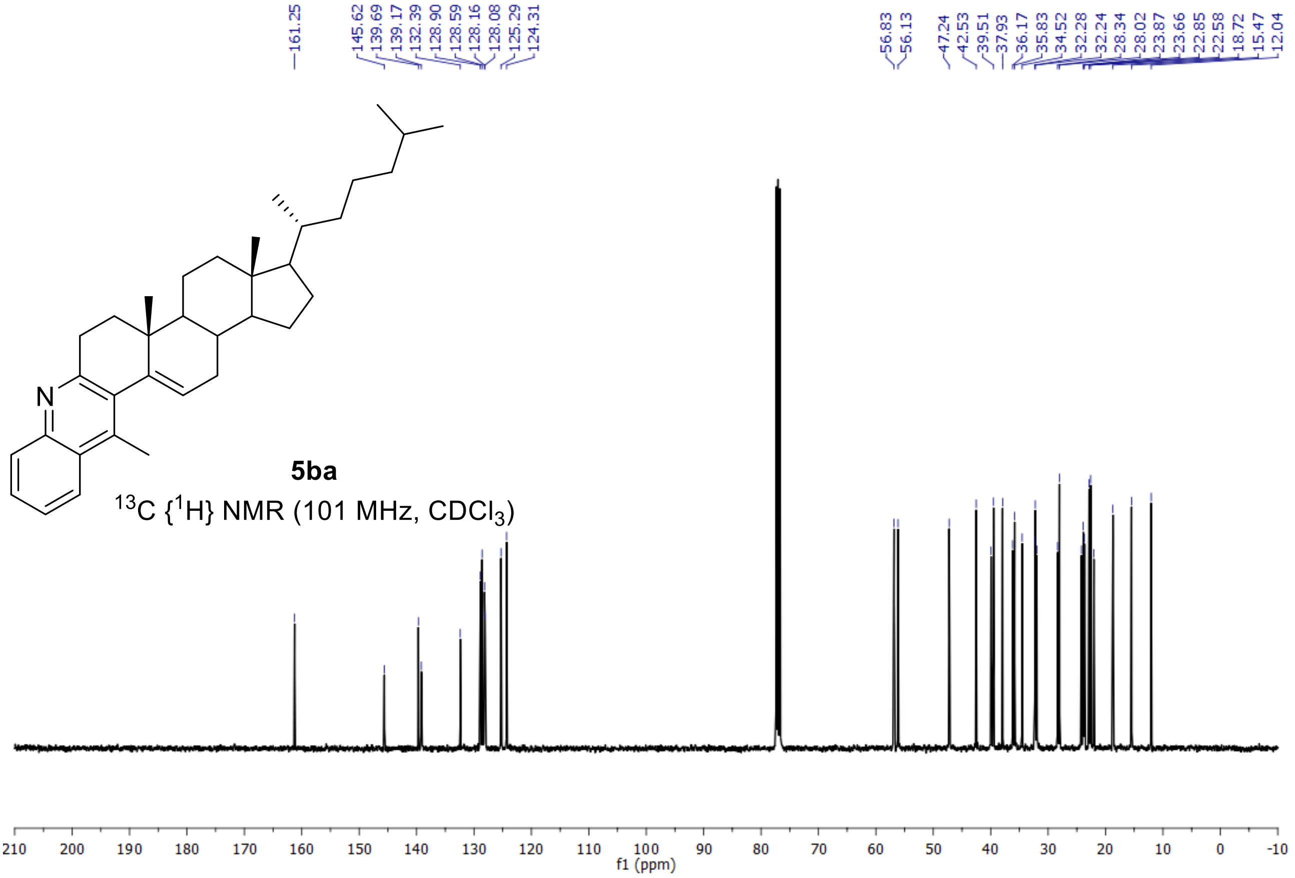
161.25
145.62
139.69
139.17
132.39
128.90
128.59
128.16
128.08
125.29
124.31
56.83
56.13
47.24
42.53
39.51
37.93
36.17
35.83
34.52
32.28
32.24
28.34
28.02
23.87
23.66
22.85
22.58
18.72
15.47
12.04
N
5ba
13C {1H} NMR (101 MHz, CDCl3)
f1 (ppm)

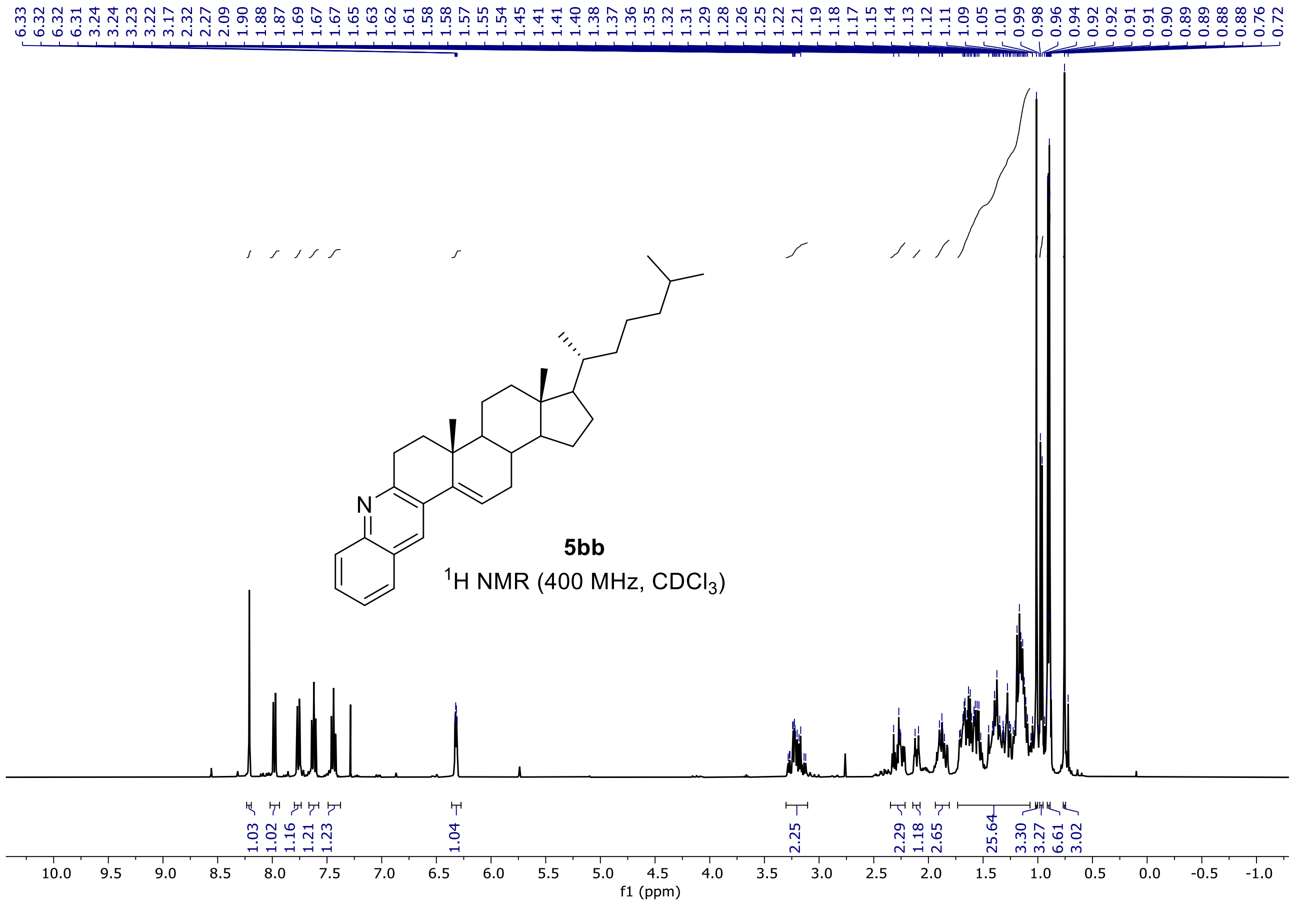
N
5bb
^{1}H NMR (400 MHz, $CDCl_3$)
f1 (ppm)

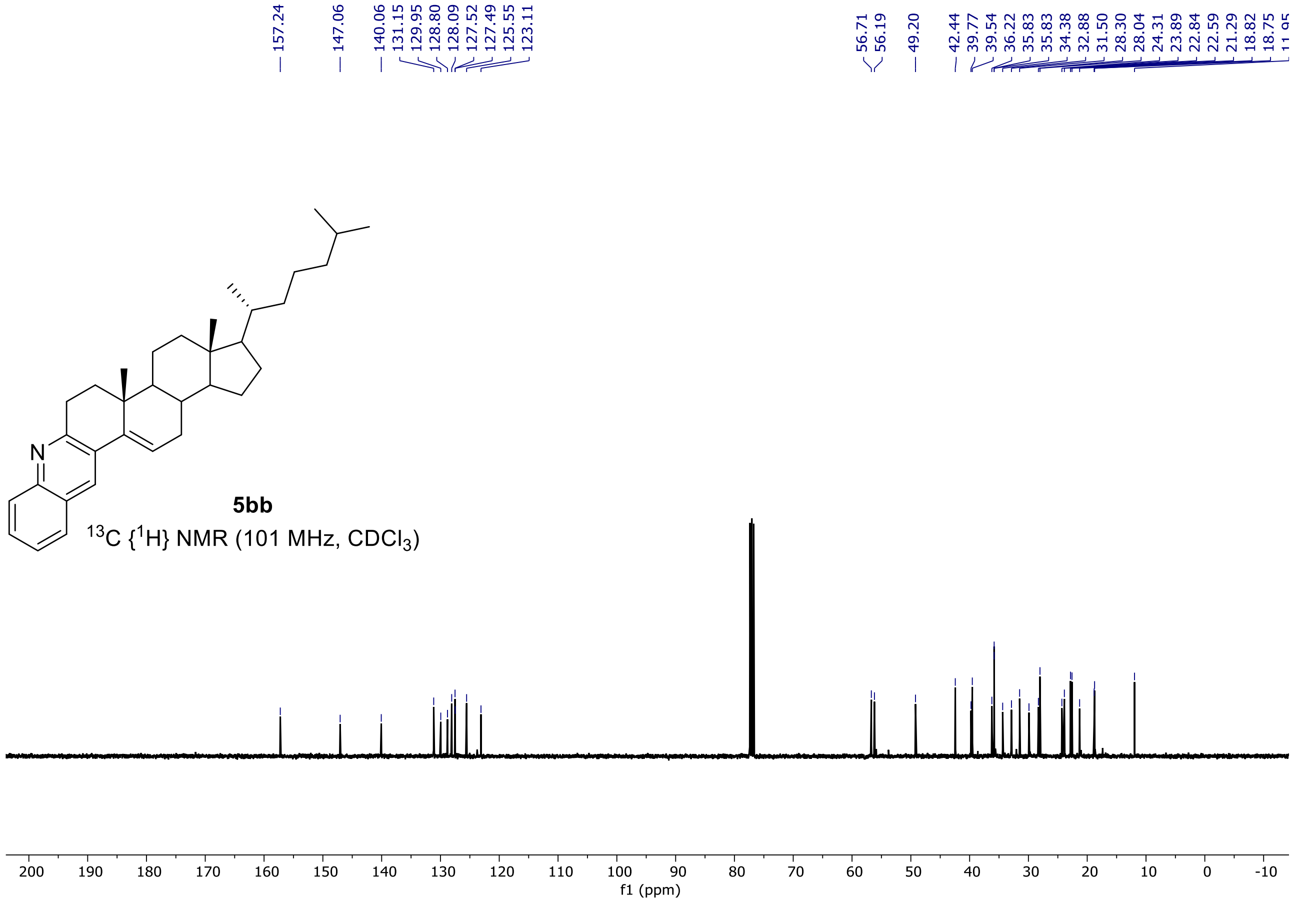
N
5bb
^{13}C {^{1}H} NMR (101 MHz, $CDCl_3$)
f1 (ppm)

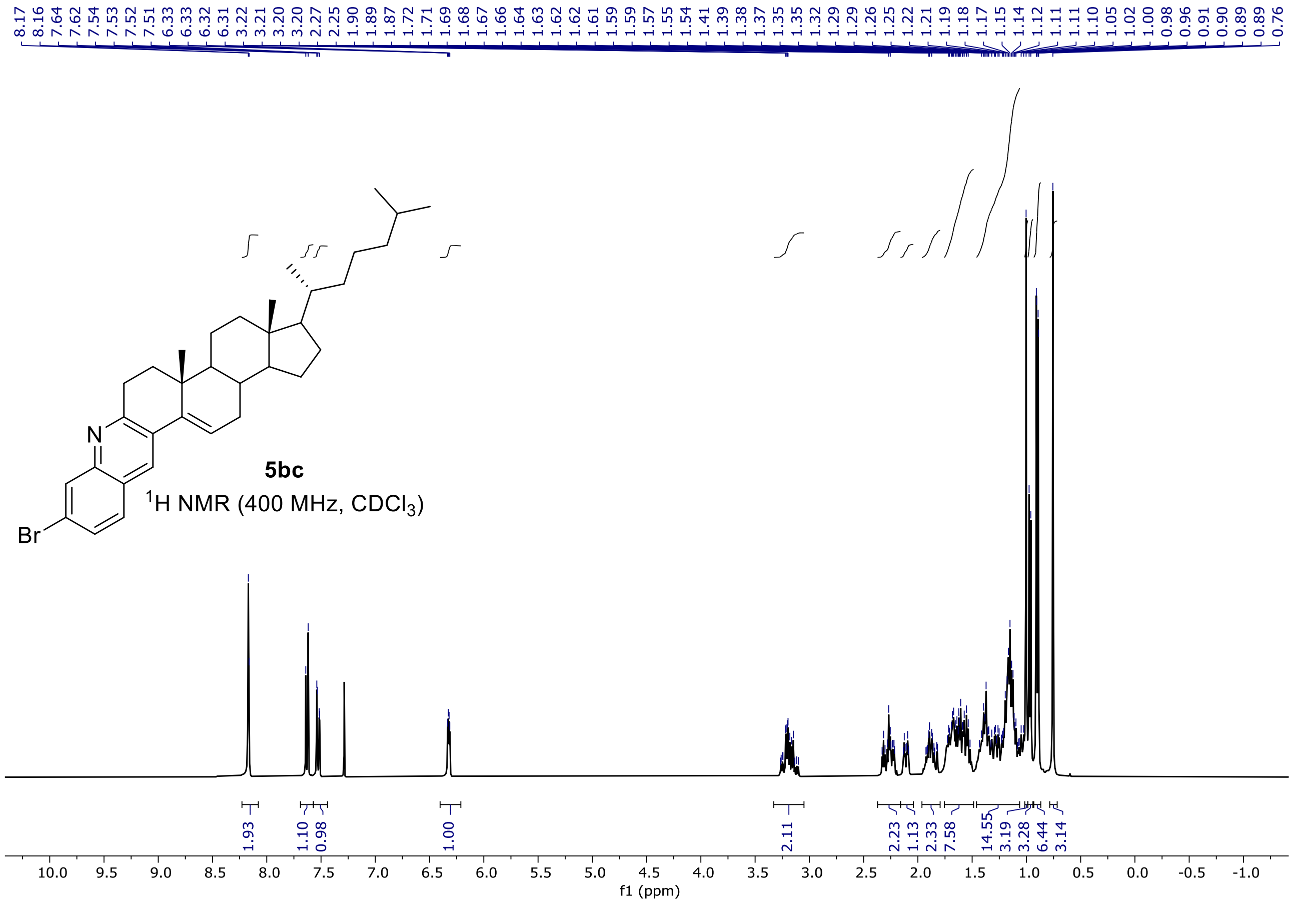
8.17 8.16 7.64 7.62 7.54 7.53 7.52 7.51 6.33 6.33 6.32 6.31 3.22 3.21 3.20 3.20 2.27 2.25 1.90 1.89 1.87 1.72 1.71 1.69 1.68 1.67 1.66 1.64 1.63 1.62 1.62 1.61 1.59 1.59 1.57 1.55 1.54 1.41 1.39 1.38 1.37 1.35 1.35 1.32 1.29 1.29 1.26 1.25 1.22 1.21 1.19 1.18 1.17 1.15 1.14 1.12 1.11 1.11 1.10 1.05 1.02 1.00 0.98 0.96 0.91 0.90 0.89 0.89 0.76
N
Br
5bc
^{1}H NMR (400 MHz, CDCl$_3$)
1.93 1.10 0.98 1.00 2.11 2.23 1.13 2.33 7.58 14.55 3.19 3.28 6.44 3.14
10.0 9.5 9.0 8.5 8.0 7.5 7.0 6.5 6.0 5.5 5.0 4.5 4.0 3.5 3.0 2.5 2.0 1.5 1.0 0.5 0.0 -0.5 -1.0
f1 (ppm)

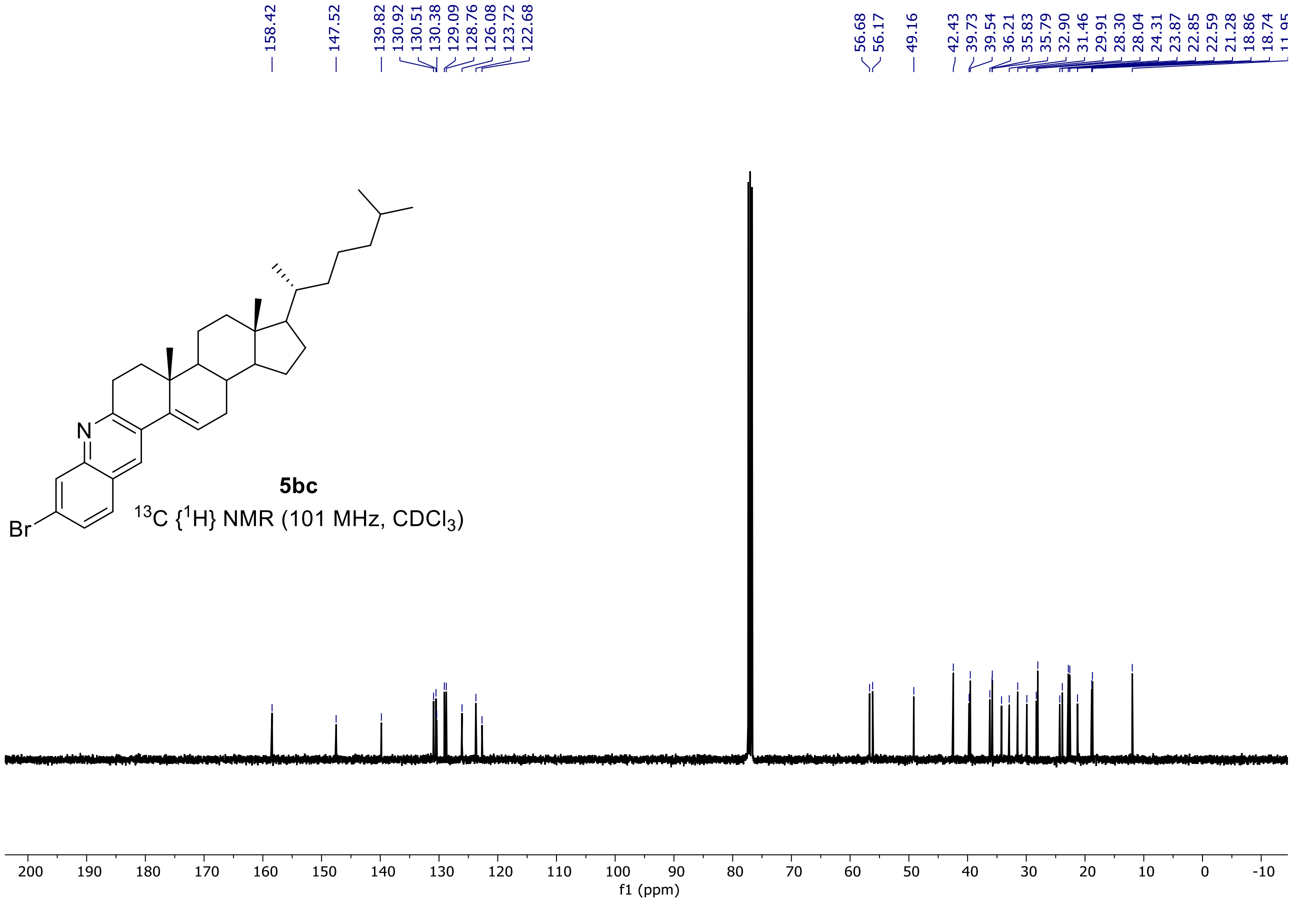
158.42 147.52 139.82 130.92 130.51 130.38 129.09 128.76 126.08 123.72 122.68 56.68 56.17 49.16 42.43 39.73 39.54 36.21 35.83 35.79 32.90 31.46 29.91 28.30 28.04 24.31 23.87 22.85 22.59 21.28 18.86 18.74
N
Br
5bc
^{13}C {^{1}H} NMR (101 MHz, CDCl$_3$)
200 190 180 170 160 150 140 130 120 110 100 90 80 70 60 50 40 30 20 10 0 -10
f1 (ppm)

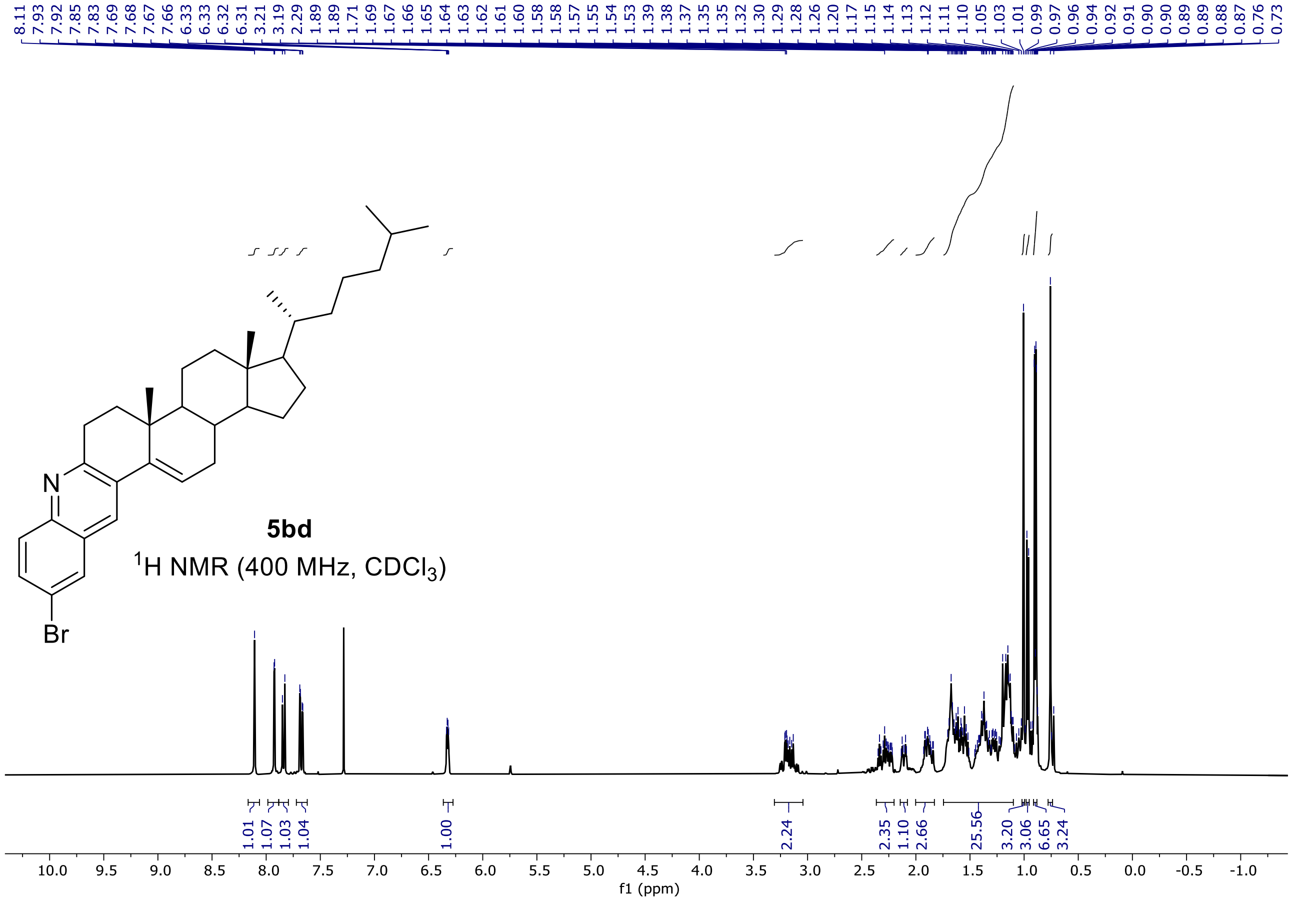
8.11 7.93 7.92 7.85 7.83 7.69 7.68 7.67 7.66 6.33 6.33 6.32 6.31 3.21 3.19 2.29 1.89 1.89 1.71 1.69 1.67 1.66 1.65 1.64 1.63 1.62 1.61 1.60 1.58 1.58 1.57 1.55 1.54 1.53 1.39 1.38 1.37 1.35 1.35 1.32 1.30 1.29 1.28 1.26 1.20 1.17 1.15 1.14 1.13 1.12 1.11 1.10 1.05 1.03 1.01 0.99 0.97 0.96 0.94 0.92 0.91 0.90 0.89 0.89 0.88 0.87 0.76 0.73
N
Br
5bd
^{1}H NMR (400 MHz, $CDCl_3$)
1.01 1.07 1.03 1.04 1.00 2.24 2.35 1.10 2.66 25.56 3.20 3.06 6.65 3.24
10.0 9.5 9.0 8.5 8.0 7.5 7.0 6.5 6.0 5.5 5.0 4.5 4.0 3.5 3.0 2.5 2.0 1.5 1.0 0.5 0.0 -0.5 -1.0
f1 (ppm)

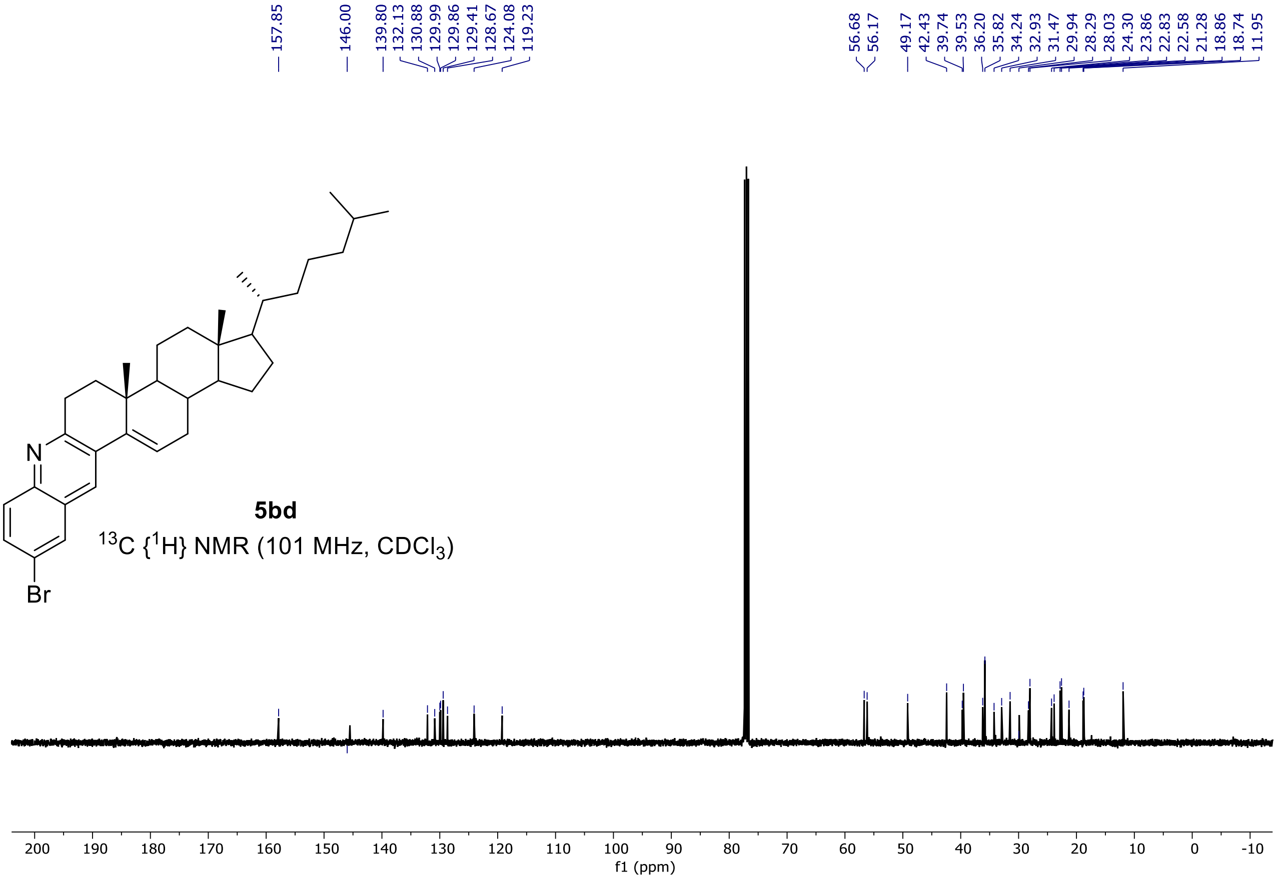
157.85 146.00 139.80 132.13 130.88 129.99 129.86 129.41 128.67 124.08 119.23
56.68 56.17 49.17 42.43 39.74 39.53 36.20 35.82 34.24 32.93 31.47 29.94 28.29 28.03 24.30 23.86 22.83 22.58 21.28 18.86 18.74 11.95
N
Br
5bd
^{13}C {^{1}H} NMR (101 MHz, $CDCl_3$)
200 190 180 170 160 150 140 130 120 110 100 90 80 70 60 50 40 30 20 10 0 -10
f1 (ppm)

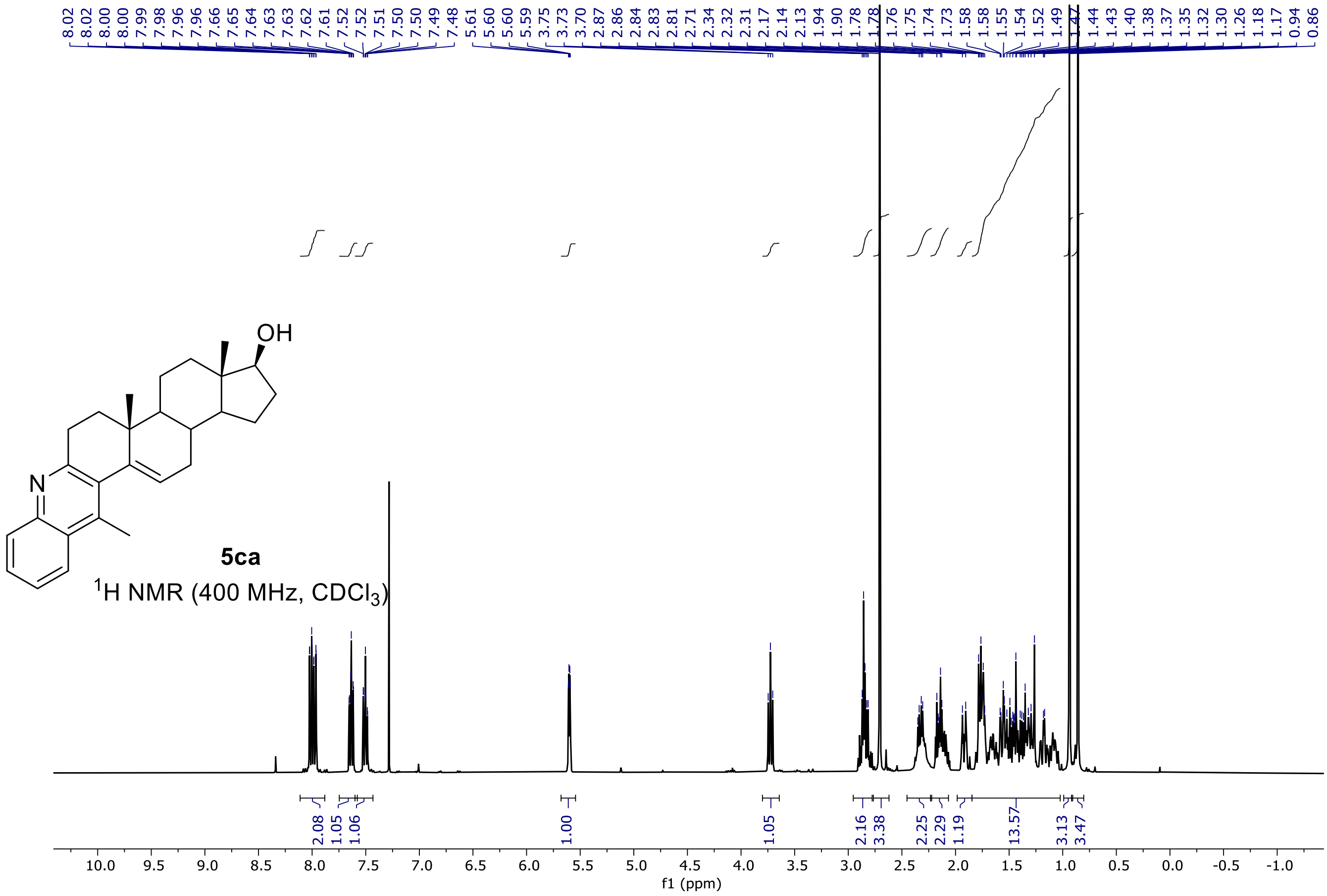
8.02 8.02 8.00 8.00 7.99 7.98 7.96 7.96 7.66 7.65 7.64 7.63 7.63 7.62 7.61 7.52 7.52 7.51 7.50 7.49 7.48 5.61 5.60 5.60 5.59 3.75 3.73 3.70 2.87 2.86 2.84 2.83 2.81 2.71 2.34 2.32 2.31 2.17 2.14 2.13 1.94 1.90 1.78 1.78 1.76 1.75 1.74 1.73 1.58 1.58 1.55 1.54 1.52 1.49 1.47 1.44 1.43 1.40 1.38 1.37 1.35 1.32 1.30 1.26 1.18 1.17 0.94 0.86
OH
N
5ca
1H NMR (400 MHz, CDCl3)
2.08 1.05 1.06 1.00 1.05 2.16 3.38 2.25 2.29 1.19 13.57 3.13 3.47
f1 (ppm)

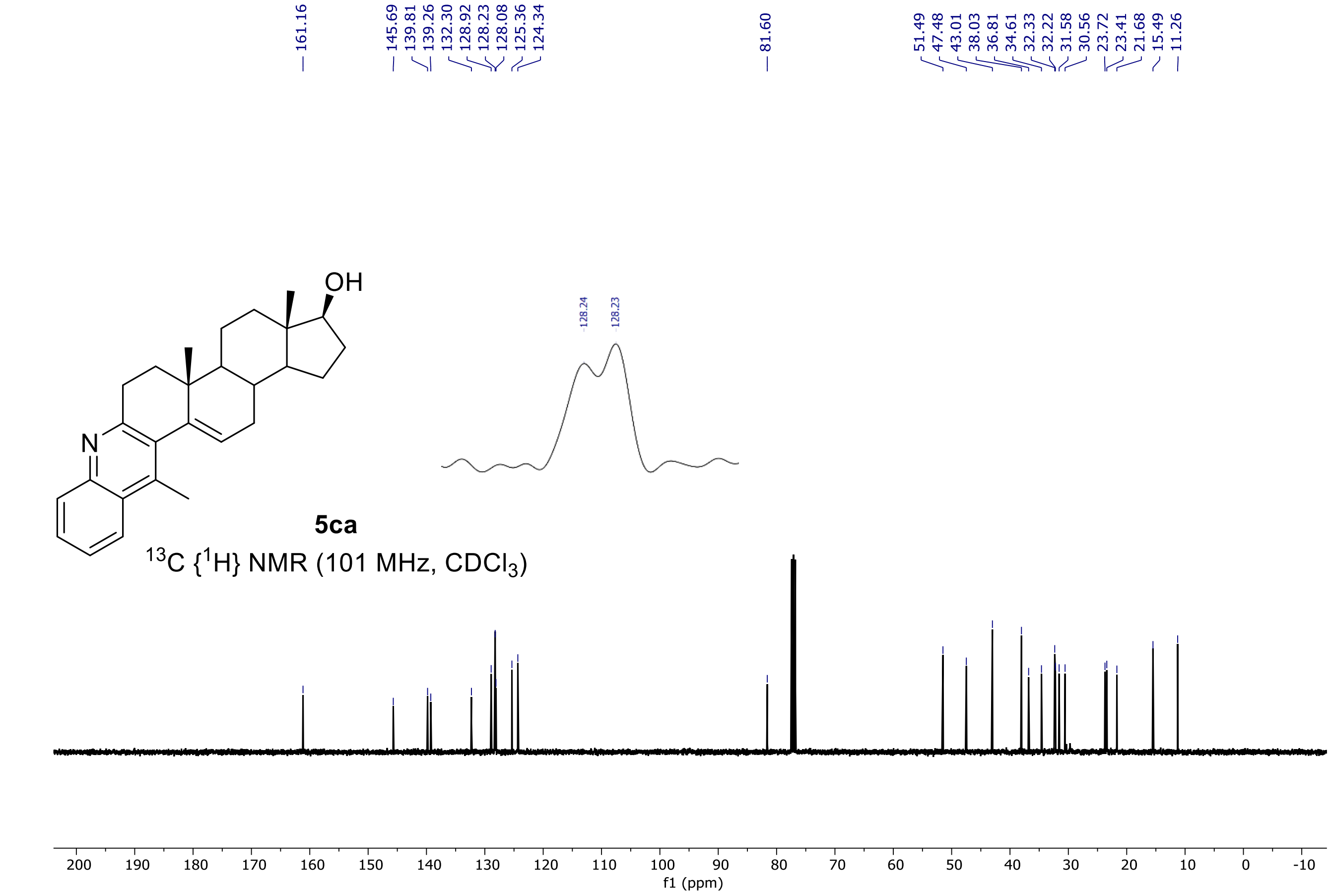
161.16 145.69 139.81 139.26 132.30 128.92 128.23 128.08 125.36 124.34 81.60 51.49 47.48 43.01 38.03 36.81 34.61 32.33 32.22 31.58 30.56 23.72 23.41 21.68 15.49 11.26
128.24 128.23
OH
N
5ca
13C {1H} NMR (101 MHz, CDCl3)
f1 (ppm)

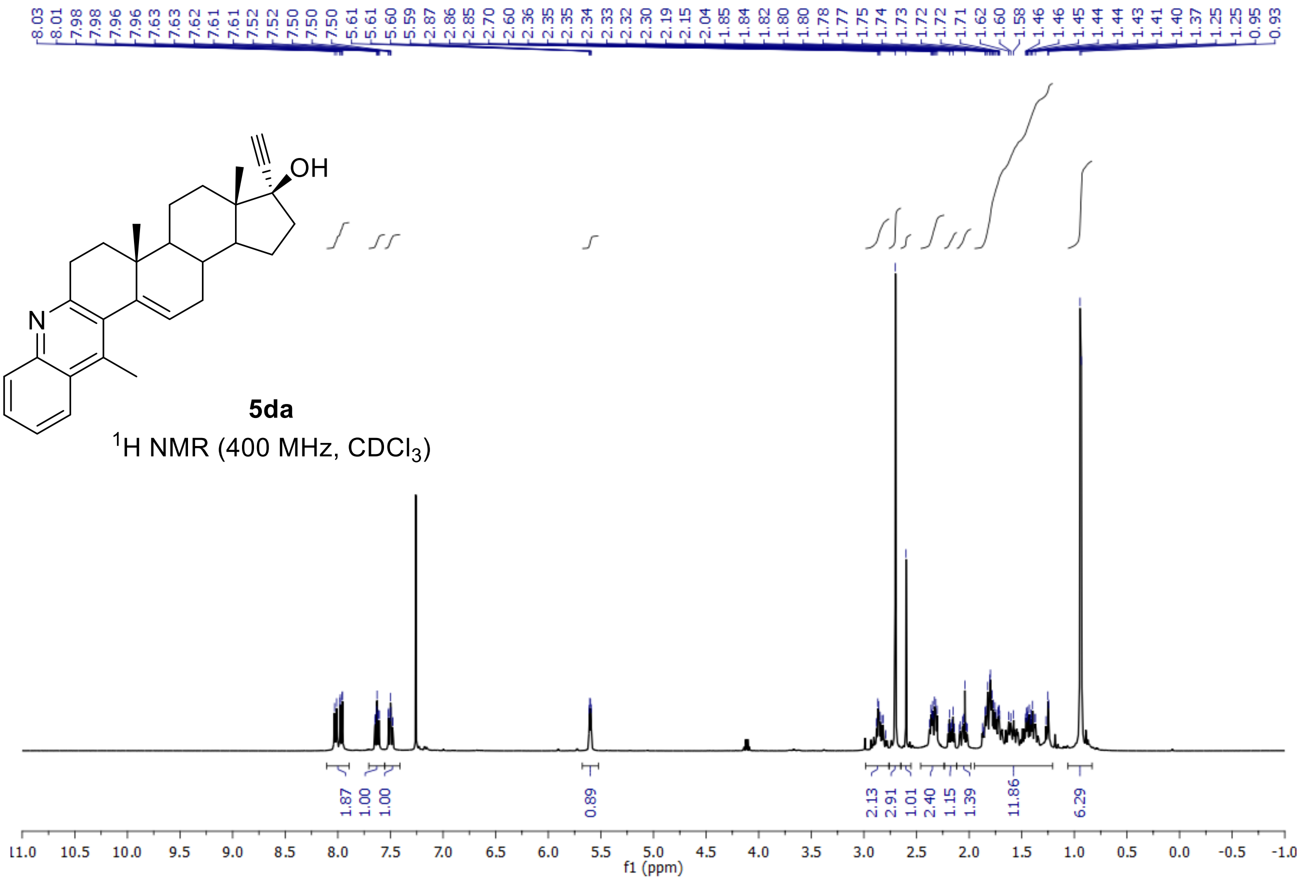
8.03 8.01 7.98 7.98 7.96 7.96 7.63 7.63 7.62 7.61 7.61 7.52 7.52 7.50 7.50 7.50 5.61 5.61 5.60 5.59 2.87 2.86 2.85 2.70 2.60 2.36 2.35 2.35 2.34 2.33 2.32 2.30 2.19 2.15 2.04 1.85 1.84 1.82 1.80 1.80 1.78 1.77 1.75 1.74 1.73 1.72 1.72 1.71 1.62 1.60 1.58 1.46 1.45 1.44 1.44 1.43 1.41 1.40 1.37 1.25 1.25 0.95 0.93
OH
N
5da
¹H NMR (400 MHz, CDCl₃)
1.87 1.00 1.00 0.89 2.13 2.91 1.01 2.40 1.15 1.39 11.86 6.29
f1 (ppm)

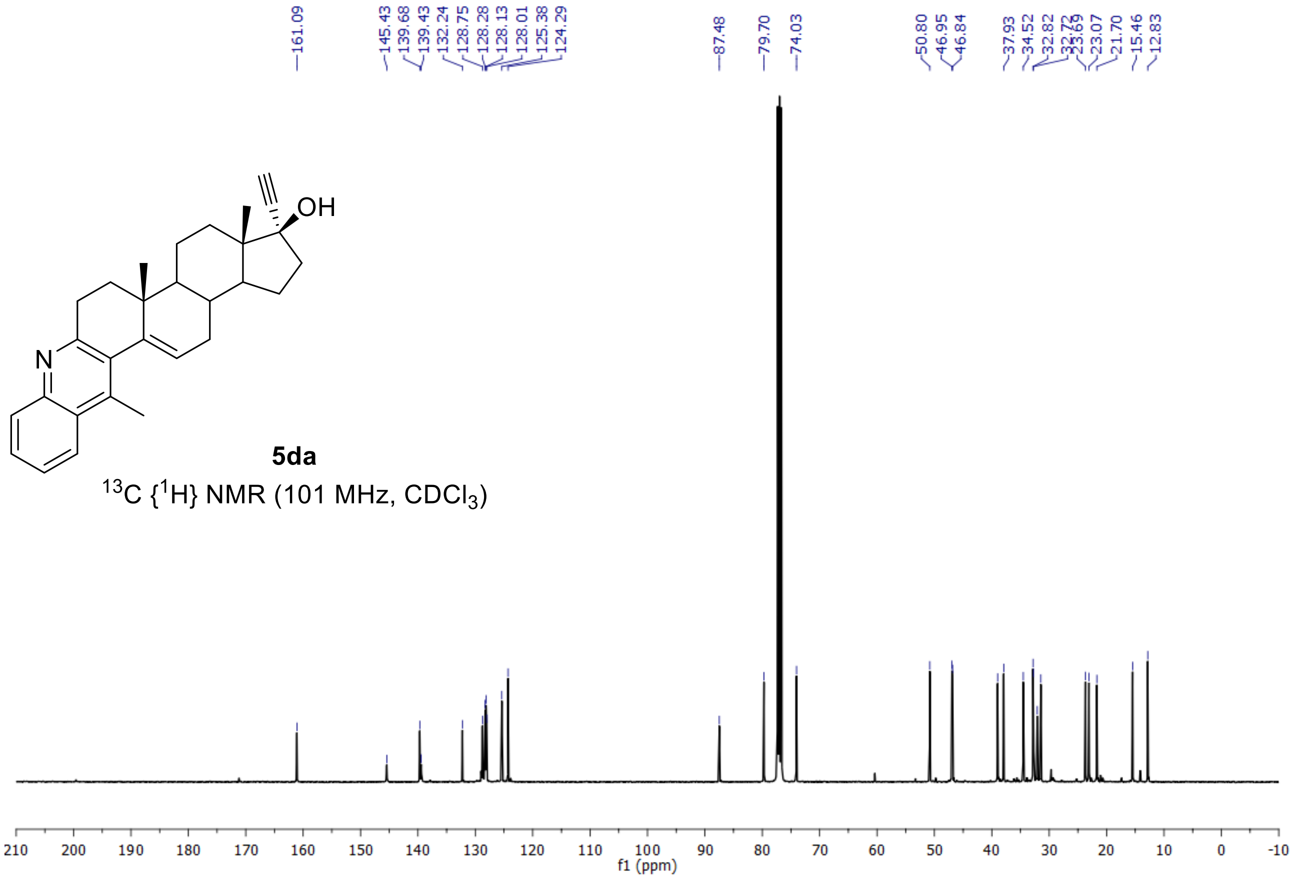
161.09 145.43 139.68 139.43 132.24 128.75 128.28 128.13 128.01 125.38 124.29 87.48 79.70 74.03 50.80 46.95 46.84 37.93 34.52 32.82 32.72 32.69 23.07 21.70 15.46 12.83
OH
N
5da
¹³C {¹H} NMR (101 MHz, CDCl₃)
f1 (ppm)

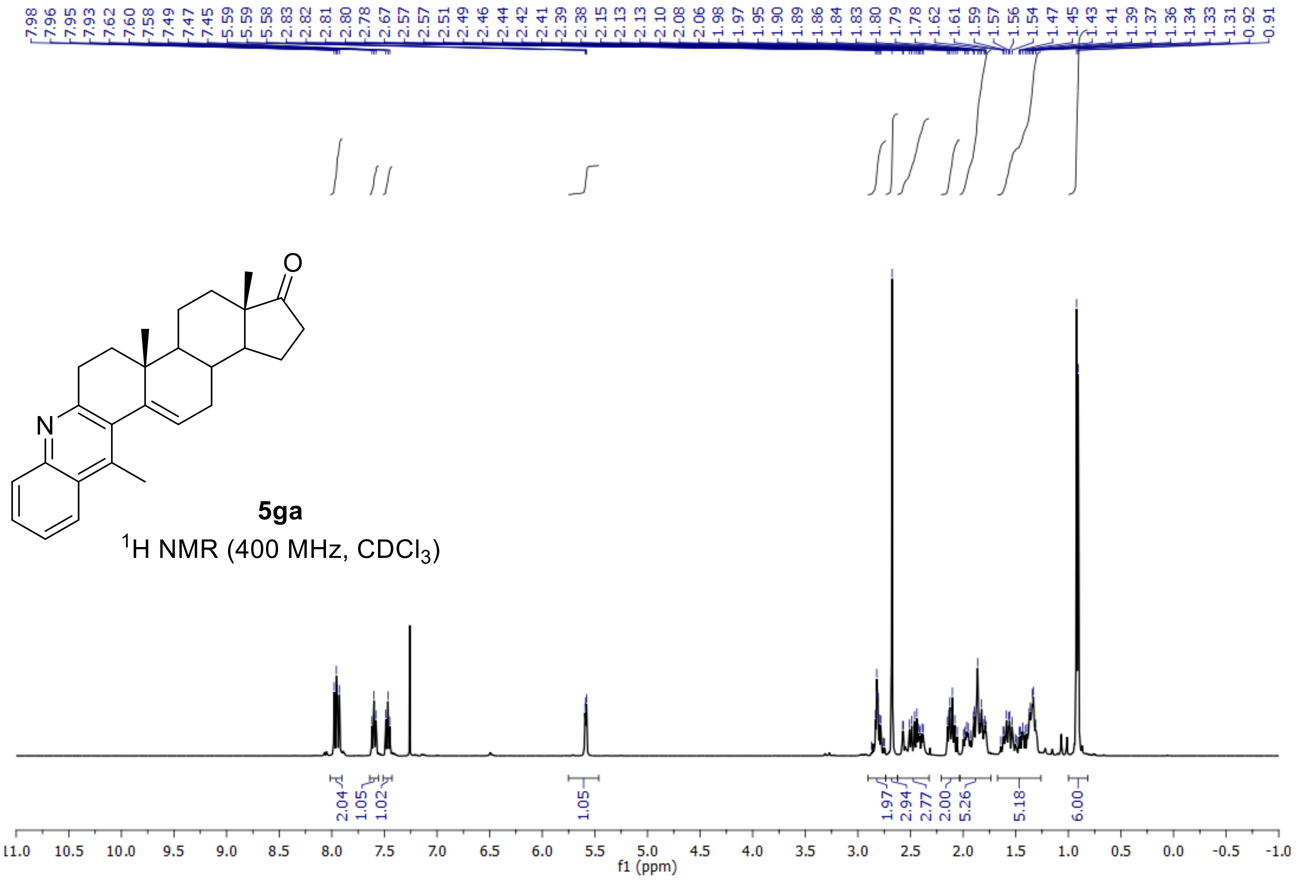
7.98 7.96 7.95 7.93 7.62 7.60 7.58 7.49 7.47 7.45 5.59 5.59 5.58 2.83 2.82 2.81 2.80 2.78 2.67 2.57 2.57 2.51 2.49 2.46 2.44 2.42 2.41 2.39 2.38 2.15 2.13 2.13 2.10 2.08 2.06 1.98 1.97 1.95 1.90 1.89 1.86 1.84 1.83 1.80 1.79 1.78 1.62 1.61 1.59 1.57 1.56 1.54 1.47 1.45 1.43 1.41 1.39 1.37 1.36 1.34 1.33 1.31 0.92 0.91
O
N
5ga
1H NMR (400 MHz, CDCl3)
2.04 1.05 1.02 1.05 1.97 2.94 2.77 2.00 5.26 5.18 6.00
11.0 10.5 10.0 9.5 9.0 8.5 8.0 7.5 7.0 6.5 6.0 5.5 5.0 4.5 4.0 3.5 3.0 2.5 2.0 1.5 1.0 0.5 0.0 -0.5 -1.0
f1 (ppm)

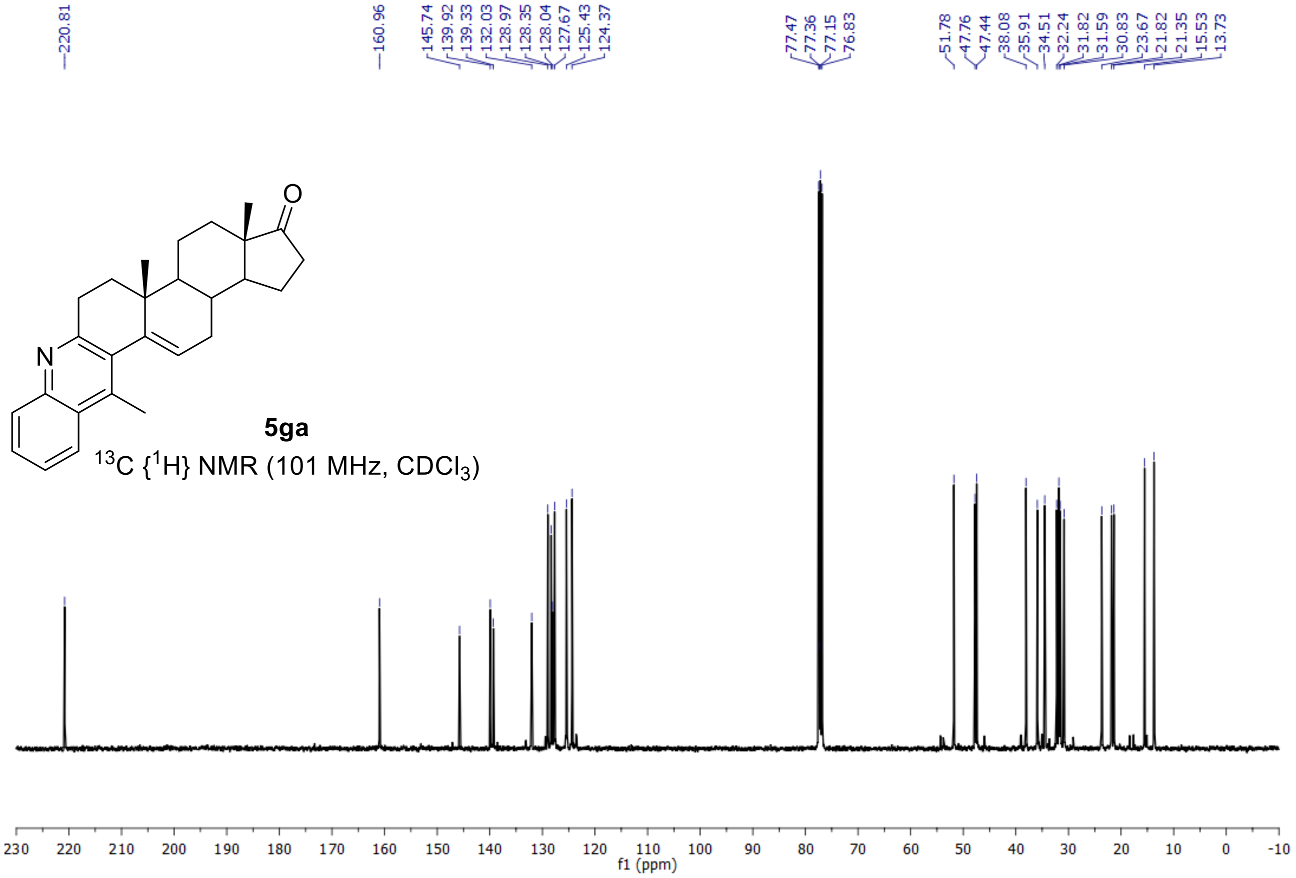
220.81 160.96 145.74 139.92 139.33 132.03 128.97 128.35 128.04 127.67 125.43 124.37 77.47 77.36 77.15 76.83 51.78 47.76 47.44 38.08 35.91 34.51 32.24 31.82 31.59 30.83 23.67 21.82 21.35 15.53 13.73
O
N
5ga
13C {1H} NMR (101 MHz, CDCl3)
230 220 210 200 190 180 170 160 150 140 130 120 110 100 90 80 70 60 50 40 30 20 10 0 -10
f1 (ppm)

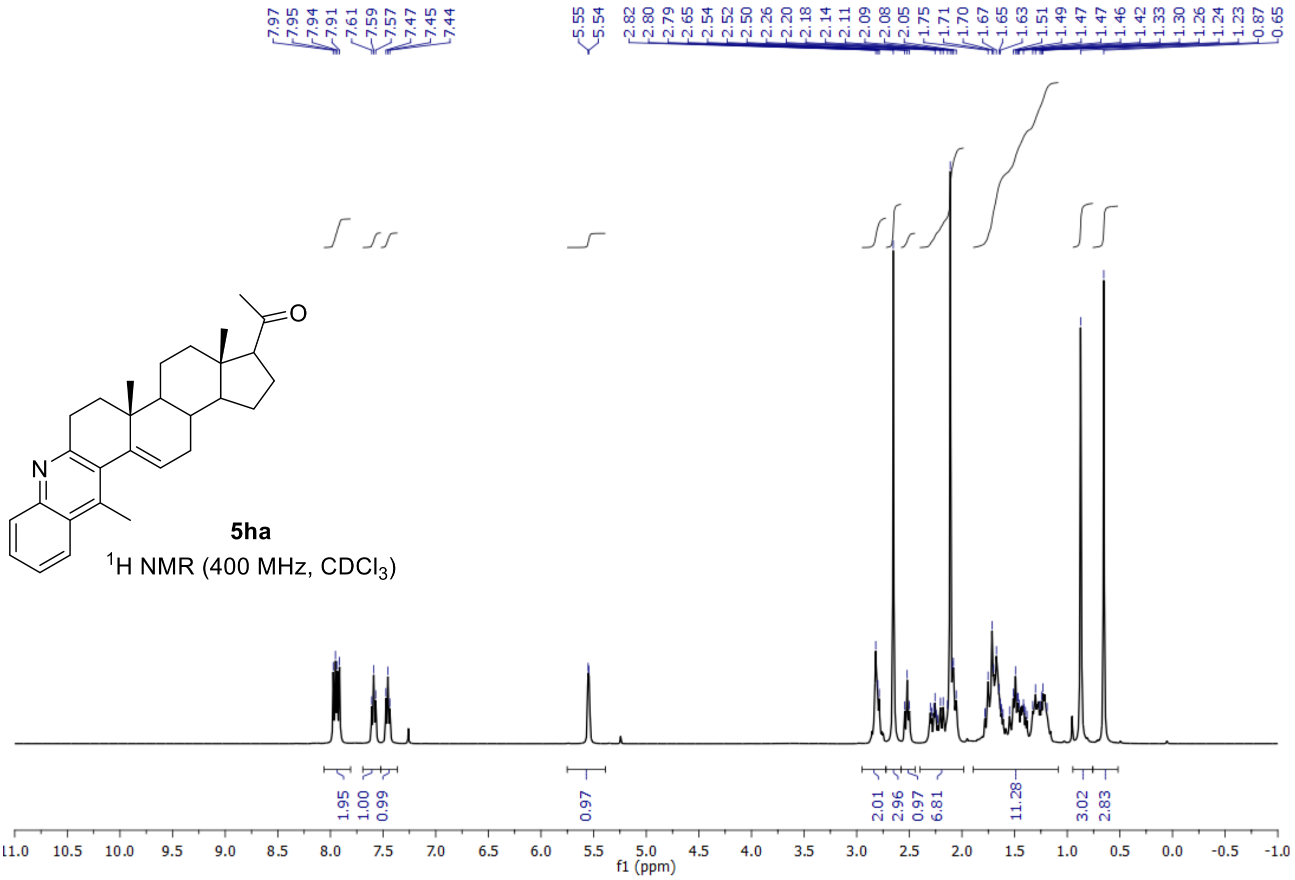

5ha

^{1}H NMR (400 MHz, $CDCl_3$)

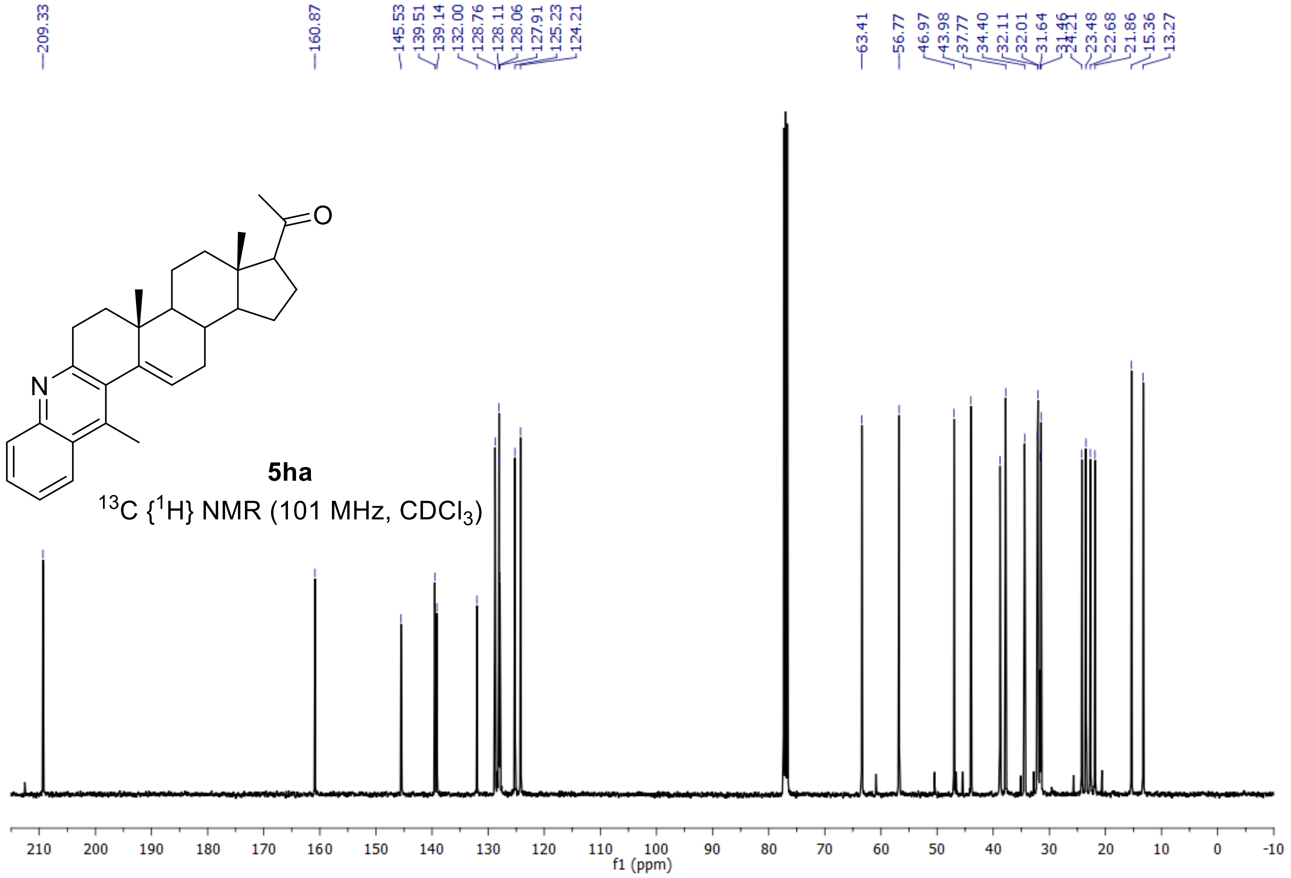

5ha

$^{13}C\ \{^{1}H\}$ NMR (101 MHz, $CDCl_3$)

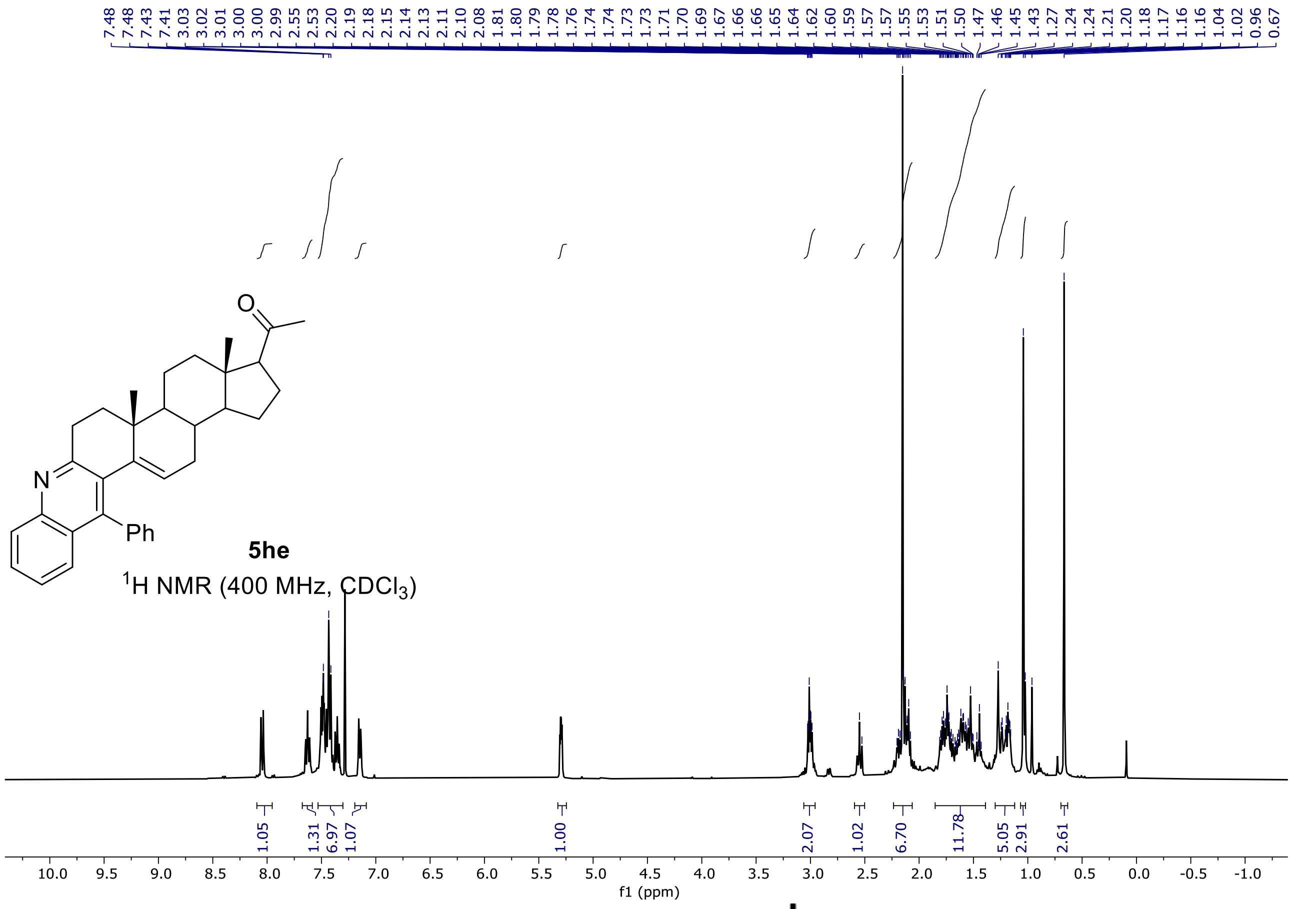
7.48 7.48 7.43 7.41 3.03 3.02 3.01 3.00 3.00 2.99 2.55 2.53 2.20 2.19 2.18 2.15 2.14 2.13 2.11 2.10 2.08 1.81 1.80 1.79 1.78 1.76 1.74 1.74 1.73 1.73 1.71 1.70 1.69 1.67 1.66 1.65 1.64 1.62 1.60 1.59 1.57 1.57 1.55 1.53 1.51 1.50 1.47 1.46 1.45 1.43 1.27 1.24 1.24 1.21 1.20 1.18 1.17 1.16 1.16 1.04 1.02 0.96 0.67
O
N
Ph
5he
1H NMR (400 MHz, CDCl3)
1.05 1.31 6.97 1.07 1.00 2.07 1.02 6.70 11.78 5.05 2.91 2.61
f1 (ppm)

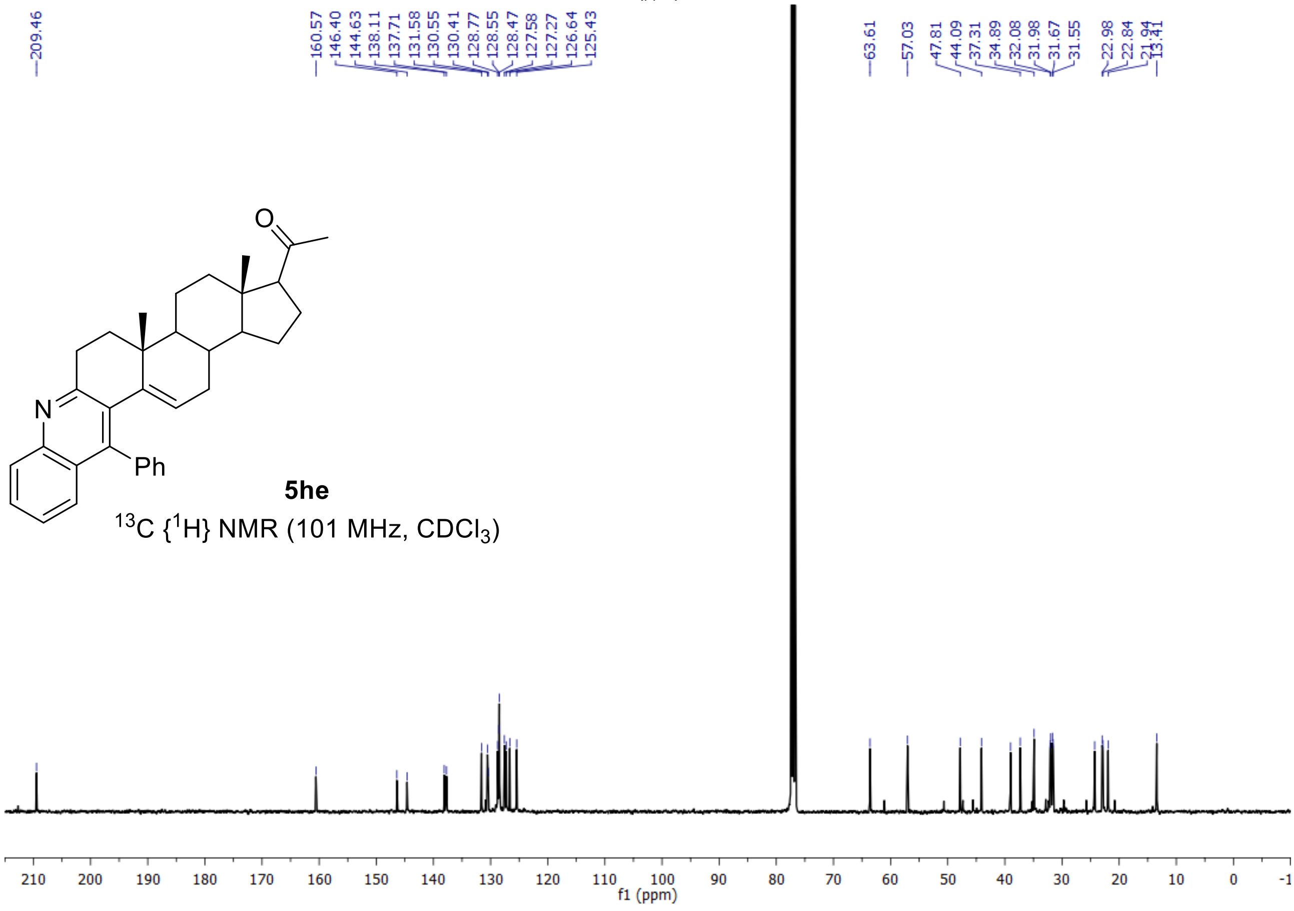
209.46
160.57 146.40 144.63 138.11 137.71 131.58 130.55 130.41 128.77 128.55 128.47 127.58 127.27 126.64 125.43
63.61 57.03 47.81 44.09 37.31 34.89 32.08 31.98 31.67 31.55 22.98 22.84 21.94 13.41
O
N
Ph
5he
13C {1H} NMR (101 MHz, CDCl3)
f1 (ppm)

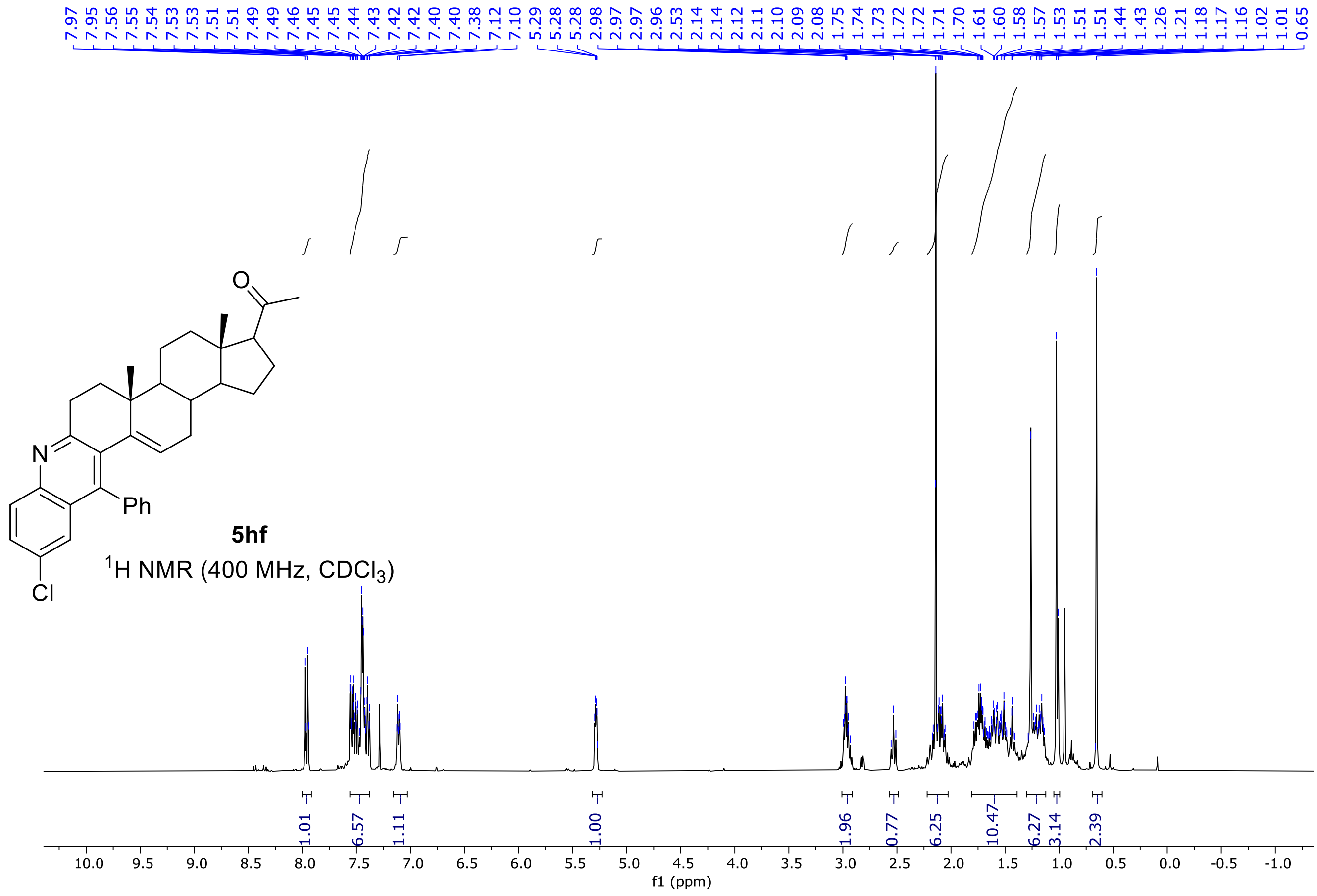

5hf

^{1}H NMR (400 MHz, $CDCl_3$)

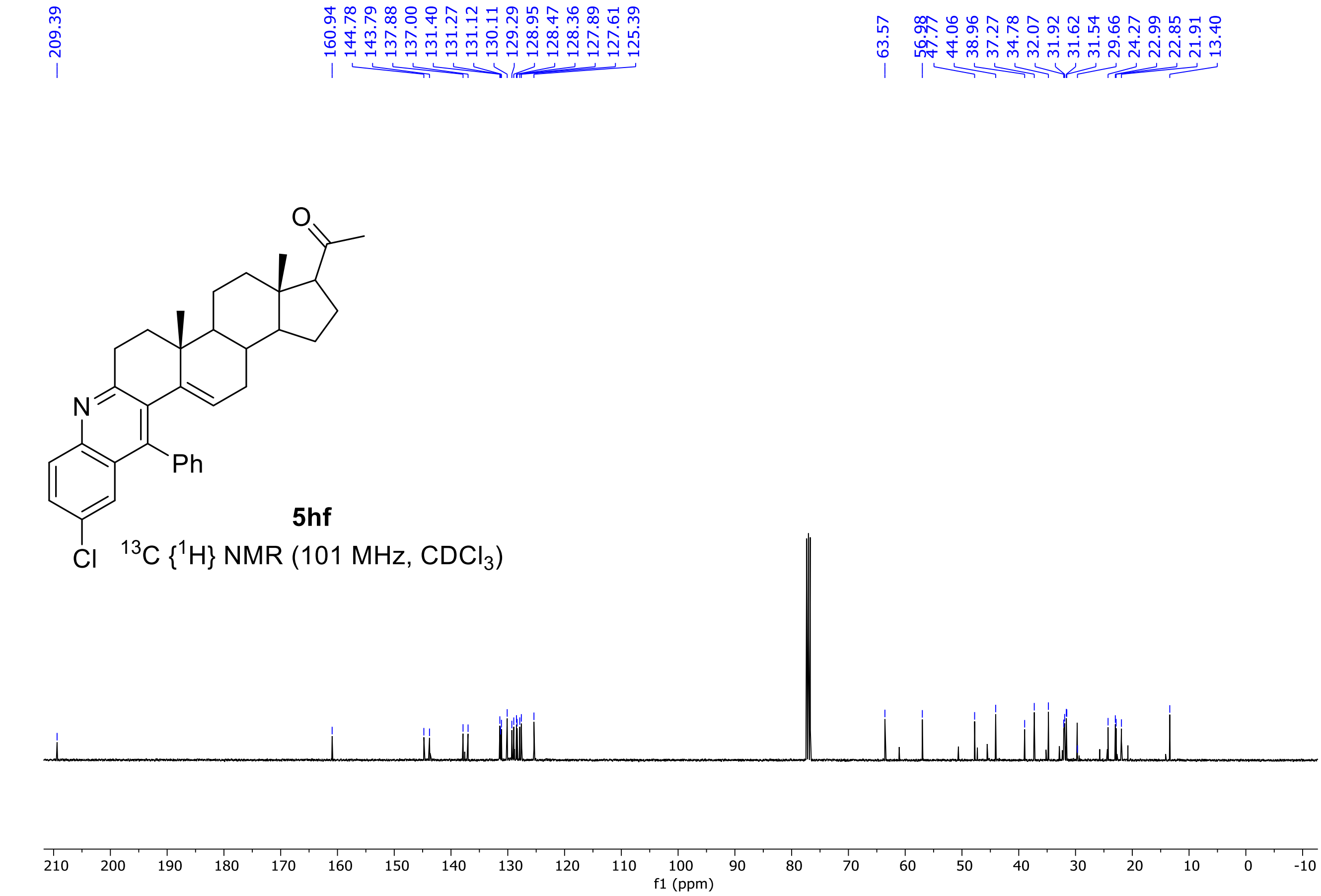

5hf

^{13}C {^{1}H} NMR (101 MHz, $CDCl_3$)

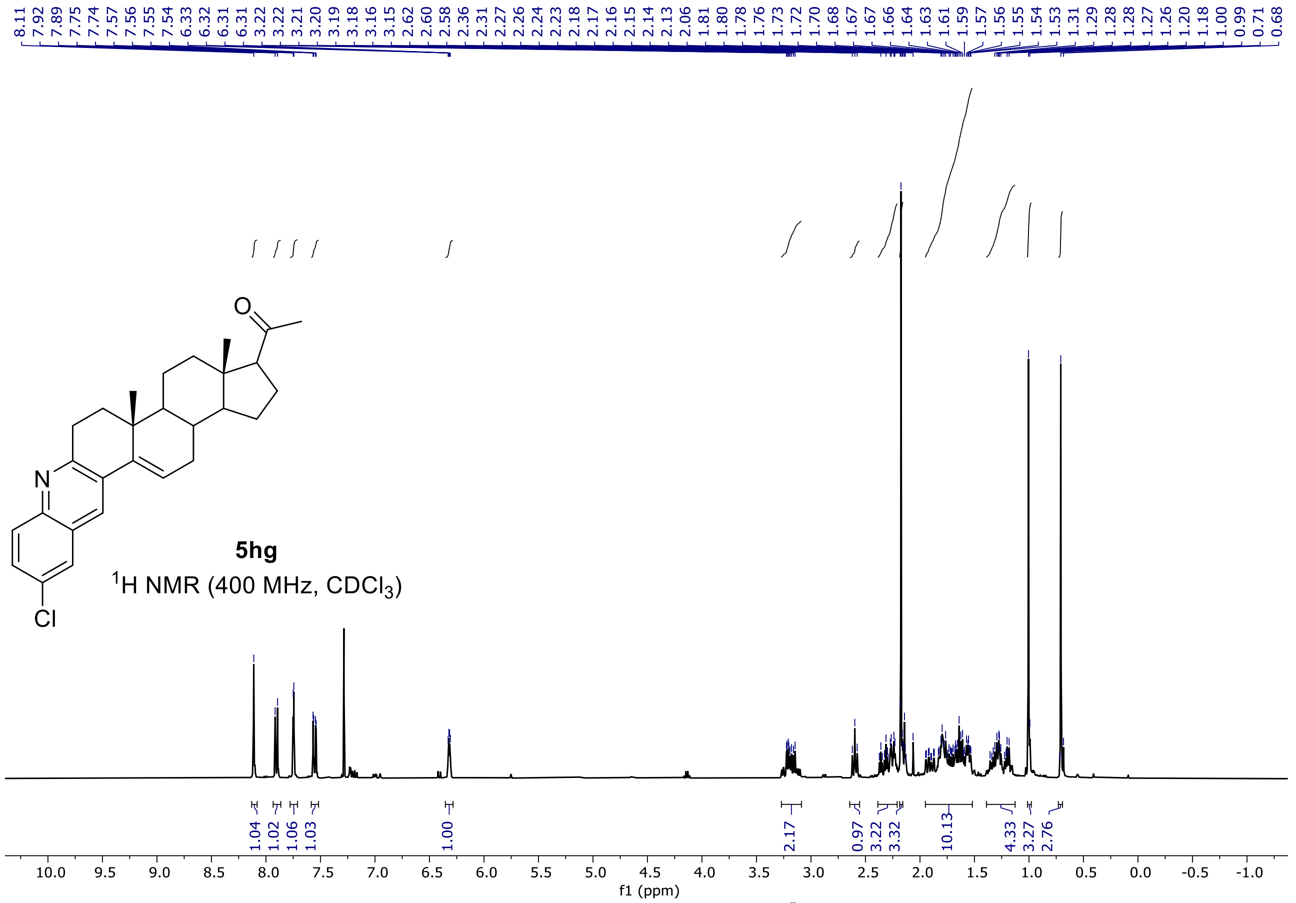
8.11 7.92 7.89 7.75 7.74 7.57 7.56 7.55 7.54 6.33 6.32 6.31 6.31 3.22 3.22 3.21 3.20 3.19 3.18 3.16 3.15 2.62 2.60 2.58 2.36 2.31 2.27 2.26 2.24 2.23 2.18 2.17 2.16 2.15 2.14 2.13 2.06 1.81 1.80 1.78 1.76 1.73 1.72 1.70 1.68 1.67 1.67 1.66 1.64 1.63 1.61 1.59 1.57 1.56 1.55 1.54 1.53 1.31 1.29 1.28 1.28 1.27 1.26 1.20 1.18 1.00 0.99 0.71 0.68
O
N
Cl
5hg
1H NMR (400 MHz, CDCl3)
1.04 1.02 1.06 1.03 1.00 2.17 0.97 3.22 3.32 10.13 4.33 3.27 2.76
10.0 9.5 9.0 8.5 8.0 7.5 7.0 6.5 6.0 5.5 5.0 4.5 4.0 3.5 3.0 2.5 2.0 1.5 1.0 0.5 0.0 -0.5 -1.0
f1 (ppm)

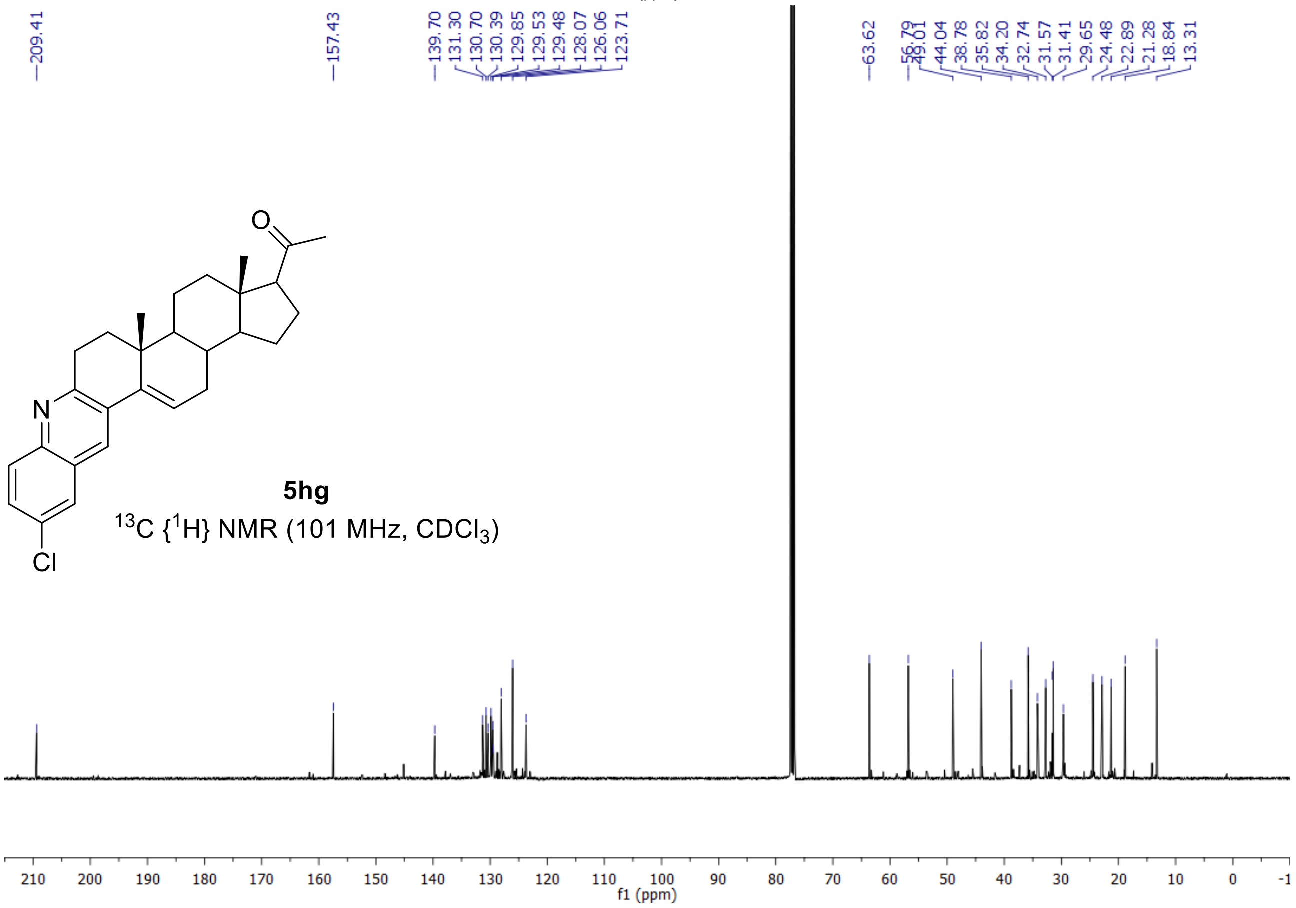
209.41 157.43 139.70 131.30 130.70 130.39 129.85 129.53 129.48 128.07 126.06 123.71 63.62 56.79 49.01 44.04 38.78 35.82 34.20 32.74 31.57 31.41 29.65 24.48 22.89 21.28 18.84 13.31
O
N
Cl
5hg
13C {1H} NMR (101 MHz, CDCl3)
210 200 190 180 170 160 150 140 130 120 110 100 90 80 70 60 50 40 30 20 10 0 -1
f1 (ppm)

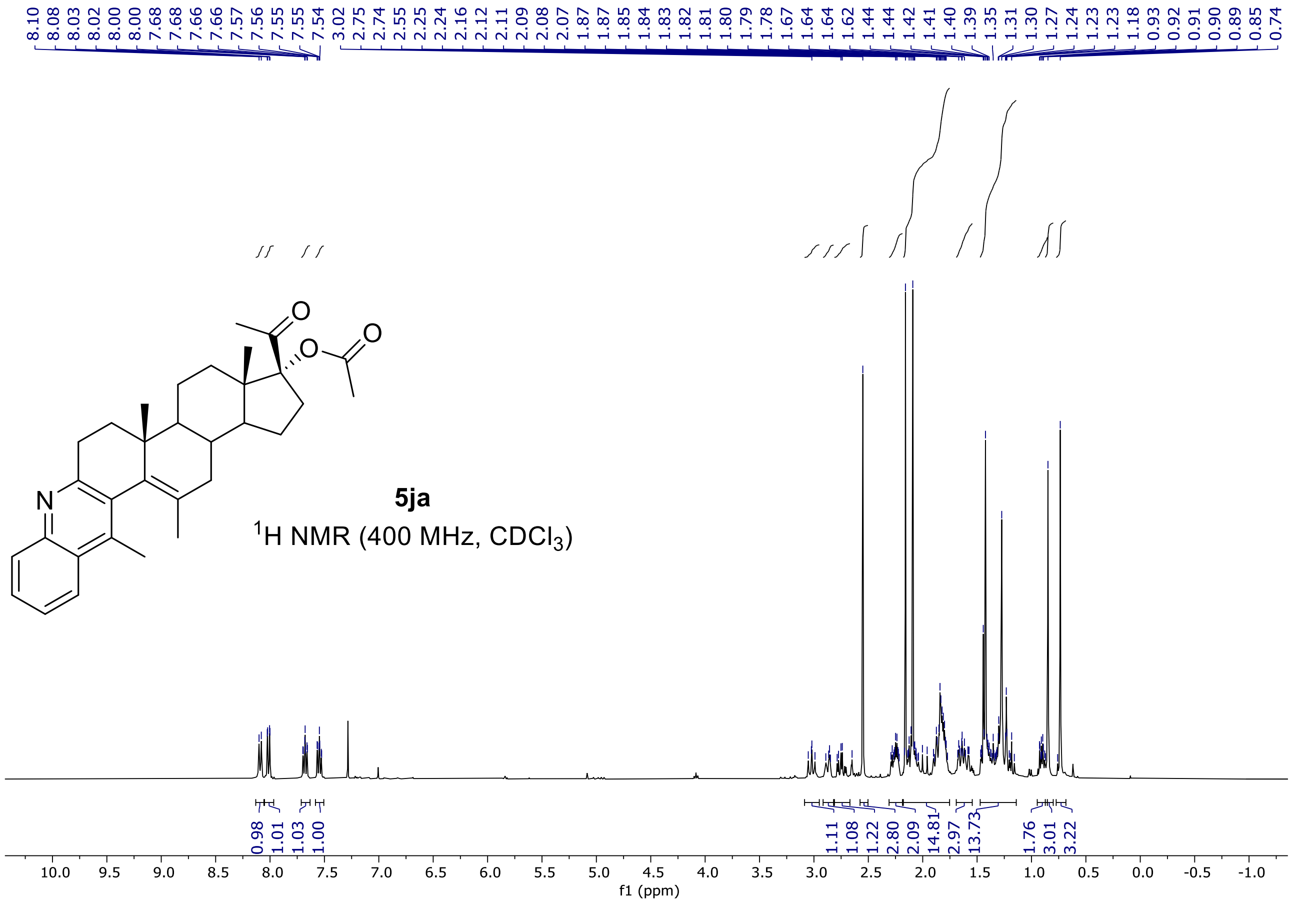
8.10 8.08 8.03 8.02 8.00 8.00 7.68 7.68 7.66 7.66 7.57 7.56 7.55 7.55 7.54 3.02 2.75 2.74 2.55 2.25 2.24 2.16 2.12 2.11 2.09 2.08 2.07 1.87 1.87 1.85 1.84 1.83 1.82 1.81 1.80 1.79 1.78 1.67 1.64 1.62 1.44 1.44 1.42 1.41 1.40 1.39 1.35 1.31 1.30 1.27 1.24 1.23 1.23 1.18 0.93 0.92 0.91 0.90 0.89 0.85 0.74
N
O
O
O
5ja
^{1}H NMR (400 MHz, CDCl$_3$)
0.98 1.01 1.03 1.00 1.11 1.08 1.22 2.80 2.09 14.81 2.97 13.73 1.76 3.01 3.22
10.0 9.5 9.0 8.5 8.0 7.5 7.0 6.5 6.0 5.5 5.0 4.5 4.0 3.5 3.0 2.5 2.0 1.5 1.0 0.5 0.0 -0.5 -1.0
f1 (ppm)

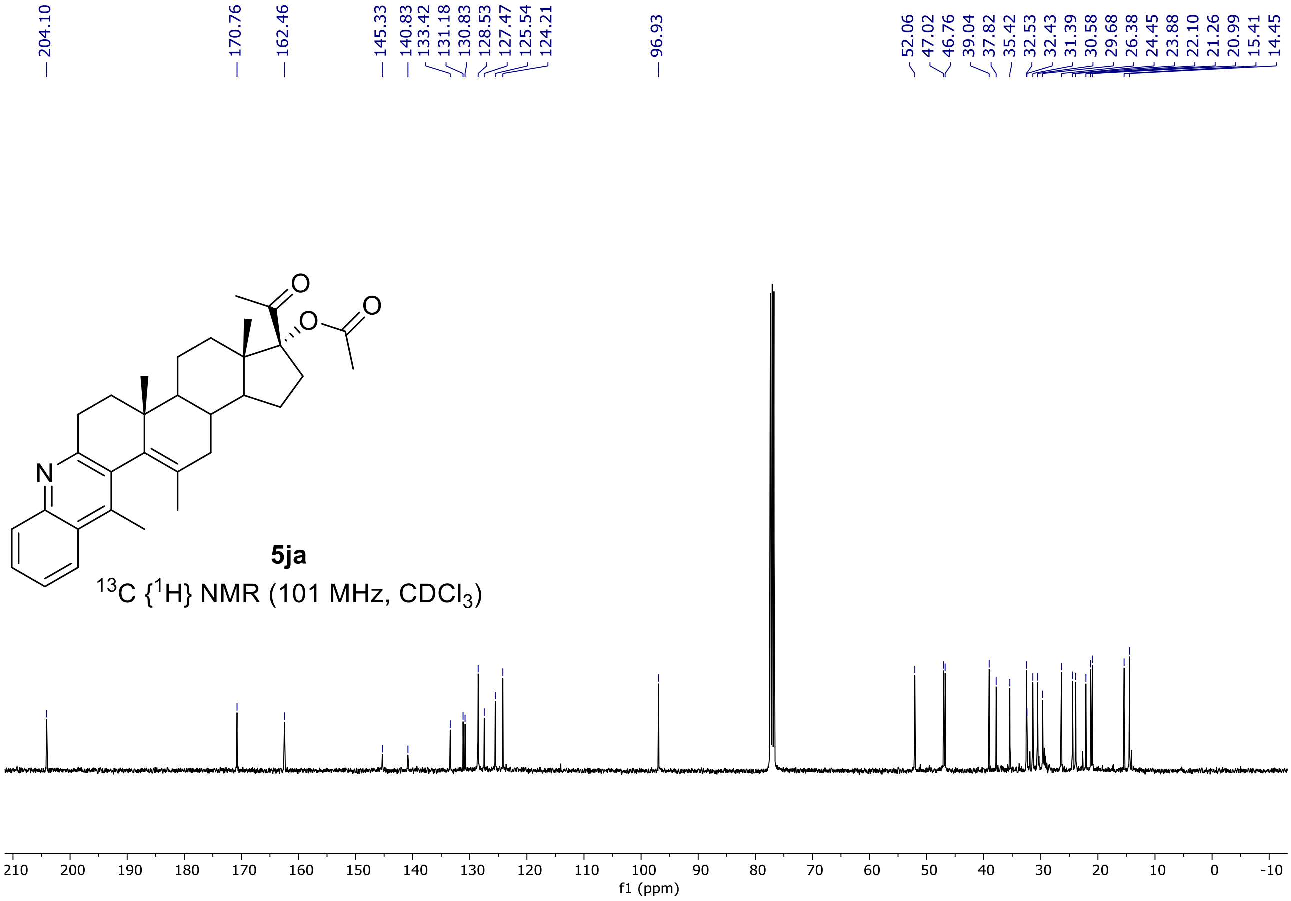
204.10 170.76 162.46 145.33 140.83 133.42 131.18 130.83 128.53 127.47 125.54 124.21 96.93 52.06 47.02 46.76 39.04 37.82 35.42 32.53 32.43 31.39 30.58 29.68 26.38 24.45 23.88 22.10 21.26 20.99 15.41 14.45
N
O
O
O
5ja
^{13}C {^{1}H} NMR (101 MHz, CDCl$_3$)
210 200 190 180 170 160 150 140 130 120 110 100 90 80 70 60 50 40 30 20 10 0 -10
f1 (ppm)

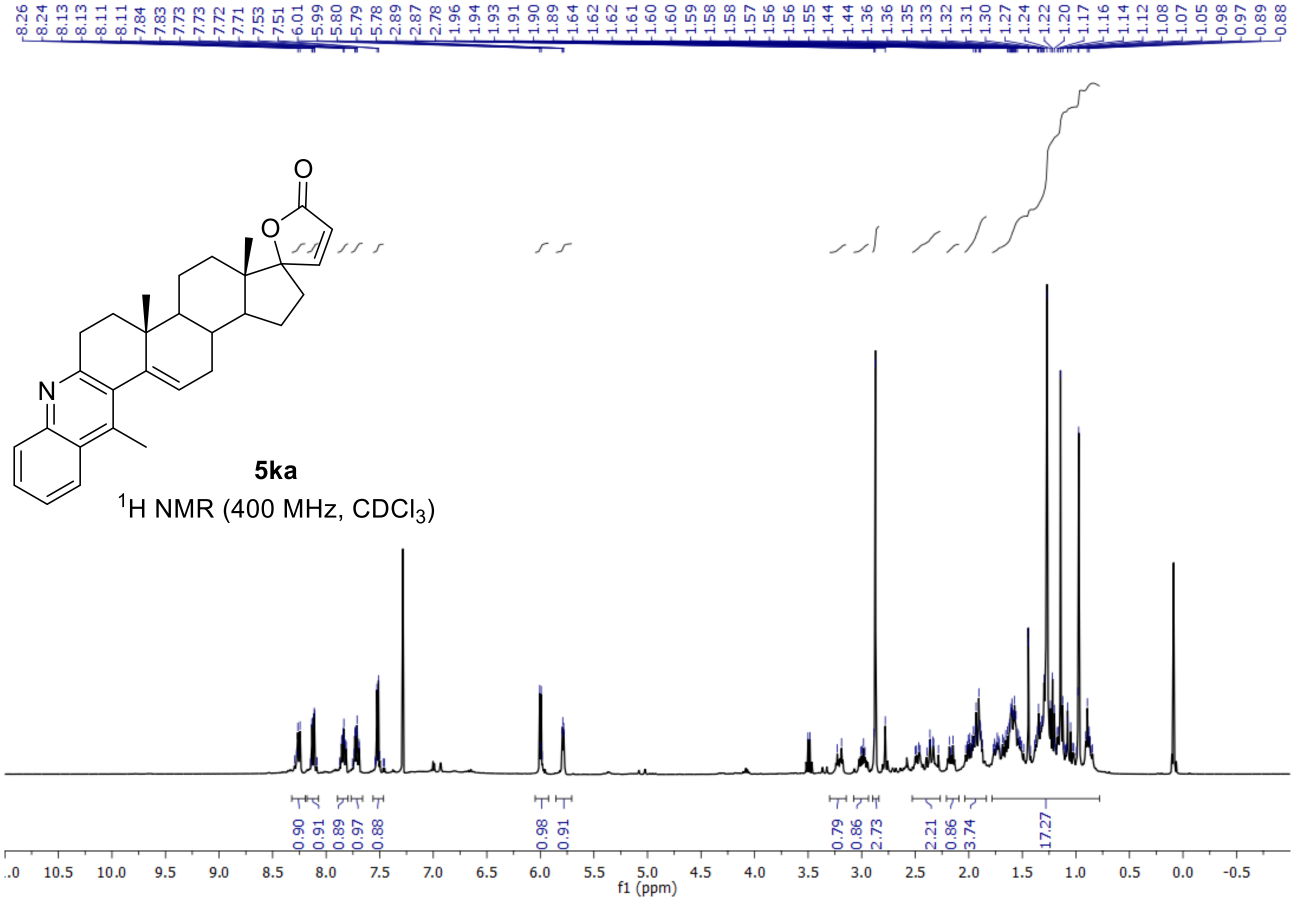
8.26 8.24 8.13 8.13 8.11 8.11 7.84 7.83 7.73 7.73 7.72 7.71 7.53 7.51 6.01 5.99 5.80 5.79 5.78 2.89 2.87 2.78 1.96 1.94 1.93 1.91 1.90 1.89 1.64 1.62 1.62 1.61 1.60 1.59 1.58 1.58 1.57 1.56 1.56 1.55 1.44 1.44 1.36 1.36 1.35 1.33 1.32 1.31 1.30 1.27 1.24 1.22 1.20 1.17 1.16 1.14 1.12 1.08 1.07 1.05 0.98 0.97 0.89 0.88
O
O
N
5ka
^{1}H NMR (400 MHz, CDCl$_3$)
0.90 0.91 0.89 0.97 0.88 0.98 0.91 0.79 0.86 2.73 2.21 0.86 3.74 17.27
.0 10.5 10.0 9.5 9.0 8.5 8.0 7.5 7.0 6.5 6.0 5.5 5.0 4.5 4.0 3.5 3.0 2.5 2.0 1.5 1.0 0.5 0.0 -0.5
f1 (ppm)

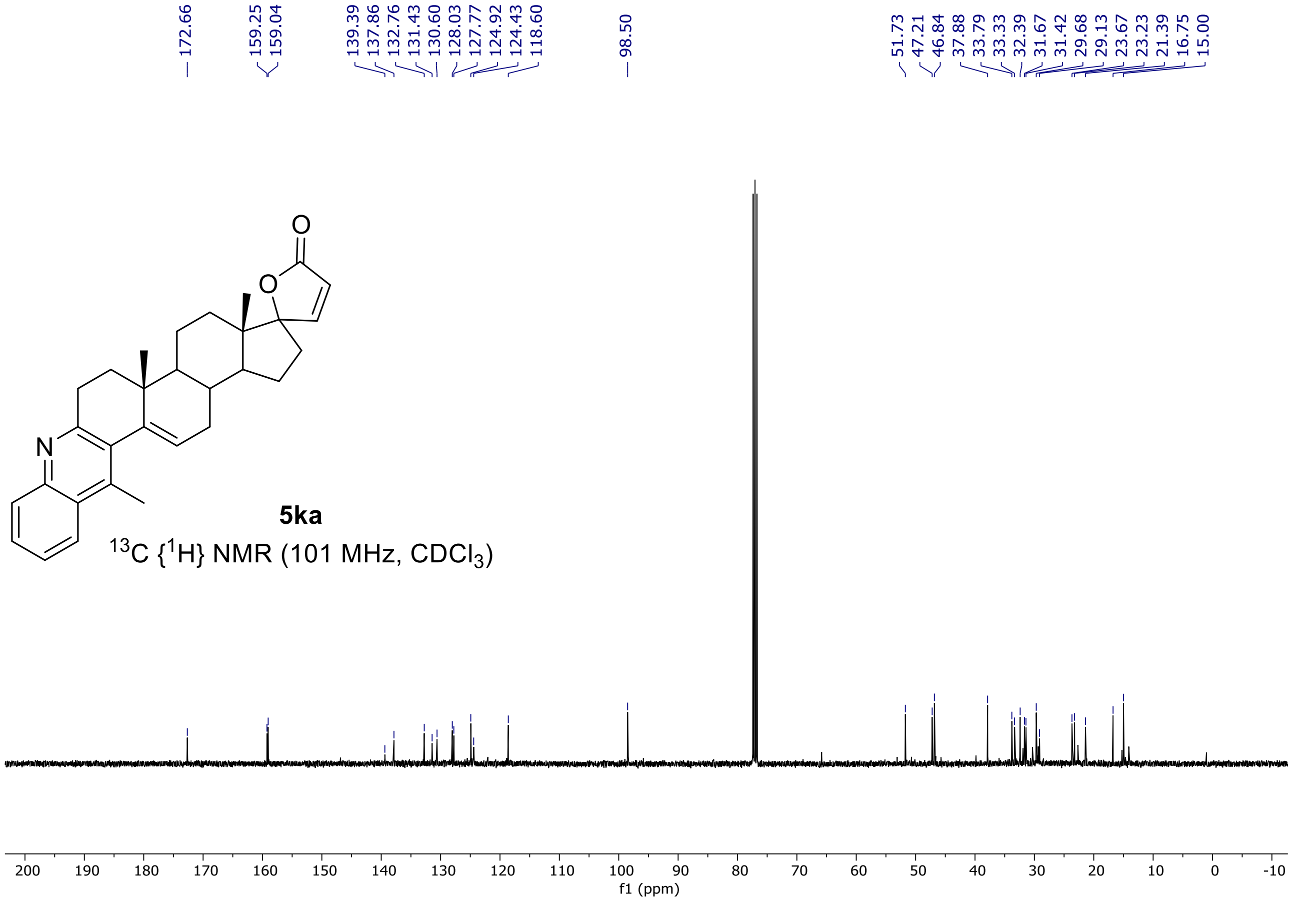
172.66 159.25 159.04 139.39 137.86 132.76 131.43 130.60 128.03 127.77 124.92 124.43 118.60 98.50 51.73 47.21 46.84 37.88 33.79 33.33 32.39 31.67 31.42 29.68 29.13 23.67 23.23 21.39 16.75 15.00
O
O
N
5ka
^{13}C {^{1}H} NMR (101 MHz, CDCl$_3$)
200 190 180 170 160 150 140 130 120 110 100 90 80 70 60 50 40 30 20 10 0 -10
f1 (ppm)

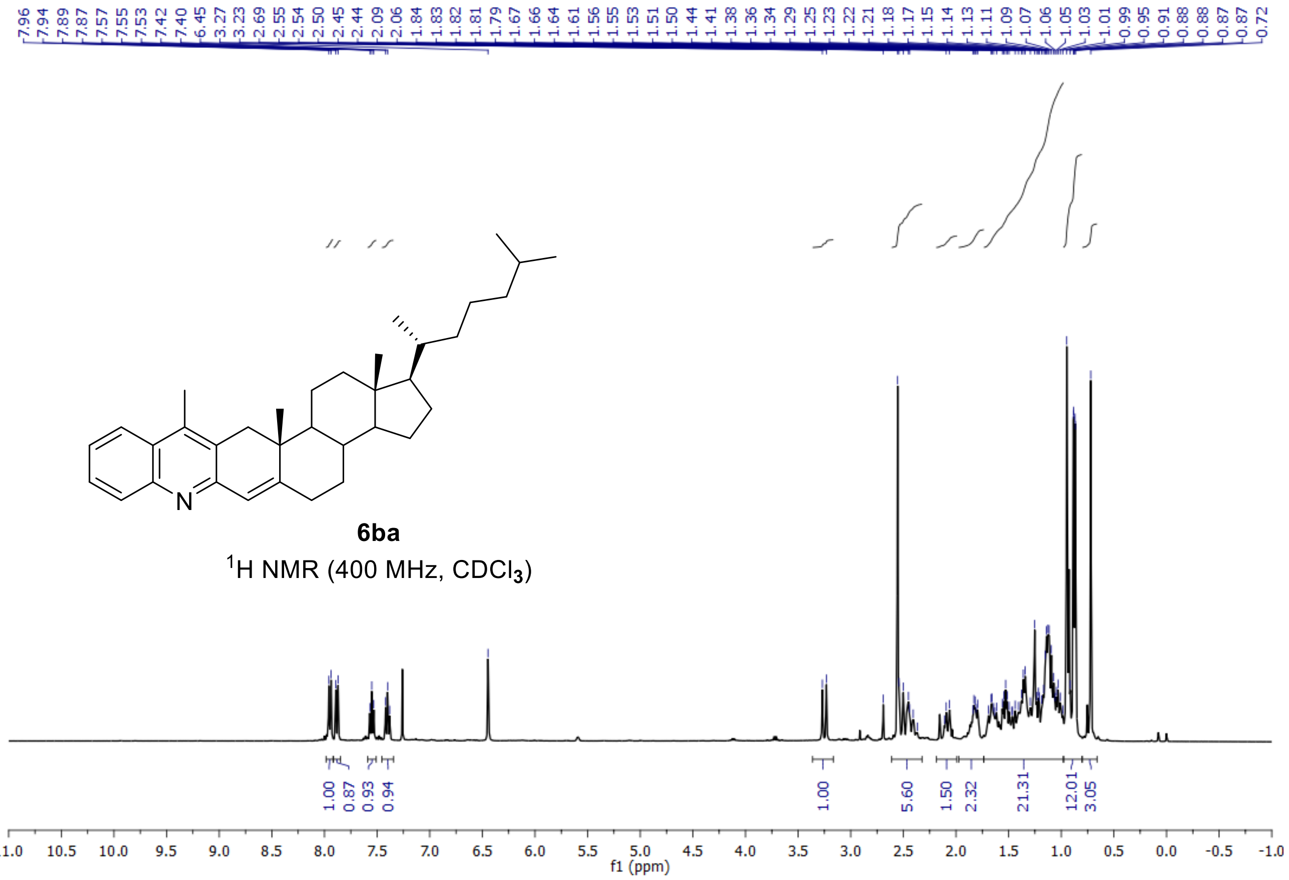
7.96 7.94 7.89 7.87 7.57 7.55 7.53 7.42 7.40 6.45 3.27 3.23 2.69 2.55 2.54 2.50 2.45 2.44 2.09 2.06 1.84 1.83 1.82 1.81 1.79 1.67 1.66 1.64 1.61 1.56 1.55 1.53 1.51 1.50 1.44 1.41 1.38 1.36 1.34 1.29 1.25 1.23 1.22 1.21 1.18 1.17 1.15 1.14 1.13 1.11 1.09 1.07 1.06 1.05 1.03 1.01 0.99 0.95 0.91 0.88 0.88 0.87 0.87 0.72
N
6ba
^{1}H NMR (400 MHz, $CDCl_3$)
1.00 0.87 0.93 0.94 1.00 5.60 1.50 2.32 21.31 12.01 3.05
f1 (ppm)

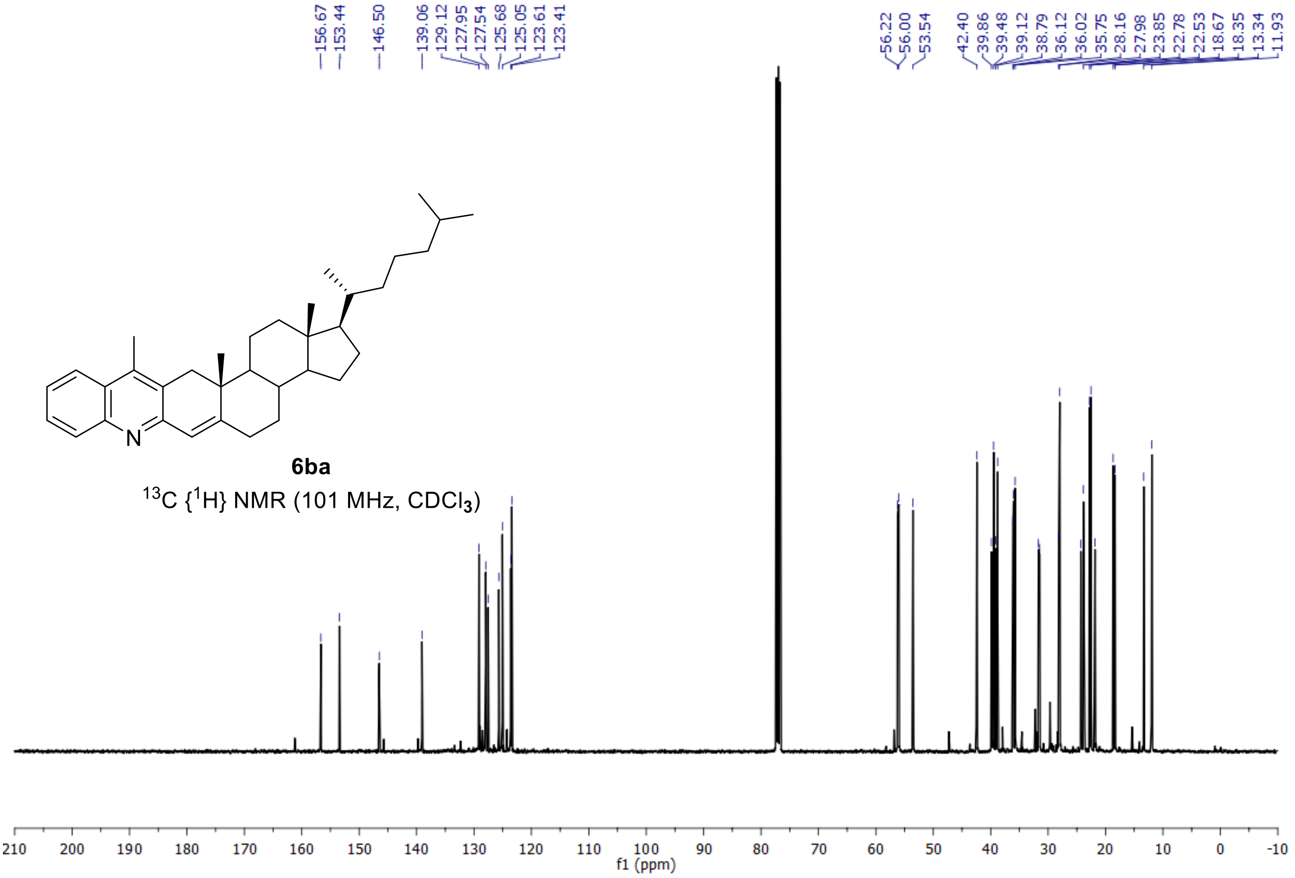
156.67 153.44 146.50 139.06 129.12 127.95 127.54 125.68 125.05 123.61 123.41
56.22 56.00 53.54 42.40 39.86 39.48 39.12 38.79 36.12 36.02 35.75 28.16 27.98 23.85 22.78 22.53 18.67 18.35 13.34 11.93
N
6ba
^{13}C {^{1}H} NMR (101 MHz, $CDCl_3$)
f1 (ppm)

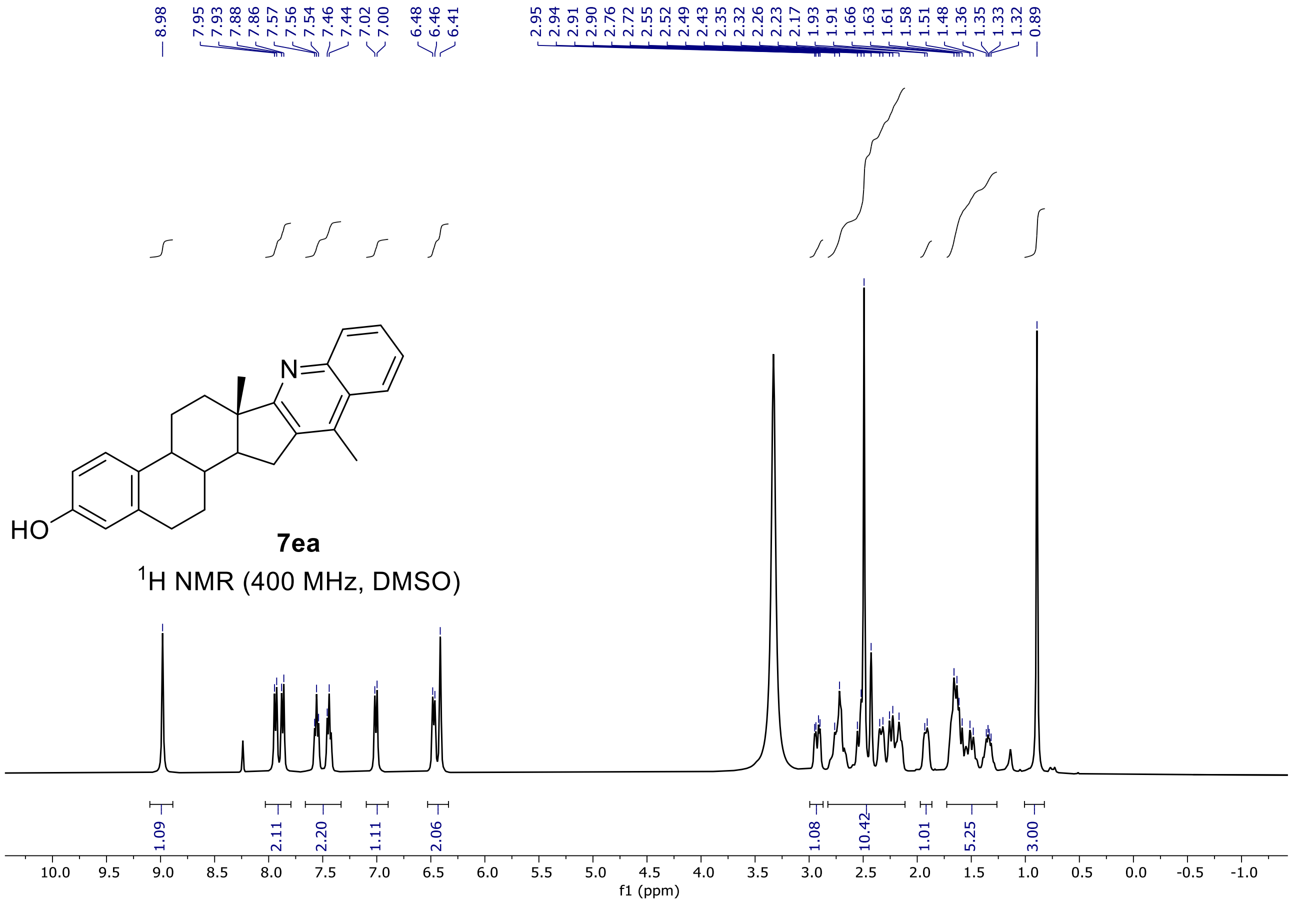
8.98
7.95
7.93
7.88
7.86
7.57
7.56
7.54
7.46
7.44
7.02
7.00
6.48
6.46
6.41
2.95
2.94
2.91
2.90
2.76
2.72
2.55
2.52
2.49
2.43
2.35
2.32
2.26
2.23
2.17
1.93
1.91
1.66
1.63
1.61
1.58
1.51
1.48
1.36
1.35
1.33
1.32
0.89
HO
N
7ea
¹H NMR (400 MHz, DMSO)
1.09
2.11
2.20
1.11
2.06
1.08
10.42
1.01
5.25
3.00
10.0 9.5 9.0 8.5 8.0 7.5 7.0 6.5 6.0 5.5 5.0 4.5 4.0 3.5 3.0 2.5 2.0 1.5 1.0 0.5 0.0 -0.5 -1.0
f1 (ppm)

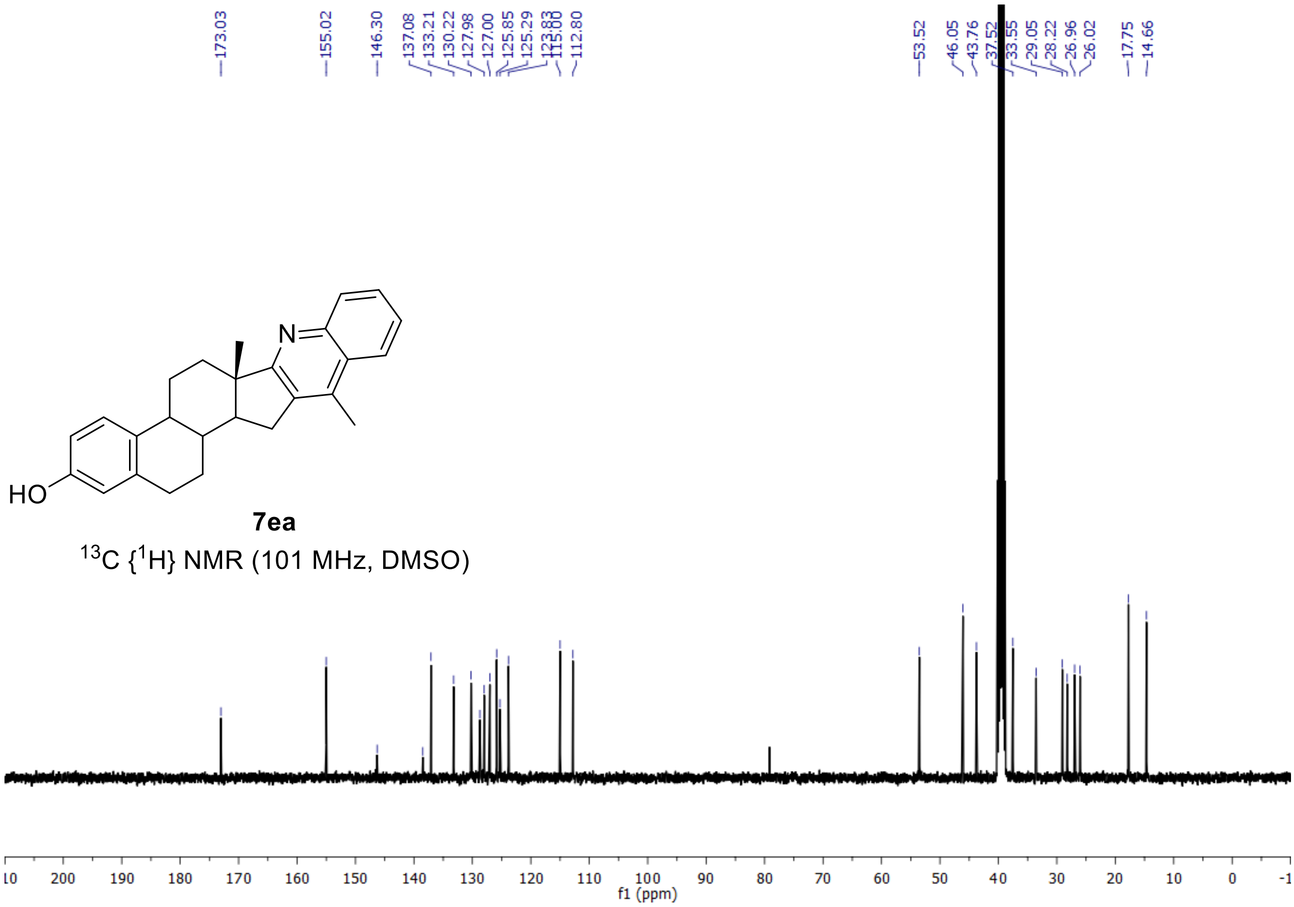
173.03
155.02
146.30
137.08
133.21
130.22
127.98
127.00
125.85
125.29
123.83
115.00
112.80
53.52
46.05
43.76
37.52
33.55
29.05
28.22
26.96
26.02
17.75
14.66
HO
N
7ea
¹³C {¹H} NMR (101 MHz, DMSO)
10 200 190 180 170 160 150 140 130 120 110 100 90 80 70 60 50 40 30 20 10 0 -1
f1 (ppm)

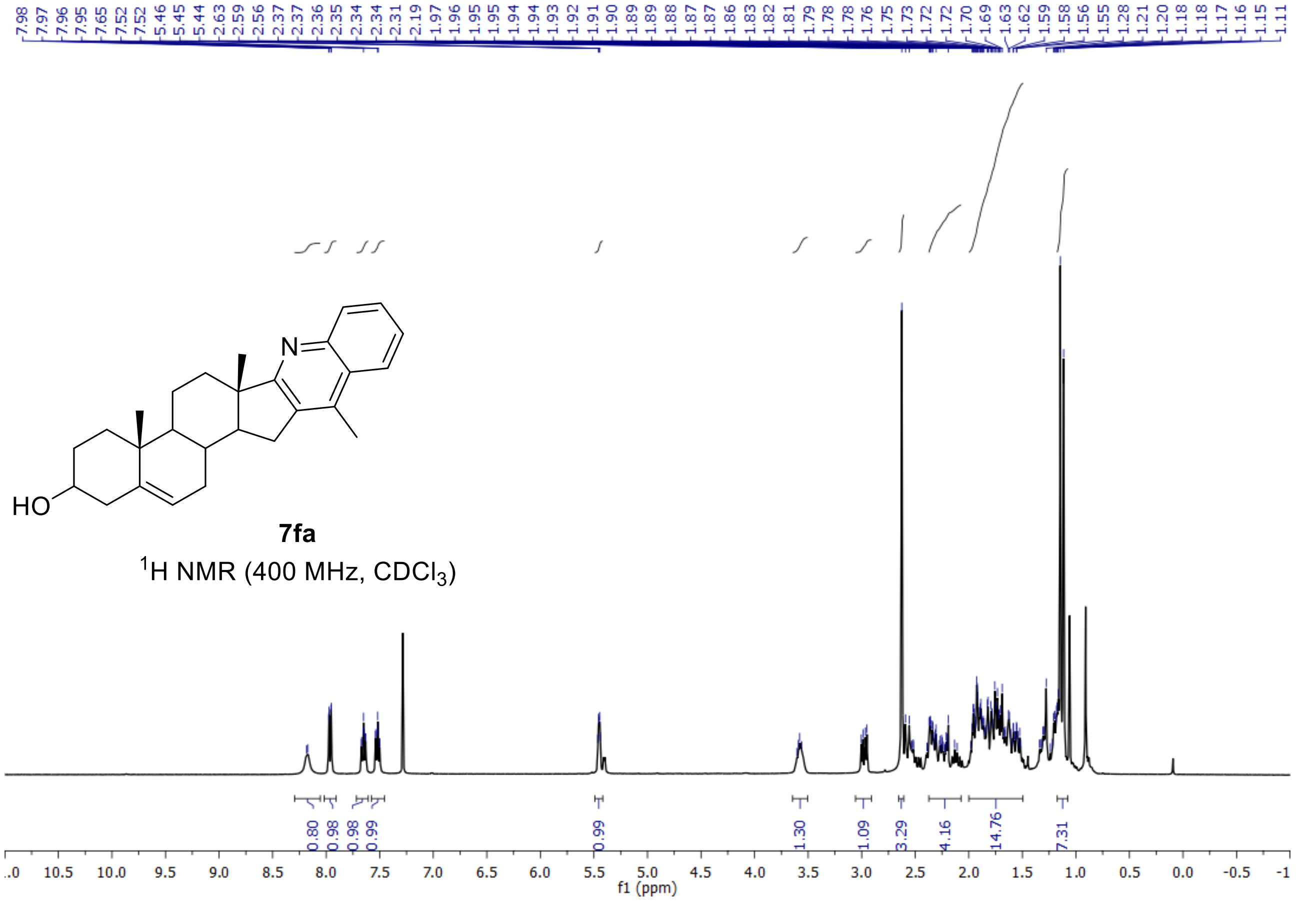

7fa

^{1}H NMR (400 MHz, $CDCl_3$)

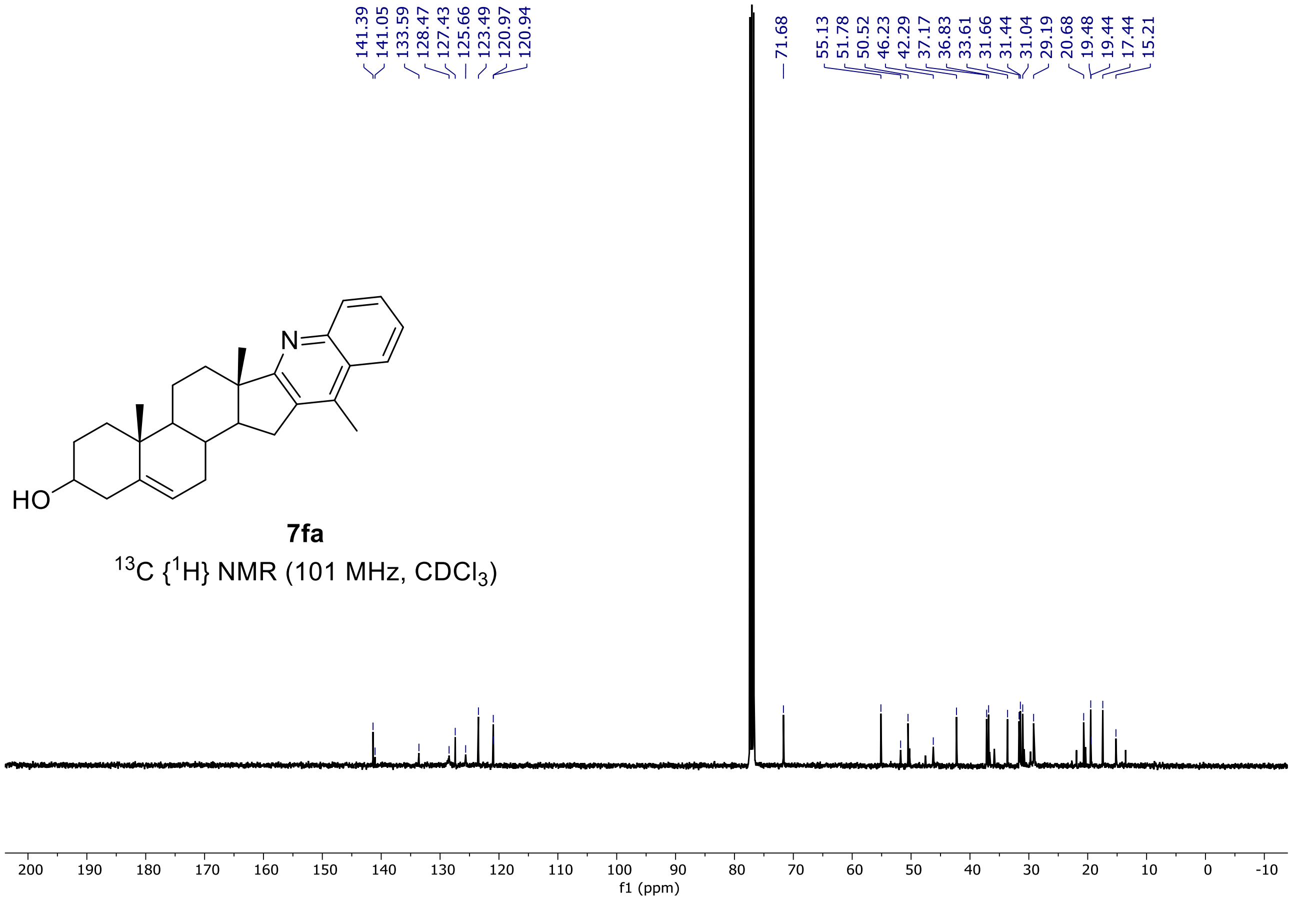

7fa

^{13}C {^{1}H} NMR (101 MHz, $CDCl_3$)

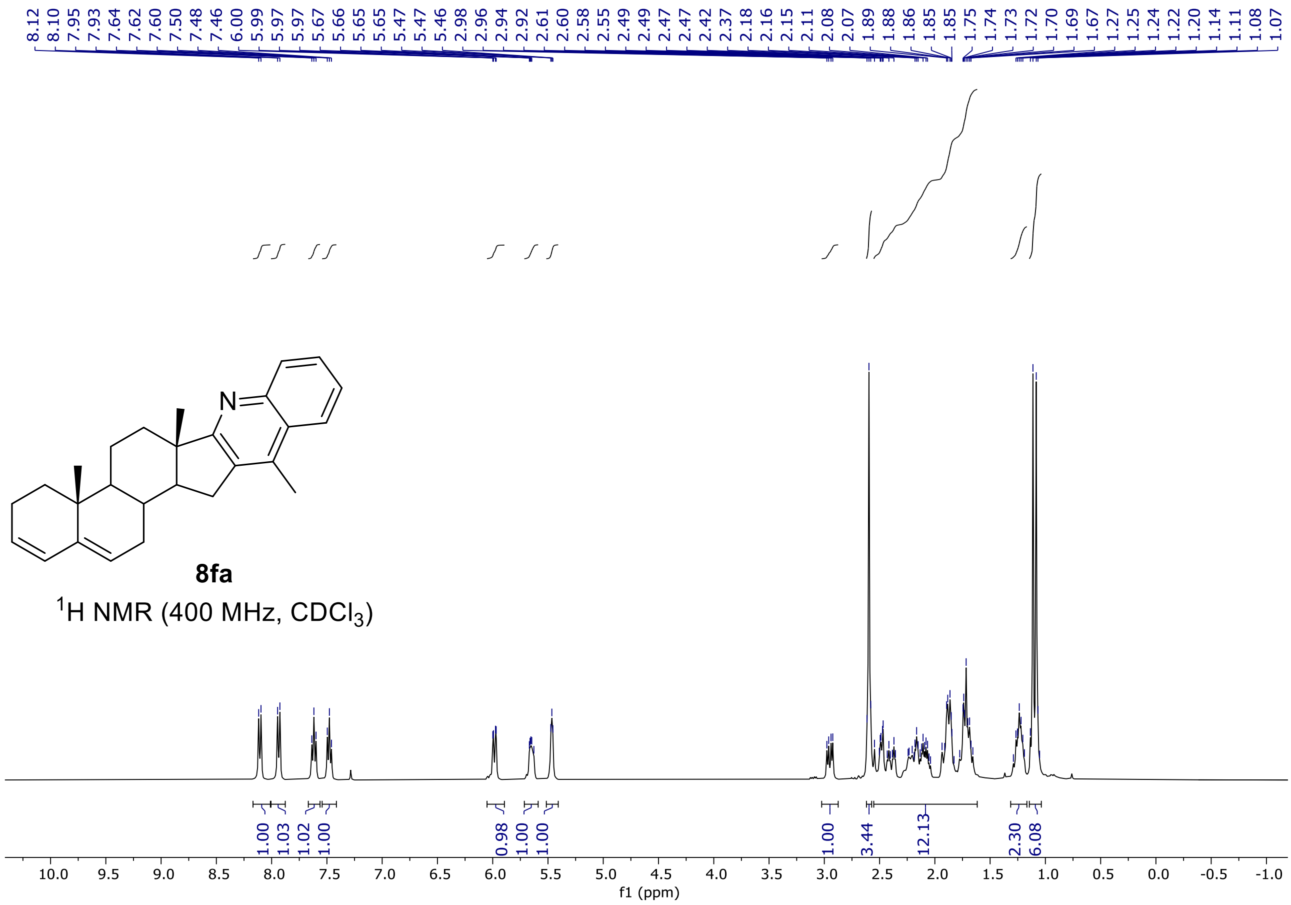
8.12 8.10 7.95 7.93 7.64 7.62 7.60 7.50 7.48 7.46 6.00 5.99 5.97 5.97 5.67 5.66 5.65 5.65 5.47 5.46 2.98 2.96 2.94 2.92 2.61 2.60 2.58 2.55 2.49 2.49 2.47 2.47 2.42 2.37 2.18 2.16 2.15 2.11 2.08 2.07 1.89 1.88 1.86 1.85 1.85 1.75 1.74 1.73 1.72 1.70 1.69 1.67 1.27 1.25 1.24 1.22 1.20 1.14 1.11 1.08 1.07
N
8fa
¹H NMR (400 MHz, CDCl₃)
1.00 1.03 1.02 1.00 0.98 1.00 1.00 1.00 3.44 12.13 2.30 6.08
10.0 9.5 9.0 8.5 8.0 7.5 7.0 6.5 6.0 5.5 5.0 4.5 4.0 3.5 3.0 2.5 2.0 1.5 1.0 0.5 0.0 -0.5 -1.0
f1 (ppm)

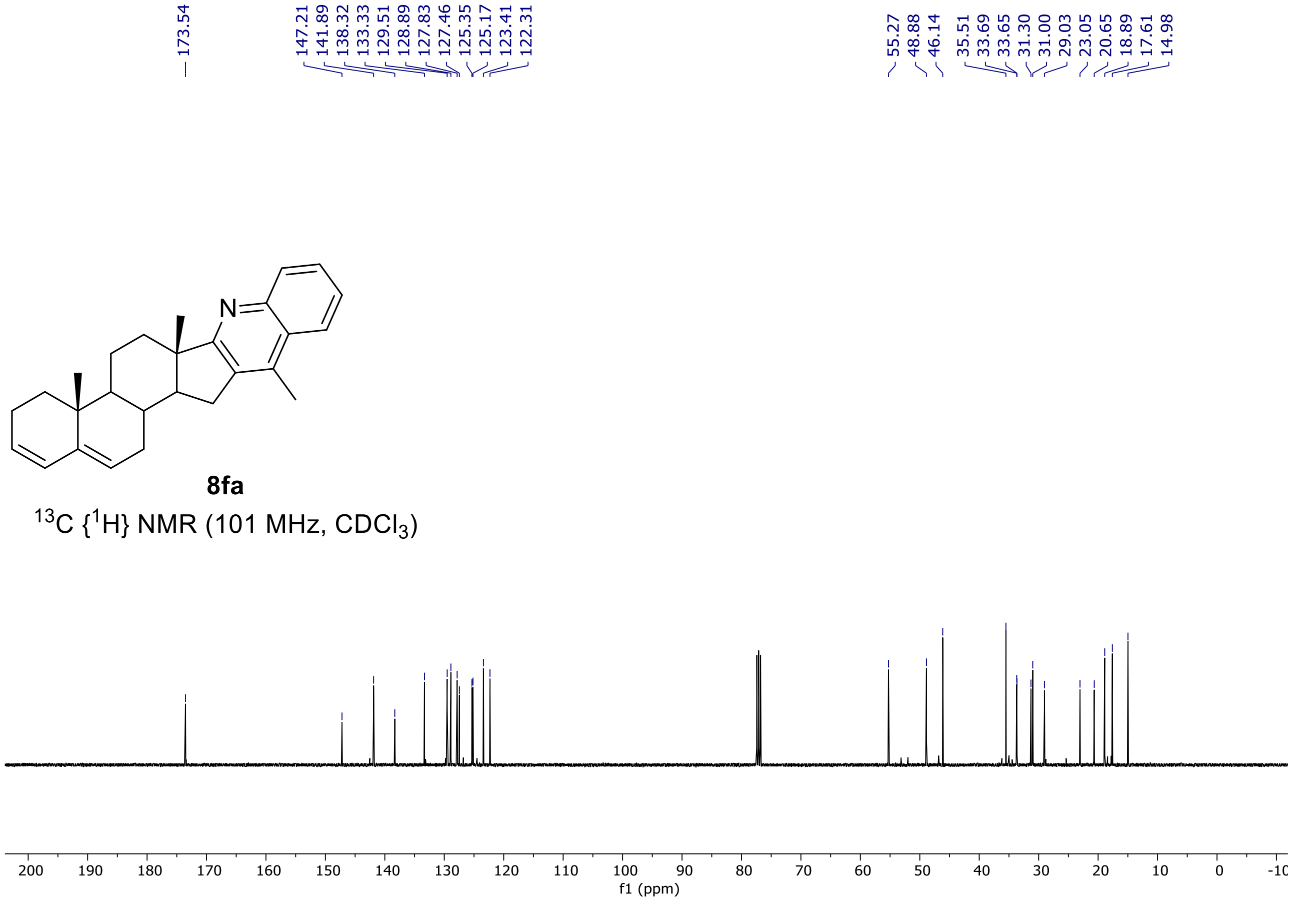
173.54
147.21 141.89 138.32 133.33 129.51 128.89 127.83 127.46 125.35 125.17 123.41 122.31
55.27 48.88 46.14 35.51 33.69 33.65 31.30 31.00 29.03 23.05 20.65 18.89 17.61 14.98
N
8fa
¹³C {¹H} NMR (101 MHz, CDCl₃)
200 190 180 170 160 150 140 130 120 110 100 90 80 70 60 50 40 30 20 10 0 -10
f1 (ppm)

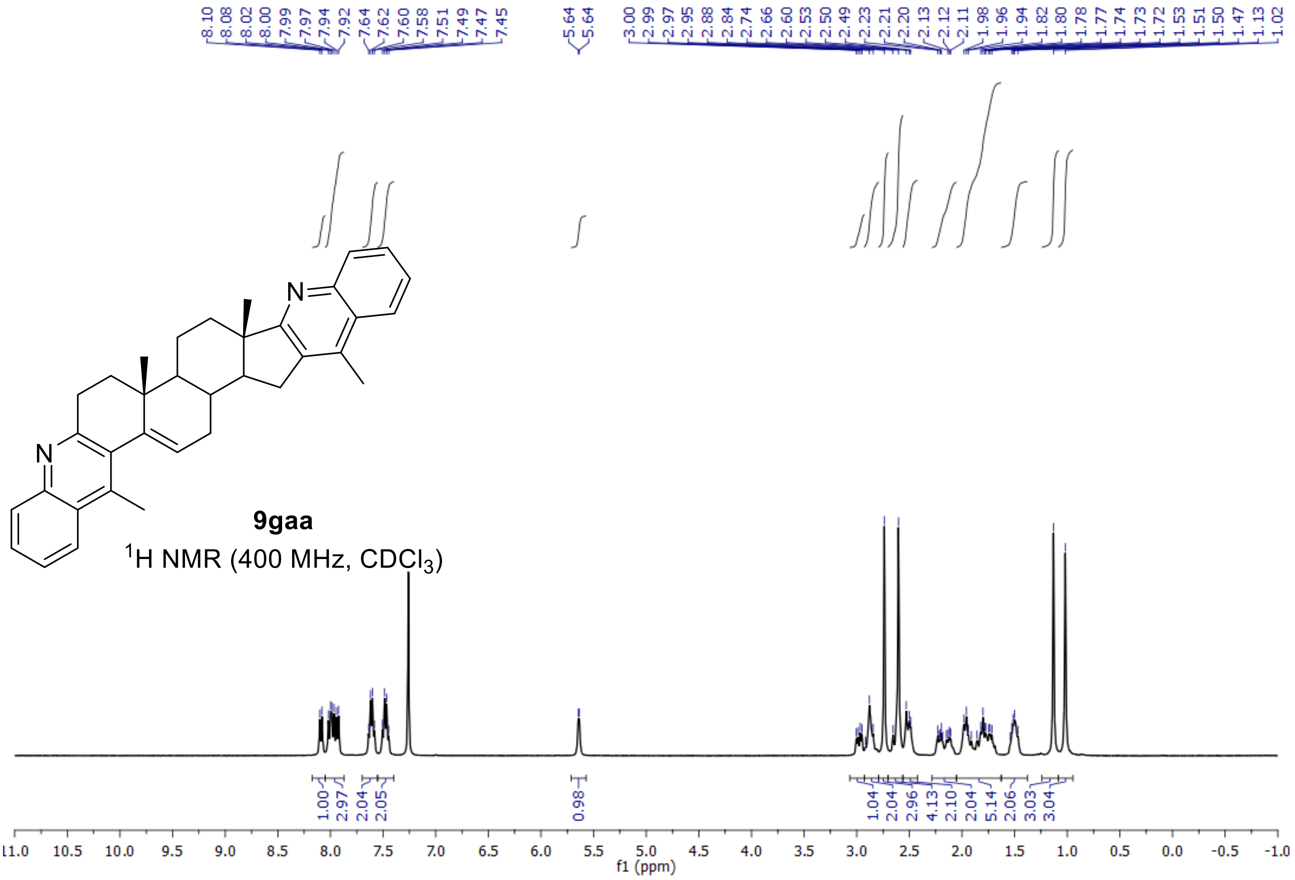

8.10 8.08 8.02 8.00 7.99 7.97 7.94 7.92 7.64 7.62 7.60 7.58 7.51 7.49 7.47 7.45
5.64 5.64
3.00 2.99 2.97 2.95 2.88 2.84 2.74 2.66 2.60 2.53 2.50 2.49 2.23 2.21 2.20 2.13 2.12 2.11 1.98 1.96 1.94 1.82 1.80 1.78 1.77 1.74 1.73 1.72 1.53 1.51 1.50 1.47 1.13 1.02
N
N
9gaa
1H NMR (400 MHz, CDCl3)
1.00 2.97 2.04 2.05 0.98 1.04 2.04 2.96 4.13 2.10 2.04 5.14 2.06 3.03 3.04
11.0 10.5 10.0 9.5 9.0 8.5 8.0 7.5 7.0 6.5 6.0 5.5 5.0 4.5 4.0 3.5 3.0 2.5 2.0 1.5 1.0 0.5 0.0 -0.5 -1.0
f1 (ppm)

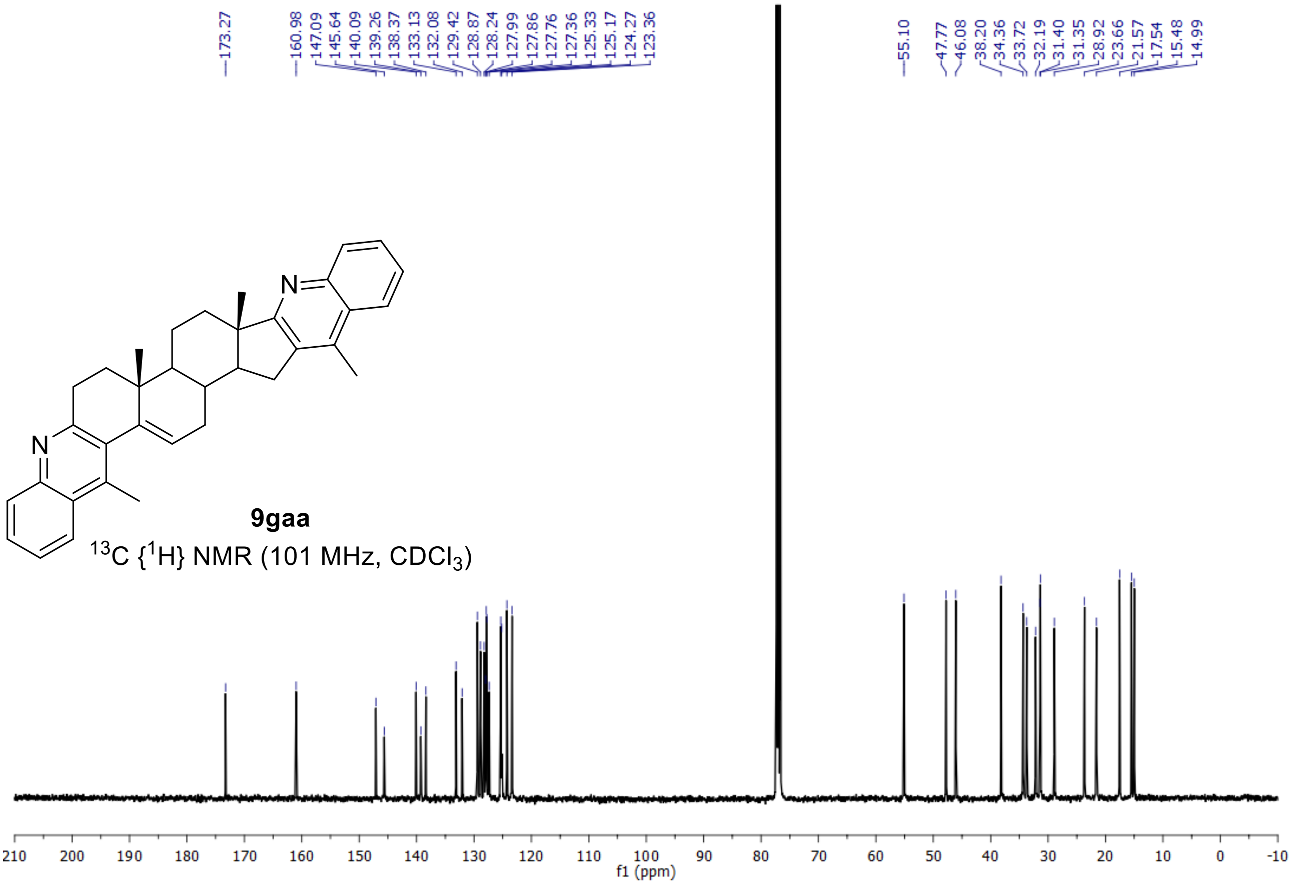

173.27
160.98 147.09 145.64 140.09 139.26 138.37 133.13 132.08 129.42 128.87 128.24 127.99 127.86 127.76 127.36 125.33 125.17 124.27 123.36
55.10 47.77 46.08 38.20 34.36 33.72 32.19 31.40 31.35 28.92 23.66 21.57 17.54 15.48 14.99
N
N
9gaa
13C {1H} NMR (101 MHz, CDCl3)
210 200 190 180 170 160 150 140 130 120 110 100 90 80 70 60 50 40 30 20 10 0 -10
f1 (ppm)

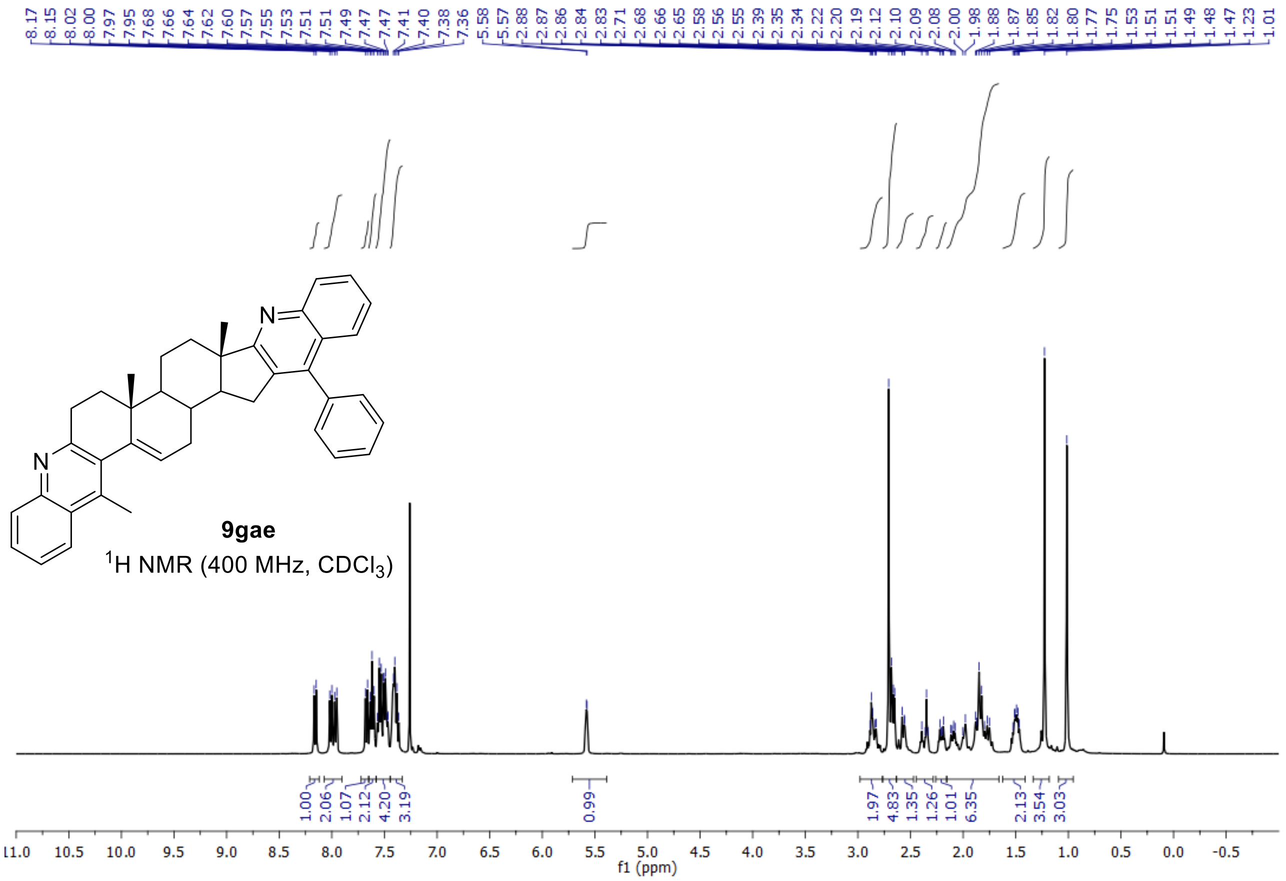
8.17 8.15 8.02 8.00 7.97 7.95 7.68 7.66 7.64 7.62 7.60 7.57 7.55 7.53 7.51 7.51 7.49 7.47 7.47 7.41 7.40 7.38 7.36 5.58 5.57 2.88 2.87 2.86 2.84 2.83 2.71 2.68 2.66 2.65 2.58 2.56 2.55 2.39 2.35 2.34 2.22 2.20 2.19 2.12 2.10 2.09 2.08 2.00 1.98 1.88 1.87 1.85 1.82 1.80 1.77 1.75 1.53 1.51 1.51 1.49 1.48 1.47 1.23 1.01
N
N
9gae
^{1}H NMR (400 MHz, CDCl$_3$)
1.00 2.06 1.07 2.12 4.20 3.19 0.99 1.97 4.83 1.35 1.26 1.01 6.35 2.13 3.54 3.03
11.0 10.5 10.0 9.5 9.0 8.5 8.0 7.5 7.0 6.5 6.0 5.5 5.0 4.5 4.0 3.5 3.0 2.5 2.0 1.5 1.0 0.5 0.0 -0.5
f1 (ppm)

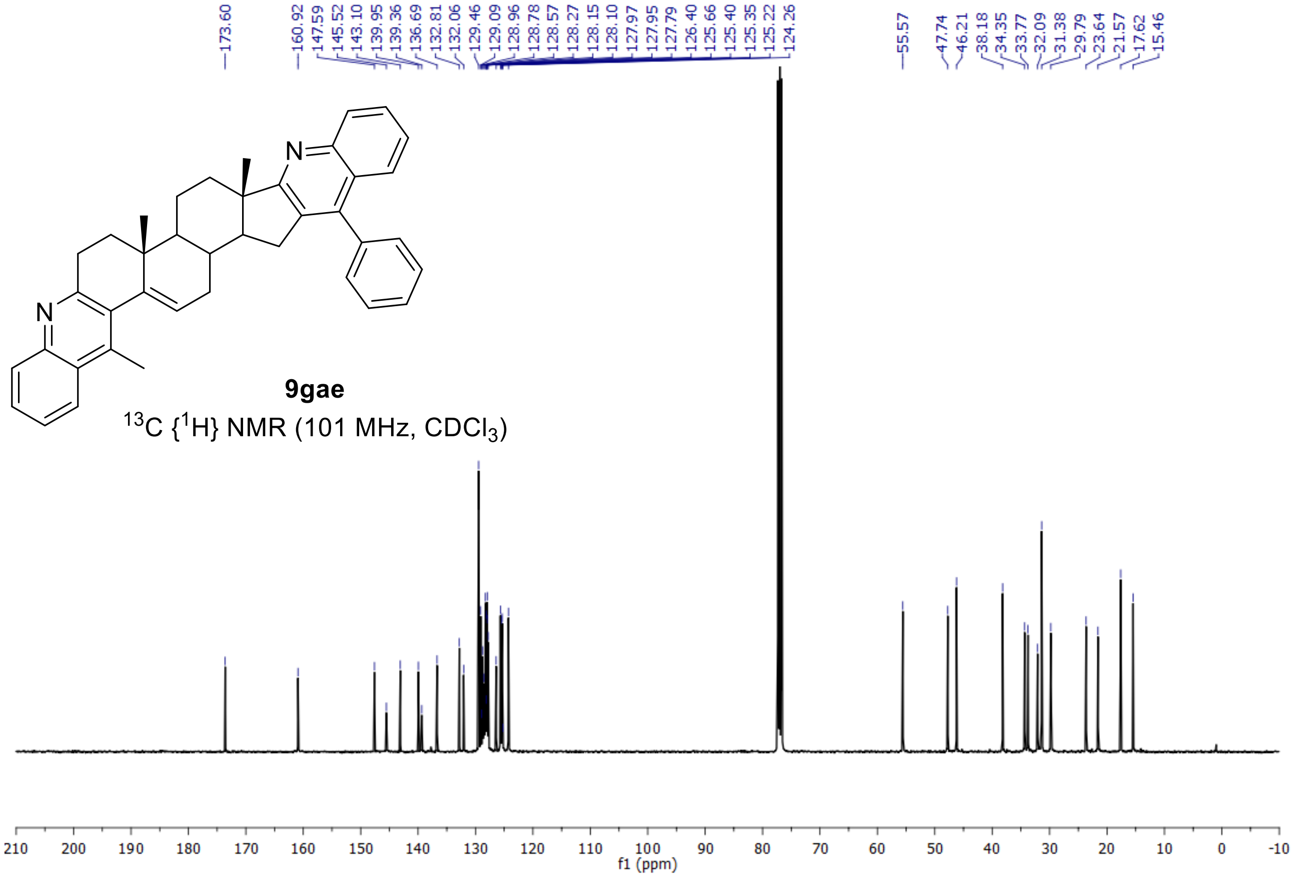
173.60 160.92 147.59 145.52 143.10 139.95 139.36 136.69 132.81 132.06 129.46 129.09 128.96 128.78 128.57 128.27 128.15 128.10 127.97 127.95 127.79 126.40 125.66 125.40 125.35 125.22 124.26 55.57 47.74 46.21 38.18 34.35 33.77 32.09 31.38 29.79 23.64 21.57 17.62 15.46
N
N
9gae
^{13}C {^{1}H} NMR (101 MHz, CDCl$_3$)
210 200 190 180 170 160 150 140 130 120 110 100 90 80 70 60 50 40 30 20 10 0 -10
f1 (ppm)

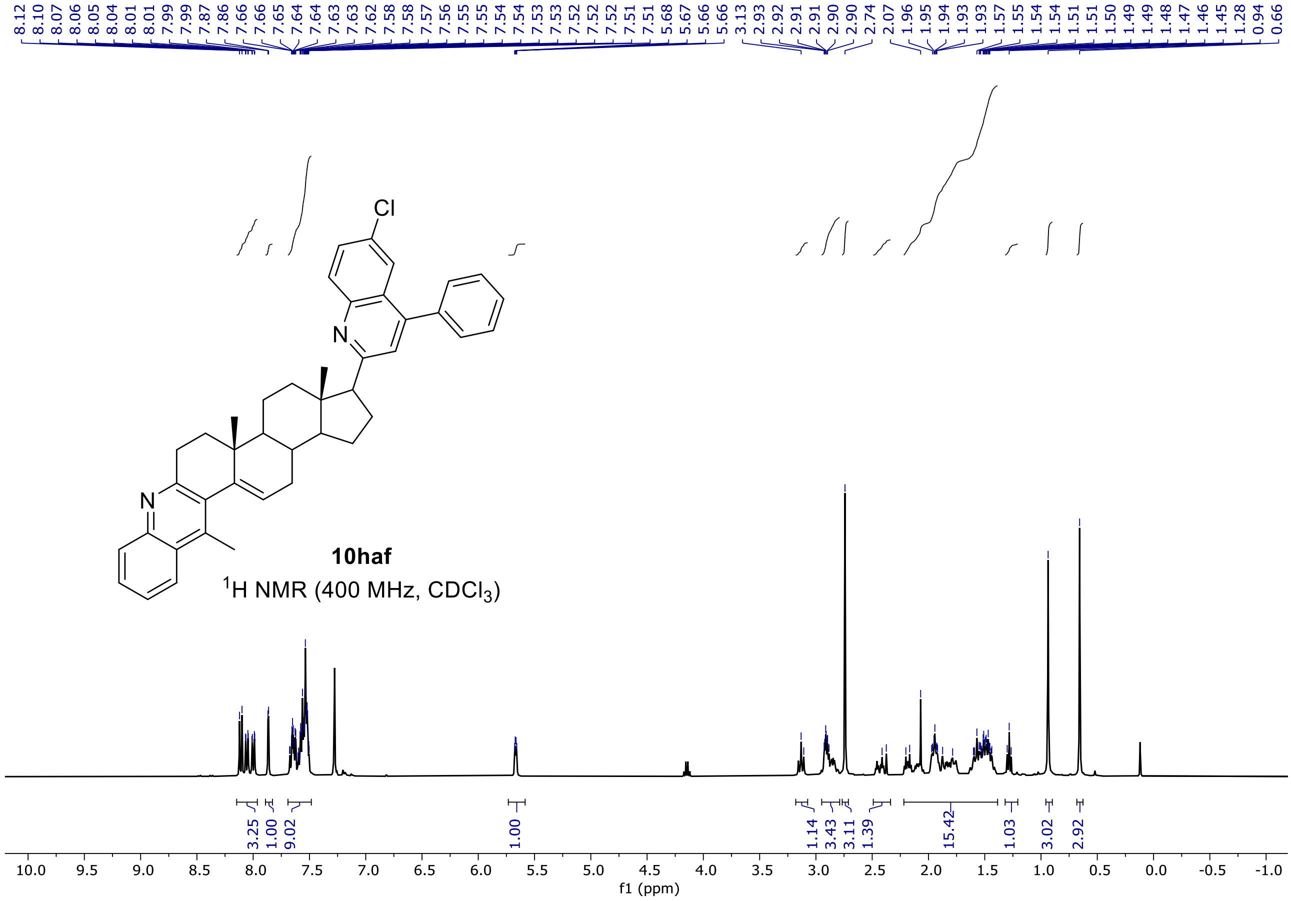
8.12 8.10 8.07 8.06 8.05 8.04 8.01 8.01 7.99 7.99 7.87 7.86 7.66 7.66 7.65 7.64 7.64 7.63 7.63 7.62 7.58 7.58 7.57 7.56 7.55 7.55 7.54 7.54 7.53 7.53 7.52 7.52 7.52 7.51 7.51 5.68 5.67 5.66 5.66 3.13 2.93 2.92 2.91 2.91 2.90 2.90 2.74 2.07 1.96 1.95 1.94 1.93 1.93 1.57 1.55 1.54 1.54 1.51 1.51 1.50 1.49 1.49 1.48 1.47 1.46 1.45 1.28 0.94 0.66
Cl
N
N
10haf
1H NMR (400 MHz, CDCl3)
3.25 1.00 9.02 1.00 1.14 3.43 3.11 1.39 15.42 1.03 3.02 2.92
10.0 9.5 9.0 8.5 8.0 7.5 7.0 6.5 6.0 5.5 5.0 4.5 4.0 3.5 3.0 2.5 2.0 1.5 1.0 0.5 0.0 -0.5 -1.0
f1 (ppm)

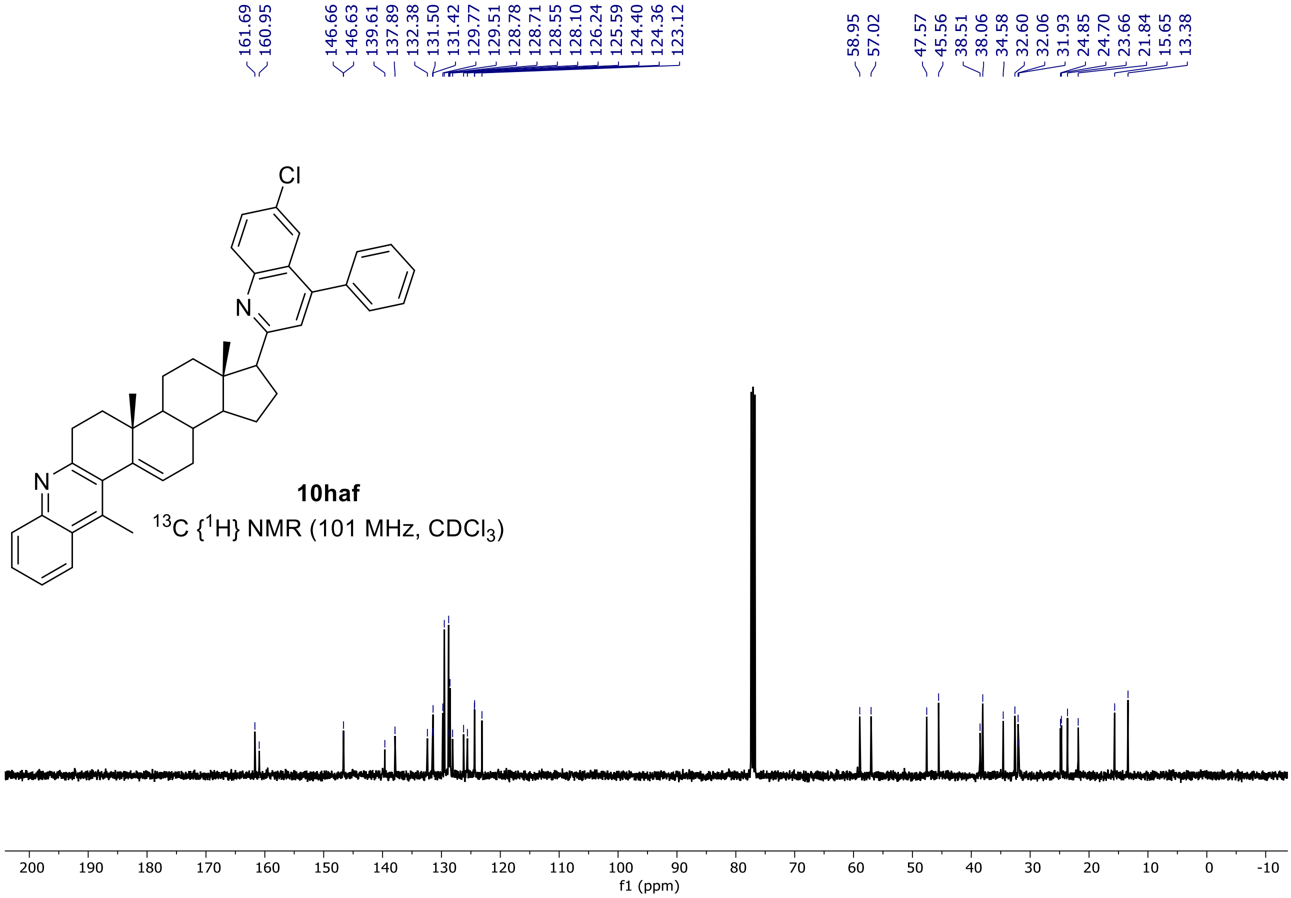
161.69 160.95 146.66 146.63 139.61 137.89 132.38 131.50 131.42 129.77 129.51 128.78 128.71 128.55 128.10 126.24 125.59 124.40 124.36 123.12 58.95 57.02 47.57 45.56 38.51 38.06 34.58 32.60 32.06 31.93 24.85 24.70 23.66 21.84 15.65 13.38
Cl
N
N
10haf
13C {1H} NMR (101 MHz, CDCl3)
200 190 180 170 160 150 140 130 120 110 100 90 80 70 60 50 40 30 20 10 0 -10
f1 (ppm)

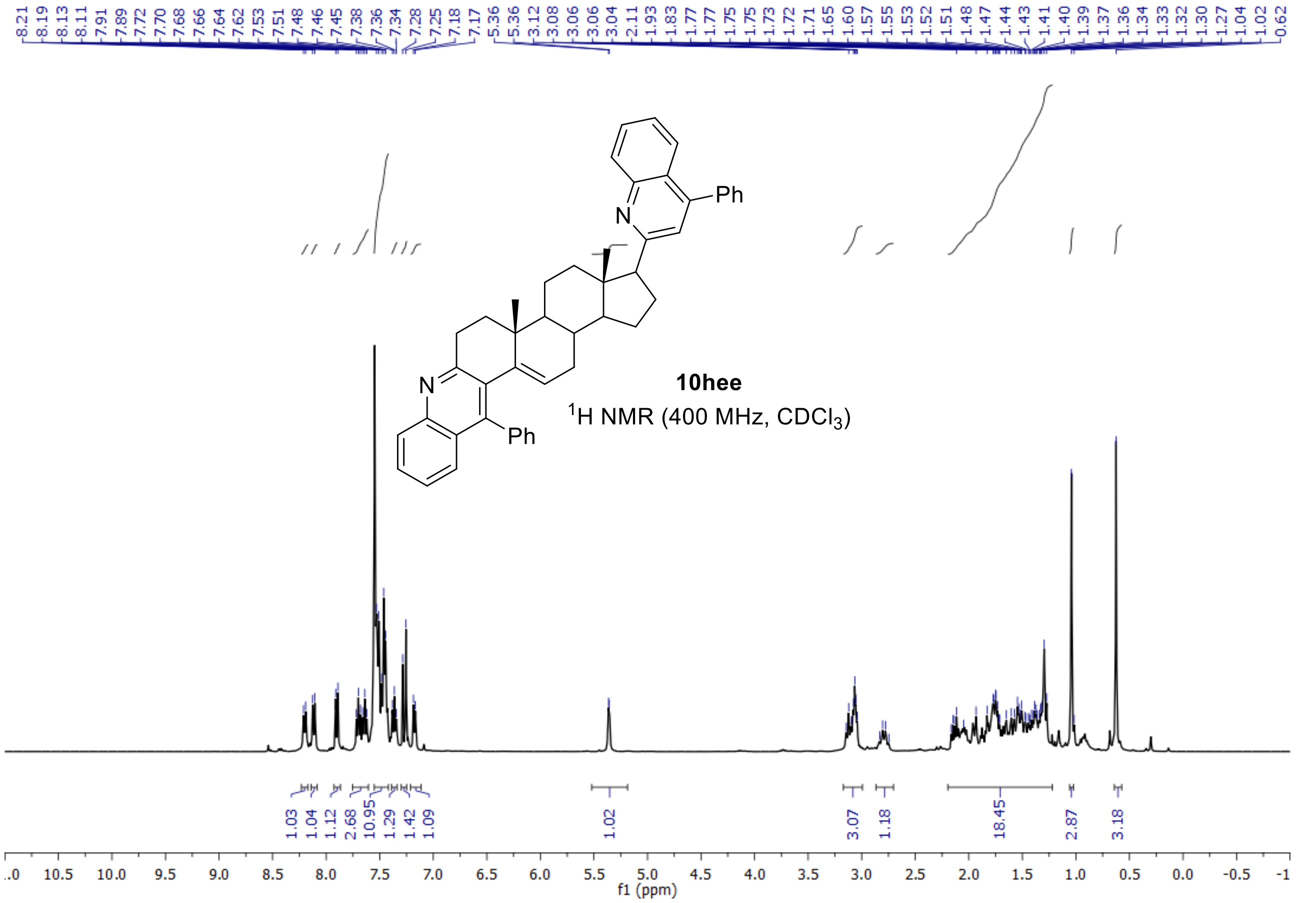
10hee
^{1}H NMR (400 MHz, CDCl$_3$)
f1 (ppm)

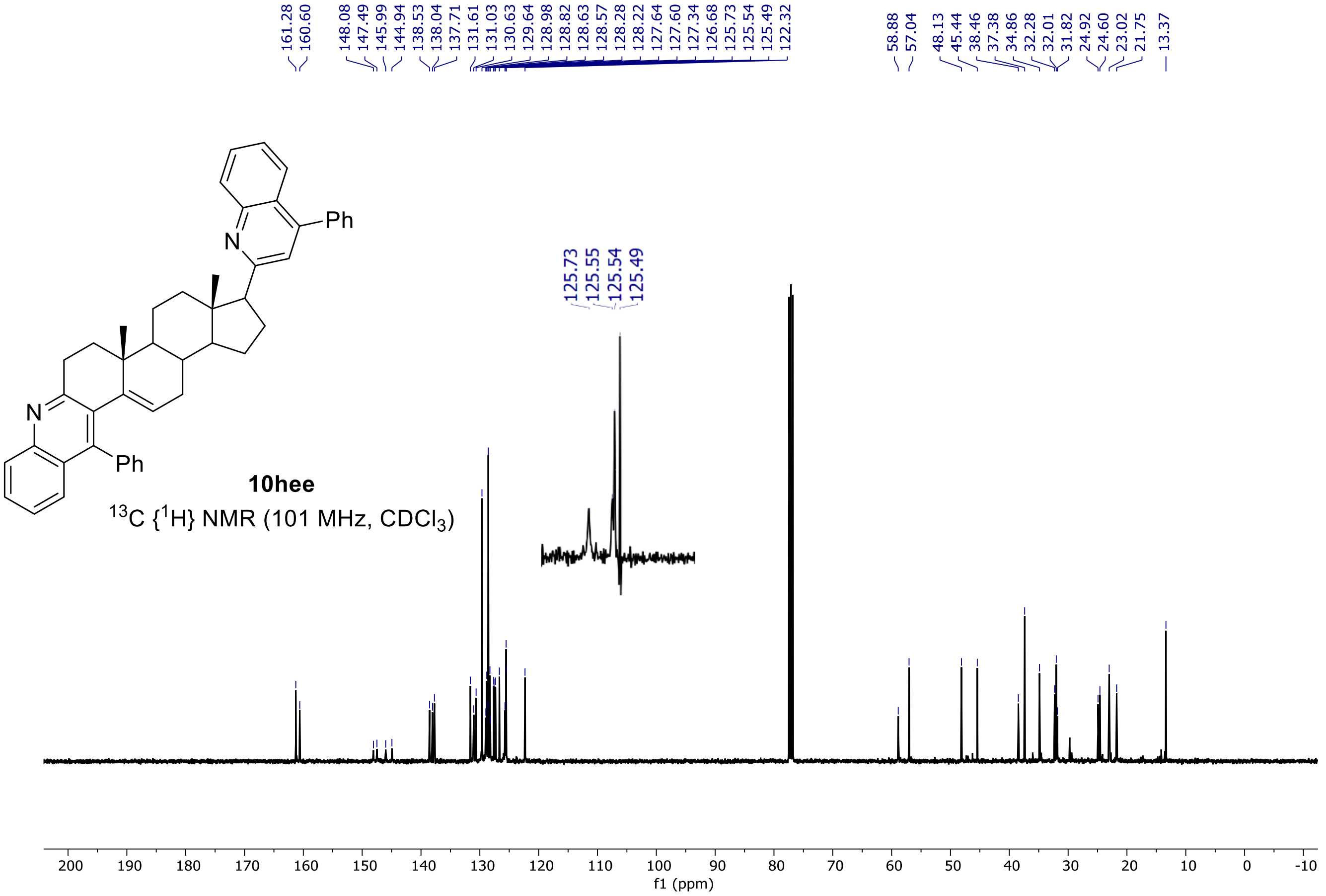
10hee
^{13}C {^{1}H} NMR (101 MHz, CDCl$_3$)
f1 (ppm)

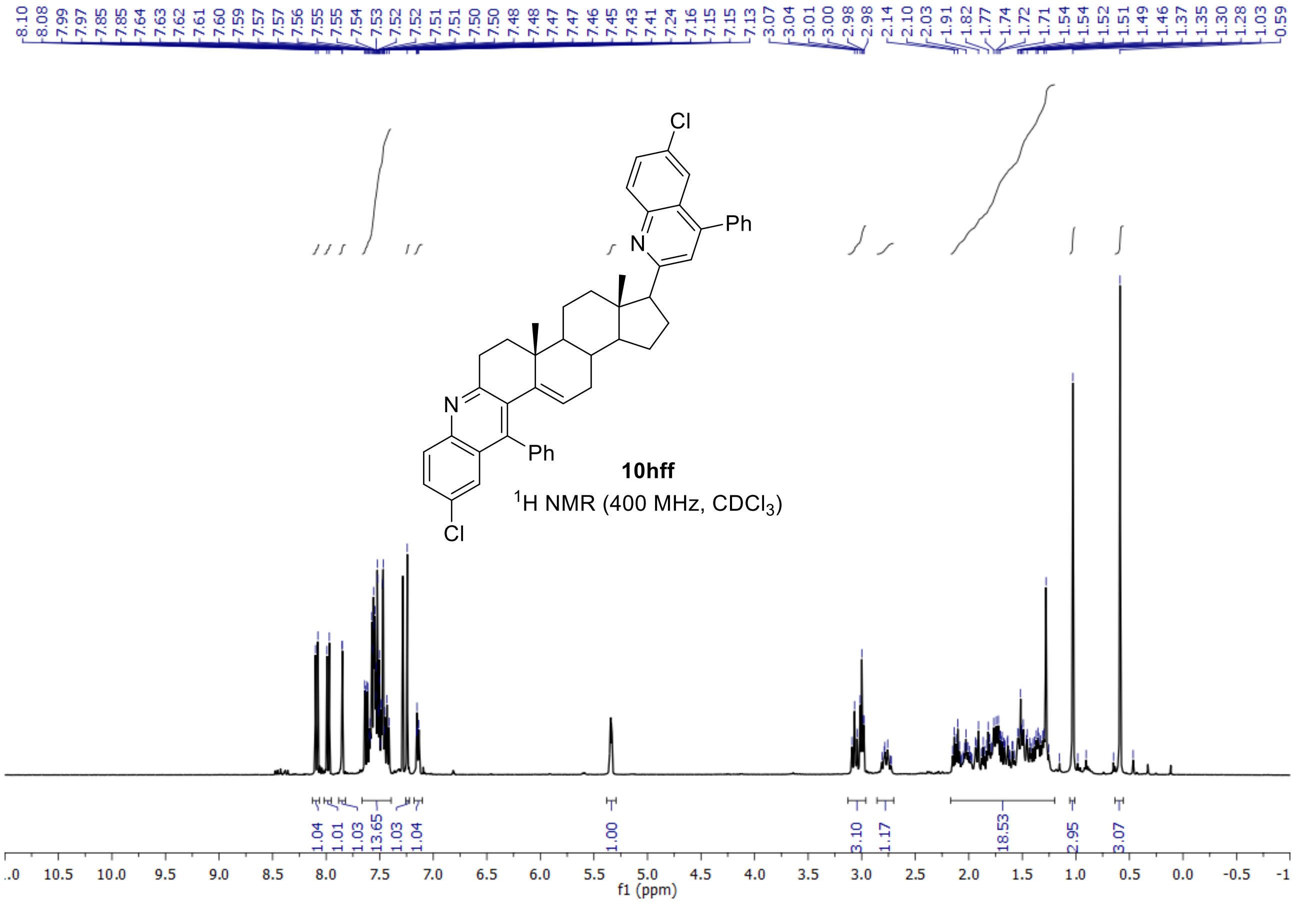
8.10 8.08 7.99 7.97 7.85 7.85 7.64 7.63 7.62 7.61 7.60 7.59 7.57 7.57 7.56 7.55 7.55 7.54 7.53 7.52 7.52 7.51 7.50 7.50 7.48 7.47 7.47 7.46 7.45 7.43 7.41 7.24 7.16 7.15 7.15 7.13 3.07 3.04 3.01 3.00 2.98 2.98 2.14 2.10 2.03 1.91 1.82 1.77 1.74 1.72 1.71 1.54 1.54 1.52 1.51 1.49 1.46 1.37 1.35 1.30 1.28 1.03 0.59
Cl
Ph
N
N
Ph
Cl
10hff
1H NMR (400 MHz, CDCl3)
1.04 1.01 1.03 13.65 1.03 1.04 1.00 3.10 1.17 18.53 2.95 3.07
f1 (ppm)

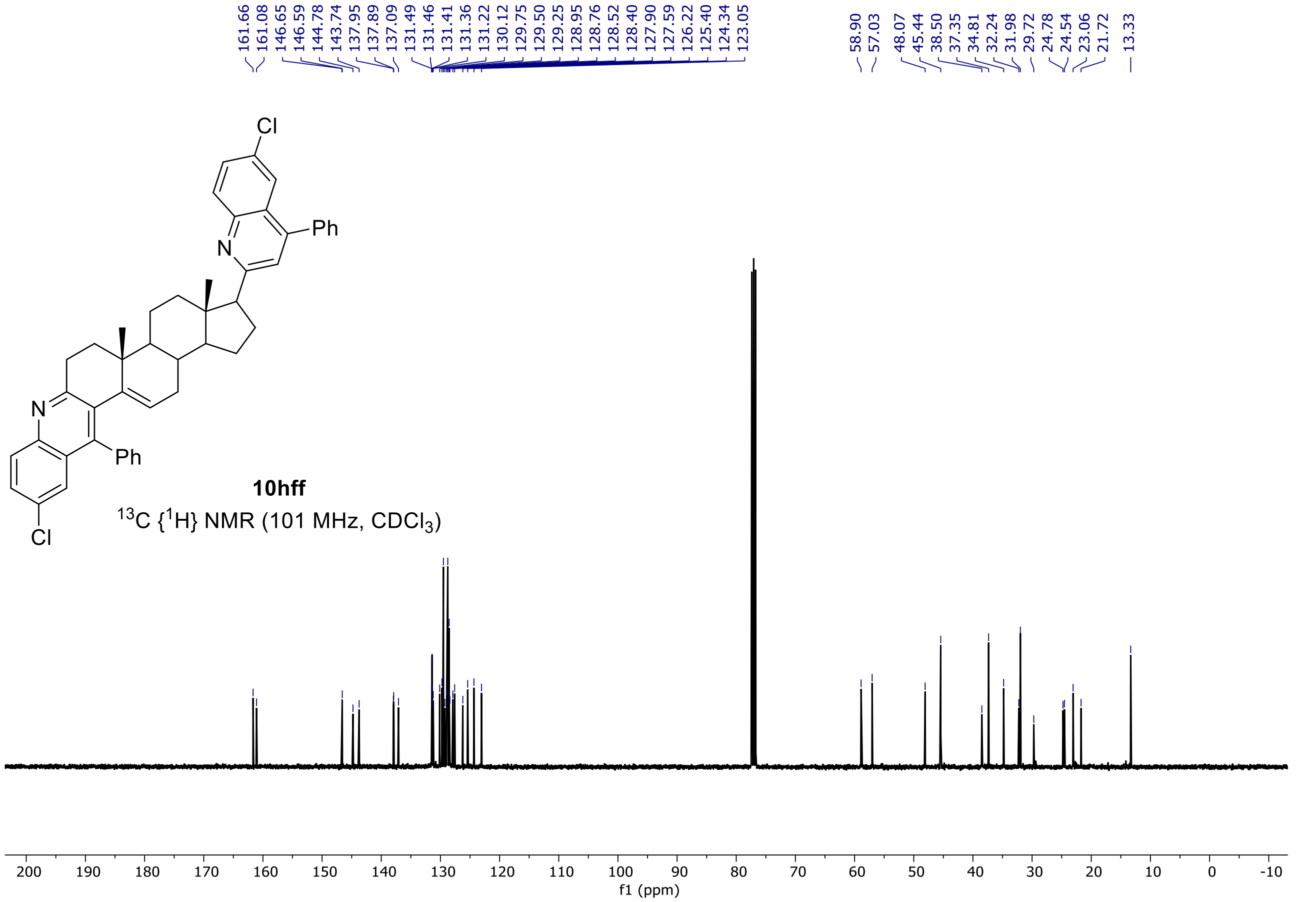
161.66 161.08 146.65 146.59 144.78 143.74 137.95 137.89 137.09 131.49 131.46 131.41 131.36 131.22 130.12 129.75 129.50 129.25 128.95 128.76 128.52 128.40 127.90 127.59 126.22 125.40 124.34 123.05 58.90 57.03 48.07 45.44 38.50 37.35 34.81 32.24 31.98 29.72 24.78 24.54 23.06 21.72 13.33
Cl
Ph
N
N
Ph
Cl
10hff
13C {1H} NMR (101 MHz, CDCl3)
f1 (ppm)

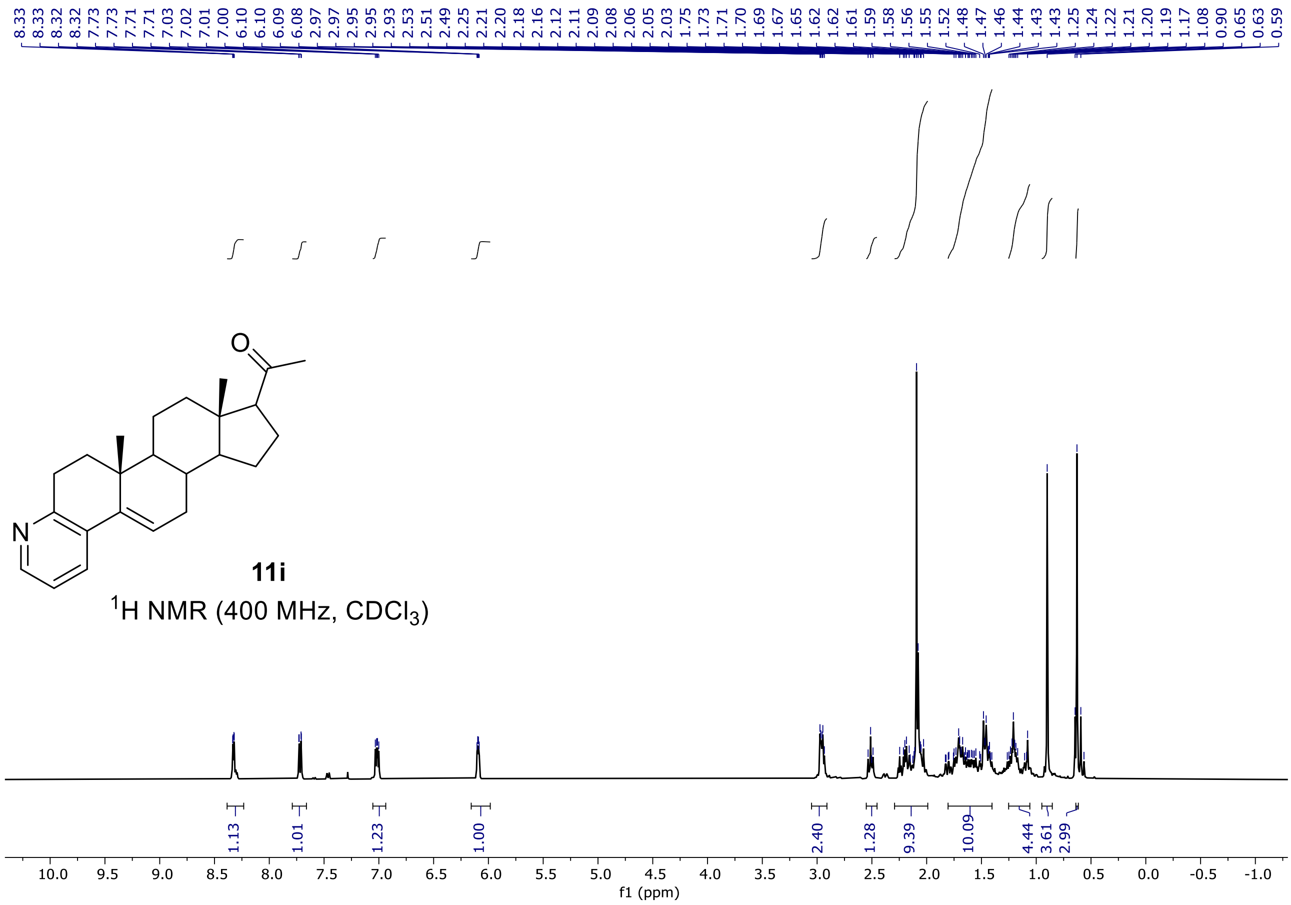
8.33 8.33 8.32 8.32 7.73 7.73 7.71 7.71 7.03 7.02 7.01 7.00 6.10 6.10 6.09 6.08 2.97 2.97 2.95 2.93 2.53 2.51 2.49 2.25 2.21 2.20 2.18 2.16 2.12 2.11 2.09 2.08 2.06 2.05 2.03 1.75 1.73 1.71 1.70 1.69 1.67 1.65 1.62 1.61 1.59 1.58 1.56 1.55 1.52 1.48 1.47 1.46 1.44 1.43 1.25 1.24 1.22 1.21 1.20 1.19 1.17 1.08 0.90 0.65 0.63 0.59
O
N
11i
^{1}H NMR (400 MHz, $CDCl_3$)
1.13 1.01 1.23 1.00 2.40 1.28 9.39 10.09 4.44 3.61 2.99
f1 (ppm)

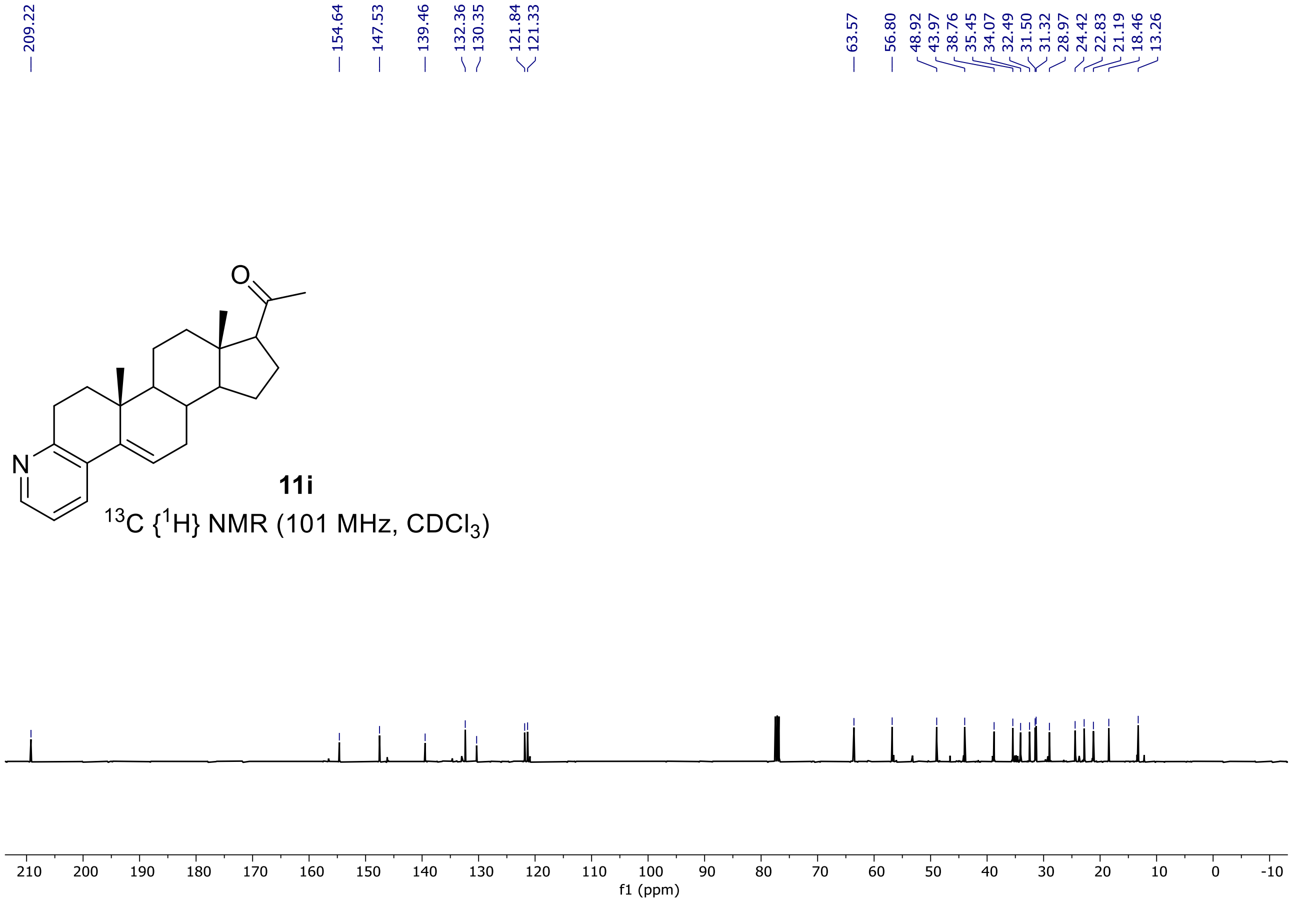
209.22 154.64 147.53 139.46 132.36 130.35 121.84 121.33 63.57 56.80 48.92 43.97 38.76 35.45 34.07 32.49 31.50 31.32 28.97 24.42 22.83 21.19 18.46 13.26
O
N
11i
^{13}C {^{1}H} NMR (101 MHz, $CDCl_3$)
f1 (ppm)

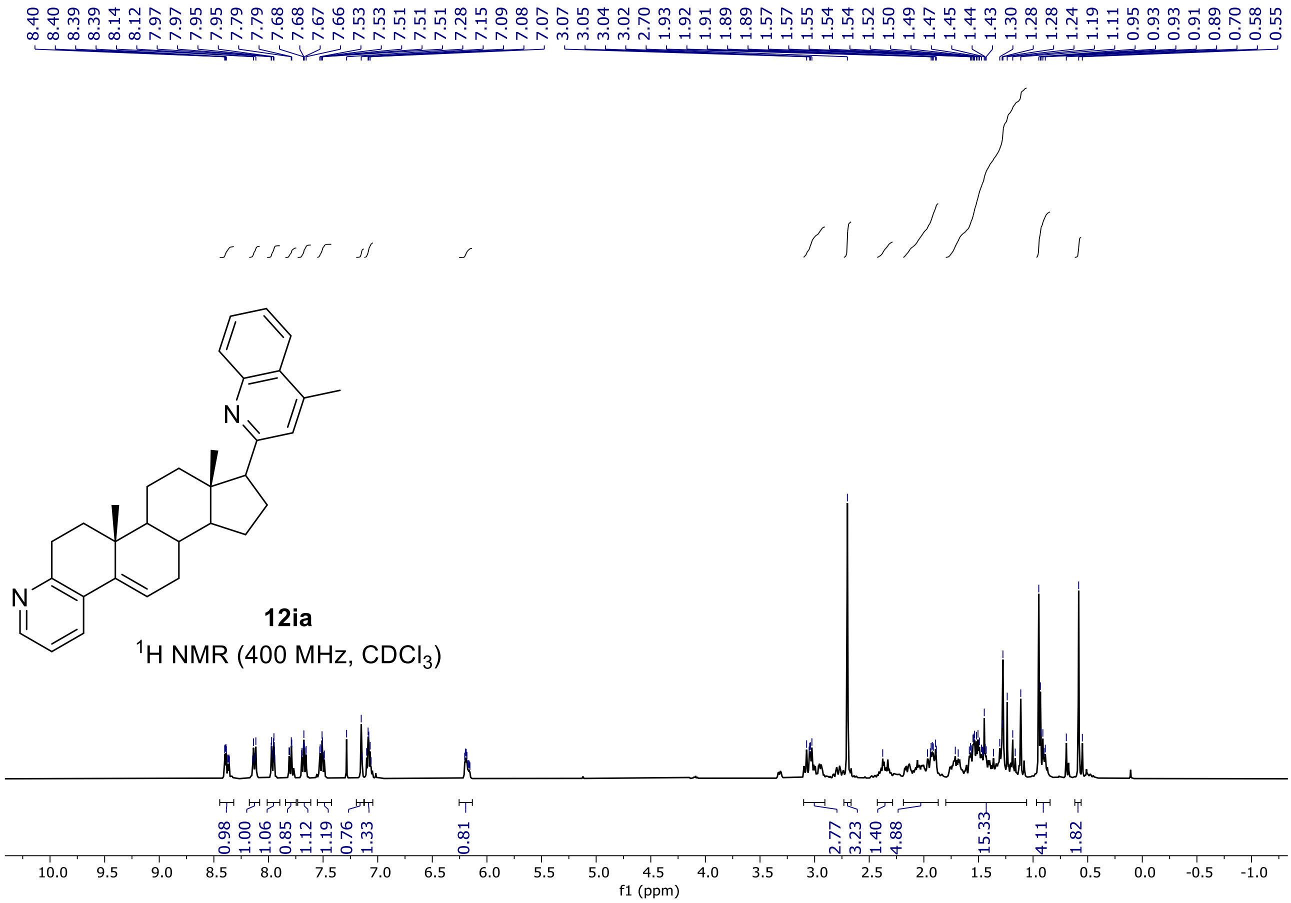
8.40 8.40 8.39 8.39 8.14 8.12 7.97 7.97 7.95 7.95 7.79 7.79 7.68 7.68 7.67 7.66 7.53 7.53 7.51 7.51 7.51 7.28 7.15 7.09 7.08 7.07 3.07 3.05 3.04 3.02 2.70 1.93 1.92 1.91 1.89 1.89 1.57 1.57 1.55 1.54 1.54 1.52 1.50 1.49 1.47 1.45 1.44 1.43 1.30 1.28 1.28 1.24 1.19 1.11 0.95 0.93 0.93 0.91 0.89 0.70 0.58 0.55
N
12ia
^{1}H NMR (400 MHz, CDCl$_3$)
0.98 1.00 1.06 0.85 1.12 1.19 0.76 1.33 0.81 2.77 3.23 1.40 4.88 15.33 4.11 1.82
10.0 9.5 9.0 8.5 8.0 7.5 7.0 6.5 6.0 5.5 5.0 4.5 4.0 3.5 3.0 2.5 2.0 1.5 1.0 0.5 0.0 -0.5 -1.0
f1 (ppm)

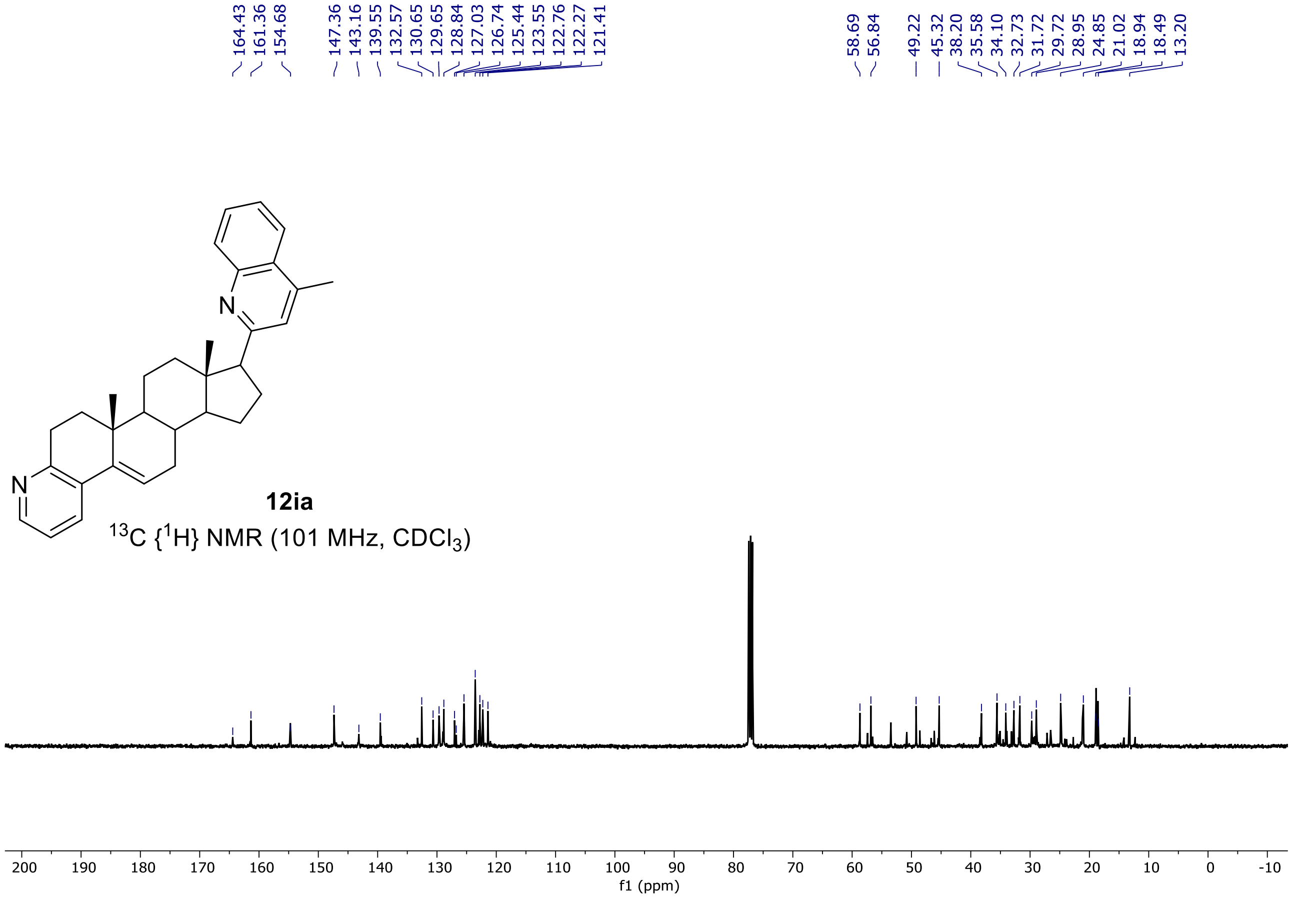
164.43 161.36 154.68 147.36 143.16 139.55 132.57 130.65 129.65 128.84 127.03 126.74 125.44 123.55 122.76 122.27 121.41
58.69 56.84 49.22 45.32 38.20 35.58 34.10 32.73 31.72 29.72 28.95 24.85 21.02 18.94 18.49 13.20
N
12ia
^{13}C {^{1}H} NMR (101 MHz, CDCl$_3$)
200 190 180 170 160 150 140 130 120 110 100 90 80 70 60 50 40 30 20 10 0 -10
f1 (ppm)

Computational Data

Calculations were performed at the CAM-B3LYP/6-311+G* level of theory with the Gaussian program.[5]

The cartesian coordinates of the structures are below reported.

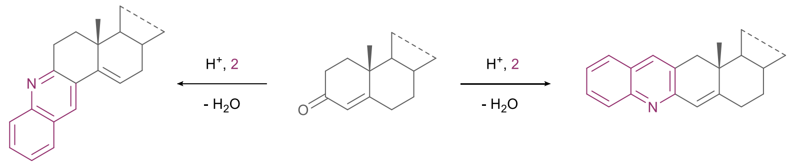

Angular Products 5 Δ^4-Ketosteroid Linear Products 6

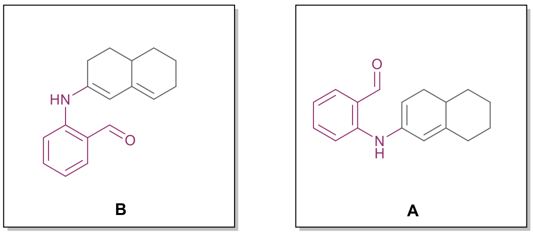

A

C	-2.113493	0.471572	7.077959
C	-2.742290	1.566501	6.470034
C	-3.273445	2.577198	7.293182
C	-3.213665	2.444854	8.685676
C	-2.600128	1.360570	9.271800
C	-2.042591	0.377337	8.452520
N	-2.907223	1.606194	5.088271
C	-1.982762	1.209352	4.108021
C	-2.561187	1.023295	2.770987
C	-1.807258	0.930210	1.673064
C	-0.296187	0.955995	1.787529
C	0.173418	0.504743	3.175269
C	-0.671580	1.030701	4.300387
C	-2.403484	0.958198	0.291540
C	-1.681079	0.049254	-0.702346
C	-0.183859	0.339875	-0.695821
C	0.393786	0.121598	0.698287
C	-3.832593	3.823092	6.741391
O	-4.434877	4.649409	7.386026

H	0.334133	-0.298813	-1.416785
H	0.291796	-0.939557	0.955115
H	1.466679	0.336637	0.710035
H	0.000450	2.007447	1.639313
H	-3.470319	0.725109	0.339984
H	-2.332321	1.989628	-0.081539
H	-1.850484	-1.000218	-0.435817
H	-2.099726	0.189457	-1.702712
H	-3.645551	1.031564	2.686817
H	-0.217055	1.191832	5.270012
H	0.166512	-0.595362	3.202681
H	1.217280	0.796399	3.316556
H	-3.682470	2.145564	4.742890
H	-3.646428	3.237850	9.284260
H	-2.547172	1.272331	10.350330
H	-1.562647	-0.487690	8.897723
H	-1.714963	-0.319095	6.456151
H	-3.642951	4.014822	5.667907
H	-0.009825	1.373904	-1.017677

B

C	-5.852999	1.912046	2.735384
C	-5.585441	0.982083	3.747793
C	-6.669202	0.342925	4.378247
C	-7.979643	0.684752	4.026368
C	-8.231864	1.607492	3.035257
C	-7.154462	2.212459	2.386979
N	-4.279072	0.748806	4.178292
C	-3.105450	0.746183	3.419929
C	-3.061629	0.571798	2.091012
C	-1.826661	0.654475	1.317989
C	-0.582341	1.085367	2.068799
C	-0.606736	0.509218	3.483462

C	-1.857402	0.945753	4.237390
C	-1.797810	0.366094	0.011514
C	-0.566991	0.421972	-0.845902
C	0.591677	1.137654	-0.153682
C	0.688716	0.716509	1.308715
C	-6.474567	-0.743625	5.354356
O	-7.351494	-1.213077	6.040976
H	1.529531	0.935974	-0.678238
H	0.842621	-0.367952	1.367216
H	1.555002	1.181833	1.789119
H	-0.618313	2.181257	2.161977
H	-2.719271	0.050870	-0.474009
H	-0.267757	-0.600545	-1.113418
H	-0.799895	0.911441	-1.797628
H	-3.975149	0.365261	1.543763
H	-1.942430	0.385301	5.176580
H	-1.783497	2.001115	4.526561
H	0.287108	0.819881	4.032067
H	-0.578974	-0.583974	3.424460
H	-4.160439	0.522690	5.151009
H	-8.786887	0.180001	4.544075
H	-9.249450	1.857034	2.759520
H	-7.335487	2.946123	1.608742
H	-5.029377	2.418633	2.250240
H	-5.454913	-1.168728	5.417409
H	0.433378	2.220773	-0.203941

References

1) Arcadi, A.; Bernocchi, E.; Burini, A.; Cacchi, S.; Marinelli, F.; Pietroni, B. The Palladium-Tributylammonium Formate Reagent in the Stereoselective Hydrogenation, and Stereo- and Regioselective Hydroarylation of Alkyl 4-Hydroxy-2-Alkynoates: A Route to Substituted Butenolides. *Tetrahedron*, **1988**, 44, 481-490.

2) Manhas, M.S.; Mc Coy, J.R. Some steroid heterocycle s*via* enamines. *J.Chem.Soc.(C)*,**1969**,1419-1422.

3) Momoli, C.; Morlacci, V.; Chiarini, M.; Palombi, L.; Arcadi, A. Friedländer-Type Reaction of 4-Cholesten-3- one with 2′-Aminoacetophenone: Angular versus Linear Quinoline-Fused Steroids. *Molbank* **2023**, 2023, M1712;

4) G. Abbiati, A. Arcadi, G. Bianchi, S. Di Giuseppe, F. Marinelli, E. Rossi, Sequential Amination/Annulation/Aromatization Reaction of Carbonyl Compounds and Propargylamine: A New One-Pot Approach to Functionalized Pyridines. *J. Org. Chem.* **2003**, *68*, 6959-6966

5) - Gaussian 16, Revision A.03: a) M. J. Frisch, G. W. Trucks, H. B. Schlegel, G. E. Scuseria, b) M. A. Robb, J. R. Cheeseman, G. Scalmani, V. Barone, c) G. A. Petersson, H. Nakatsuji, X. Li, M. Caricato, A. V. Marenich, d) J. Bloino, B. G. Janesko, R. Gomperts, B. Mennucci, H. P. Hratchian, e) J. V. Ortiz, A. F. Izmaylov, J. L. Sonnenberg, D. Williams-Young, f) F. Ding, F. Lipparini, F. Egidi, J. Goings, B. Peng, A. Petrone, g) T. Henderson, D. Ranasinghe, V. G. Zakrzewski, J. Gao, N. Rega, h) G. Zheng, W. Liang, M. Hada, M. Ehara, K. Toyota, R. Fukuda, i) J. Hasegawa, M. Ishida, T. Nakajima, Y. Honda, O. Kitao, H. Nakai, j) T. Vreven, K. Throssell, J. A. Montgomery, Jr., J. E. Peralta, k) F. Ogliaro, M. J. Bearpark, J. J. Heyd, E. N. Brothers, K. N. Kudin, l) V. N. Staroverov, T. A. Keith, R. Kobayashi, J. Normand, m) K. Raghavachari, A. P. Rendell, J. C. Burant, S. S. Iyengar, n) J. Tomasi, M. Cossi, J. M. Millam, M. Klene, C. Adamo, R. Cammi, o) J. W. Ochterski, R. L. Martin, K. Morokuma, O. Farkas, p) J. B. Foresman, and D. J. Fox, Gaussian, Inc., Wallingford CT, 2016.
